# Temporal and phylogenetic evolution of the sauropod dinosaur body plan

**DOI:** 10.1098/rsos.150636

**Published:** 2016-03-30

**Authors:** Karl T. Bates, Philip D. Mannion, Peter L. Falkingham, Stephen L. Brusatte, John R. Hutchinson, Alejandro Otero, William I. Sellers, Corwin Sullivan, Kent A. Stevens, Vivian Allen

**Affiliations:** 1 Department of Musculoskeletal Biology, Institute of Aging and Chronic Disease, University of Liverpool, The Apex Building, 6 West Derby Street, Liverpool L7 8TX, UK; 2 Department of Earth Science and Engineering, Imperial College London, South Kensington Campus, London SW7 2AZ, UK; 3 School of Natural Sciences and Psychology, Liverpool John Moores University, James Parsons Building, Bryon Street, Liverpool L3 3AF, UK; 4 School of GeoSciences, University of Edinburgh, Grant Institute, The King's Buildings, James Hutton Road, Edinburgh EH9 3FE, UK; 5 Department of Comparative Biomedical Sciences, Structure and Motion Laboratory, Royal Veterinary College, University of London, Hatfield, Hertfordshire AL9 7TA, UK; 6 CONICET – División Paleontología de Vertebrados, Museo de La Plata, Paseo del Bosque s/n, La Plata B1900FWA, Argentina; 7 Faculty of Life Sciences, University of Manchester, Michael Smith Building, Oxford Road, Manchester M13 9PT, UK; 8 Key Laboratory of Vertebrate Evolution and Human Origins, Institute of Vertebrate Paleontology and Paleoanthropology, Chinese Academy of Sciences, 142 Xizhimenwai Dajie, Beijing 100044, People's Republic of China; 9 Department of Computer and Information Science, University of Oregon, Eugene, OR 97403, USA

**Keywords:** biomechanics, computer modelling, centre-of-mass, body shape, phylogeny, gigantism

## Abstract

The colossal size and body plan of sauropod dinosaurs are unparalleled in terrestrial vertebrates. However, to date, there have been only limited attempts to examine temporal and phylogenetic patterns in the sauropod bauplan. Here, we combine three-dimensional computational models with phylogenetic reconstructions to quantify the evolution of whole-body shape and body segment properties across the sauropod radiation. Limitations associated with the absence of soft tissue preservation in fossils result in large error bars about mean absolute body shape predictions. However, applying any consistent skeleton : body volume ratio to all taxa does yield changes in body shape that appear concurrent with major macroevolutionary events in sauropod history. A caudad shift in centre-of-mass (CoM) in Middle Triassic Saurischia, associated with the evolution of bipedalism in various dinosaur lineages, was reversed in Late Triassic sauropodomorphs. A craniad CoM shift coincided with the evolution of quadrupedalism in the Late Triassic, followed by a more striking craniad shift in Late Jurassic–Cretaceous titanosauriforms, which included the largest sauropods. These craniad CoM shifts are strongly correlated with neck enlargement, a key innovation in sauropod evolution and pivotal to their gigantism. By creating a much larger feeding envelope, neck elongation is thought to have increased feeding efficiency and opened up trophic niches that were inaccessible to other herbivores. However, we find that relative neck size and CoM position are not strongly correlated with inferred feeding habits. Instead the craniad CoM positions of titanosauriforms appear closely linked with locomotion and environmental distributions, potentially contributing to the continued success of this group until the end-Cretaceous, with all other sauropods having gone extinct by the early Late Cretaceous.

## 1. Introduction

Sauropod dinosaurs were the dominant group of large herbivores in global terrestrial ecosystems throughout much of the Mesozoic [1,2]. Their gigantic body sizes, an order of magnitude greater than any living terrestrial animal, in combination with a body plan distinct among tetrapods (e.g. long muscular necks and tails; graviportal, columnar limbs) make them a unique group for studies of morphological and functional evolution through deep time [3]. In particular, the evolution of sauropods from relatively small-bodied bipedal, and possibly facultatively bipedal, ancestors into extremely large-bodied obligate quadrupeds involved fundamental changes to most aspects of their biology [3]. However, despite numerous studies linking changes in biodiversity, ecology and biomechanics to body size and shape [3–6], there is a clear lack of quantitative analysis of temporal and phylogenetic trends in the sauropod bauplan.

**Figure 1.**
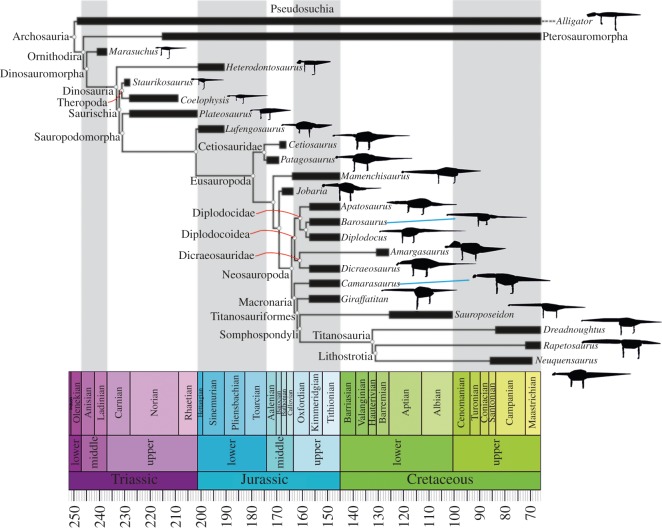
Time-calibrated phylogeny showing taxa included in this study (partly generated using [8]), with silhouettes of the convex hull volumetric models in left lateral view. Silhouettes not to scale.

Simple bone and body length segment ratios have been used to quantify aspects of body shape diversity across Sauropoda [7]. Studies that have sought to more directly quantify three-dimensional body shape in sauropods [5,6] have been hampered by small sample sizes. In particular very few Titanosauriformes, which dominated sauropod faunas throughout the Cretaceous [2], with derived members being the only sauropods to survive up to the end-Cretaceous mass extinction [1,2], have been subject to body shape analysis due to the absence of well-preserved specimens. The group includes famous taxa such as *Brachiosaurus*, as well as the largest known sauropods, such as the gigantic *Argentinosaurus* [1–3]. Therefore, we currently have very little understanding of how the unprecedented body plans of titanosauriforms contributed to their success in the latter half of the 150 million year evolutionary history of sauropods (figure 1).

**Figure 2.**
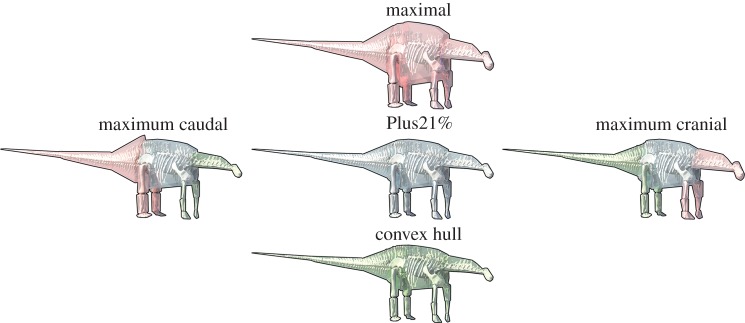
Reconstructed sauropod dinosaur (*Dicraeosaurus*) body volumes. We used an automated algorithm to produce an initial minimum convex hull volume (bottom model, green) around digitized fossil skeletons to minimize subjectivity [9,10]. Two geometrically similar expansions of this minimal volume were produced (‘Plus21%’ middle, grey (in accordance with [9]); ‘maximal’ top, red) from which we selected combinations of body segments that produced the most caudal (left) and cranial (right) CoM positions.

In this study, we attempt to rectify this by estimating parameters for overall body morphology (mass, centre-of-mass (CoM) and first mass moments (FMM, mass multiplied by CoM position)), both at whole-body and body segment levels for exemplar taxa covering the temporal and phylogenetic extent of the sauropod radiation (sauropods, basal sauropodomorphs and their immediate antecedents spanning the Middle Triassic through to the end-Cretaceous; figure 1) using automated computational volumetric techniques [9,10] (figure 2). Specimens of 17 sauropodomorph taxa and an additional five extinct and extant outgroup taxa were chosen (figure 1). Crucially, our analysis includes a number of Cretaceous titanosauriforms, made possible by recent discoveries of near-complete specimens and through careful sensitivity analysis of less complete taxa. Indeed, herein we conduct an exhaustive sensitivity analysis of numerous parameters associated with volumetric reconstruction (building on our previous work [9–14]), allowing us to quantitatively demonstrate keys areas of uncertainty in our models and subsequently to qualitatively gauge confidence in our ability to reconstruct macroevolutionary patterns within sauropodomorph dinosaurs.

To address temporal and phylogenetic patterns directly, rather than just using values for the studied sauropods, we mapped normalized estimated parameters from our volumetric models onto the evolutionary splitting events or nodes shown in figure 1 (based on [15]), using temporal branch lengths and a Brownian maximum-likelihood evolutionary model. This approach furthermore allows us to identify associations between morphological patterns in whole-body CoM and segment-specific parameters, and place changes in these fundamental biological properties in the context of existing hypotheses regarding functional, ecological and macroevolutionary patterns within sauropodomorphs.

## 2. Material and methods

### 2.1. Taxonomic coverage

Our sample of taxa (figure 1) covers the full temporal extent of the sauropod radiation (sauropods, basal sauropodomorphs and their immediate antecedents, spanning the Middle Triassic through to the end-Cretaceous). Phylogenetically, all major subclades are represented, with the exception of Rebbachisauridae. Very few sauropodomorphs are represented by individuals with highly complete skeletons. Indeed, volumetric reconstructions of dinosaurs in general rely heavily on composite skeletons produced by scaling elements from multiple individuals and estimating the dimensions of unpreserved elements using crude geometric proxies or reconstructions in cast/sculpted material. In the electronic supplementary material (S1), we review skeletal completeness in our sample of sauropodomorph taxa before exploring its impact on our results in a number of different ways through several sensitivity analyses (see below).

### 2.2. Volumetric reconstruction approach

Three-dimensional models of complete to near-complete skeletons of taxa (figure 1; see also electronic supplementary material, S1) were digitized using either long-range laser scanning [9–12], digital photogrammetry [16] or computed tomography scanning in the case of *Alligator*. The model of *Camarasaurus* was generated through computer-aided design approaches described in Stevens [17]. To quantify body proportions and overall body shape, CoM position and body segment masses were estimated from computer reconstructions of gross morphology built around digitized skeletons using a convex hulling approach [9,10]. Each three-dimensional skeletal model was posed in a standard reference posture, with the tail and neck extending horizontally and the limbs in a fully extended, vertical position (figure 2 and electronic supplementary material, movie S1). Models were then divided into standardized body segments and the minimum convex hull (enclosed volume) around each segment calculated using the Matlab (www.mathworks.com) qhull algorithm [9,10]. This mathematical approach of tight fitting three-dimensional convex polygons to each body segment minimizes subjectivity in body volume reconstruction. In addition, the extent of an object's convex hull is dictated solely by its geometric extremes, which minimizes impact of reconstructed (i.e. missing) skeletal components in the mounted skeletons (see the electronic supplementary material in [10] for extensive discussion of this, and further discussion below and in electronic supplementary material S1 here).

The minimum convex hull volumes provide the minimum volume estimate for each animal, and a baseline for our sensitivity analyses in which we generated further models (figure 2; see also electronic supplementary material, movie S1). In our first model iteration, the minimal convex hulls were geometrically expanded by 21%, following a previous study on extant mammalian body proportions [9]. We subsequently generated a ‘maximal mass model’ in which the volume of the trunk segment was increased by 50%, and the volumes of all other segments by 100% [10]. From these three models, we produced two further models composed of the combination of segments that produced the most cranial and most caudal CoM positions (figure 2 and electronic supplementary material, movie S1). The ‘maximal’ volumetric expansions yielded an overall increase in body volume of around 60% in most of the sauropods modelled, which is well in excess of the upper 95% CIs (corresponding to a 32.2% expansion) found for mammals by Sellers *et al*. [9]. Indeed, our 100% expansions of head, neck, tail and all limb segments are more than three times greater than the upper 95% CI from Sellers *et al*. [9]. Our maximum caudal and cranial models are therefore composed of volumes that contain extremely large volumes at one end of the animal and minimum convex hulls that can unequivocally be considered to underestimate body segment volumes at the opposite end (figure 2 and electronic supplementary material, movie S1). Our decision to generate such large error bars through these extreme models reflects our cautious approach to volumetric reconstructions [9–14], the additional uncertainty associated with reconstructed dinosaur body volumes (e.g. different body shapes and sizes from living animals), and the goal to incorporate additional error margins to account for the more modest effects of skeletal articulation and incompleteness [10,12]. To place the magnitude of these error bars into relative context, we also calculated the CoM of two model iterations using the upper and lower 95% CIs convex hull expansion from [9]. Specifically, a caudal CoM model was derived by expanding caudal body segments (e.g. tail, hindlimbs) by the upper 95% CI expansion (32.2%) and cranial body segments (e.g. forelimbs, neck, head) by the lower 95% CI expansion (9.01%). Reversing these expansions yielded a cranial CoM model.

**Figure 3.**
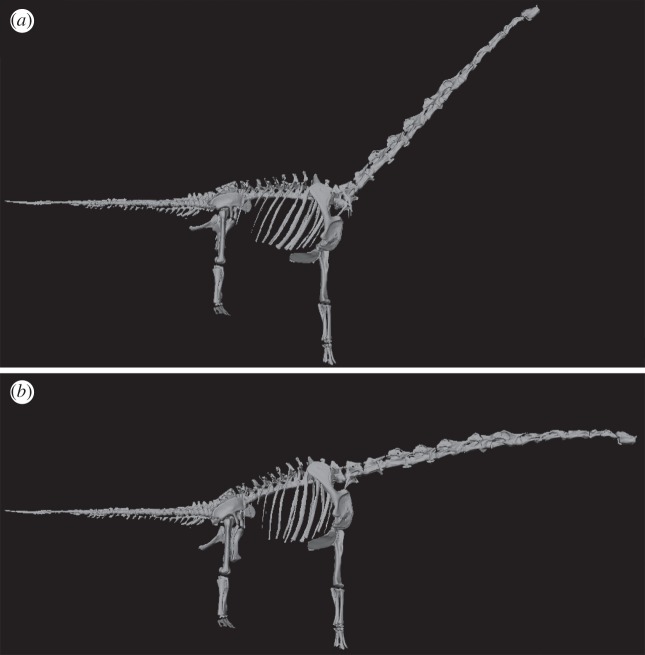
Examples of neck orientations used in the sensitivity analyses. *Giraffatitan* model in right lateral view with neck inclined to (*a*) 45° and (*b*) in the osteologically straight, undeflected state. In (*b*), the neck rises at a slope of between 18 and 27° above the horizontal (depending upon the reconstruction of the pectoral girdles upon the ribcage; fig. 4 in [17]). The pose in (*a*), on the other hand, corresponds to the familiar giraffe-like interpretation of macronarian neck posture, wherein the neck rises steeply either by reconstructing the vertebrae as if wedge-shaped at the base (as in the Berlin reconstruction) or by suggesting they habitually bent their necks to the limit of dorsiflexion at the base [19,20].

To further quantify likely error and evaluate the robustness of our conclusions regarding CoM and body segment evolution, we also carried out additional sensitivity tests. These sensitivity tests focus on the size of reconstructed zero-density respiratory volumes and errors associated with skeletal completeness in specific taxa, which is particularly key to our analysis of Cretaceous titanosauriforms. For example, neck length in *Dreadnoughtus* is poorly constrained by fossilized remains, whereas in *Sauroposeidon* and *Neuquensaurus* composite neck reconstructions have been produced from different specimens. We therefore ran additional analyses with the neck length of *Dreadnoughtus* altered by ±20% and those of *Sauroposeidon* and *Neuquensaurus* altered by ±10% to reflect this uncertainty. A similar approach was used to assess the sensitivity of CoM predictions to the size of zero-density respiratory structures, neck shape and tail length (see figures S6–12 in electronic supplementary material, S1). In addition, given the disparity in neck orientation reconstructions for sauropods in the literature and ongoing controversy regarding this important issue [17–21], we carried out a sensitivity analysis on neck posture (figure 3). Some derived sauropods (macronarians) have been suggested by some workers to have had more raised or inclined neck postures [19–20]. We therefore ran two sensitivity analyses related to neck orientation; one in which the neck segments of all macronarians were rotated dorsally by 45° (figure 3*a*), and a second one in which the neck of *Giraffatitan* was posed in the osteologically straight, undeflected state (figure 3*b*) [17]. Note that in the simple ‘necks inclined to 45 degrees' models all other body segments remained posed in the standardized postures used throughout this analysis (figure 3*a*). Applying this rotation to the models in the postures in actual mounted skeletons results in a much higher neck angle relative to the ground (e.g. around 68° to the horizontal in *Giraffatitan*). Exclusion of any curvature (e.g. S-shaped ‘swan-like’ curvature) also maximized the neck and head CoM displacements in these models (figure 3*a*). Thus, we are confident that our models cover the range of habitual neck postures postulated for sauropods to date [17–21].

In all model iterations, the masses of all segments were calculated using a density of 1000 kg m^−3^. However, zero-density respiratory structures in the head, neck and ‘trunk’ segments were reconstructed using surfaces lofted through NURBS circles that we shaped around the skeletal models (e.g. around the centra and ribs in the trunk segment), and we subtracted the volume of these structures from their overall segment volume before mass calculation, as in previous studies [10–14]. To account for the impact of skeletal pneumaticity on mass properties, we used convex hulling to enclose the volume of the centra of the cervical and dorsal vertebrae in each of the modelled sauropodomorph taxa, although this approach undoubtedly overestimates actual skeletal volume in these regions owing to interarticular spaces between bones. We then recalculated the mass of the neck and thoracic segments accordingly, giving the respiratory volume a density of 0 kg m^−3^ and the pneumatic bone volume a density of 900 kg m^−3^ (e.g. equating to an air space volume [22] of 50% if the density of air is 0 kg m^−3^ and the density of bone is assumed to be 1800 kg m^−3^). To the best of our knowledge, no study has explicitly quantified the impact of pneumaticity on the three-dimensional mass properties of a living archosaur, nor is there sufficient information in the literature at present to attribute differential levels of pneumatic air space volume within or between whole-body reconstructions of individual sauropod taxa. We therefore chose this simplified, standardized approach within our sauropodomorph models for our phylogenetic statistical analysis (see below). However, to provide the first insight into the potential nature and magnitude of differential pneumaticity on three-dimensional mass properties we also report the raw results from an additional sensitivity analysis in which we varied the density value attributed to the convex hull bone volumes in the thoracic and neck segments.

### 2.3. Phylogenetic and statistical analysis

We normalized estimated CoM positions and segment properties (segment lengths, masses and CoM positions) by division either by mean estimated whole-body mass (for masses) or by mean estimated whole-body mass^1/3^ (for linear parameters). We then used a simplified, high-level phylogeny of the sauropod branch of Archosauria (figure 1), with branch lengths based on first-occurrence data for fossils of each group, as the basis for estimation of ancestral node states for each parameter over the course of sauropod evolution (see electronic supplementary material, S1). As this approach often leads to branch lengths of zero, between first-occurrence taxa from the same geological formation, or owing to ghost-range issues, we substituted all zero branch lengths with lengths of one million years. Sensitivity tests surrounding this assumption (see figures S2 and S3 in electronic supplementary material, S1) did not qualitatively affect our conclusions.

The phylogeny and normalized data were then used as input to estimate ancestral node states with the ape package [23] for R (v. 3.02 (25 September 2013), http://cran.r-project.org/web/packages/ape/). Owing to better performance with variable (and long) branch lengths, the established method of ancestral state estimation (ACE) using maximum-likelihood and a simple Brownian evolutionary model were chosen over the older method of maximum-parsimony, or the less-established method of generalized least-squares. To test for phylogenetic signal in our parameters, we used the same simplified phylogeny and normalized data to generate Pagel's lambda scores (*λ*) with the phytools package [24] for R (http://cran.r-project.org/web/packages/phytools/). To assess the degree of correlation between our parameters, we first calculated phylogenetic independent contrasts (PICs) [25] from our raw (un-normalized) data and phylogeny, again using the ape package for R. PICs for parameters were then tested for correlation using Spearman's rho test (*ρ*, a non-parametric test was used due to non-normality in several parameters), performed using the Hmisc package for R (http://cran.r-project.org/web/packages/Hmisc/index.html). All signals and correlations were accepted as significant using an alpha level of 0.05. All raw and normalized mass property data are tabulated in electronic supplementary material S1, and our convex hull volumes and ACE outputs are freely available from http://dx.doi.org/10.5061/dryad.jq933.

## 3. Results

**Figure 4.**
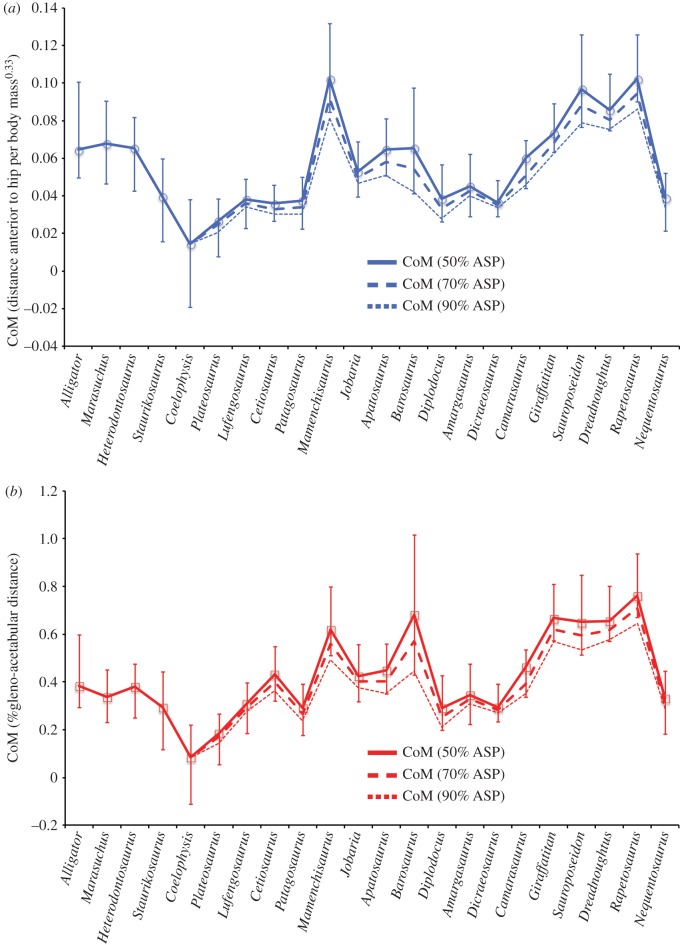
Raw CoM predictions for all taxa with normalization conducted using (*a*) distance cranial to the hip divided by body mass^0.33^ and (*b*) as a fraction of gleno-acetabular distance. Data plotted come from the Plus21% model iteration with densities in the neck and thoracic segments of sauropodomorph models varied to represent the effects of differential levels of pneumatic air space or ‘air space proportion’ (ASP, 50%, 70% and 90%) within the vertebral column in these regions. Error bars represent the CoM position of the maximum caudad and craniad models.

**Figure 5.**
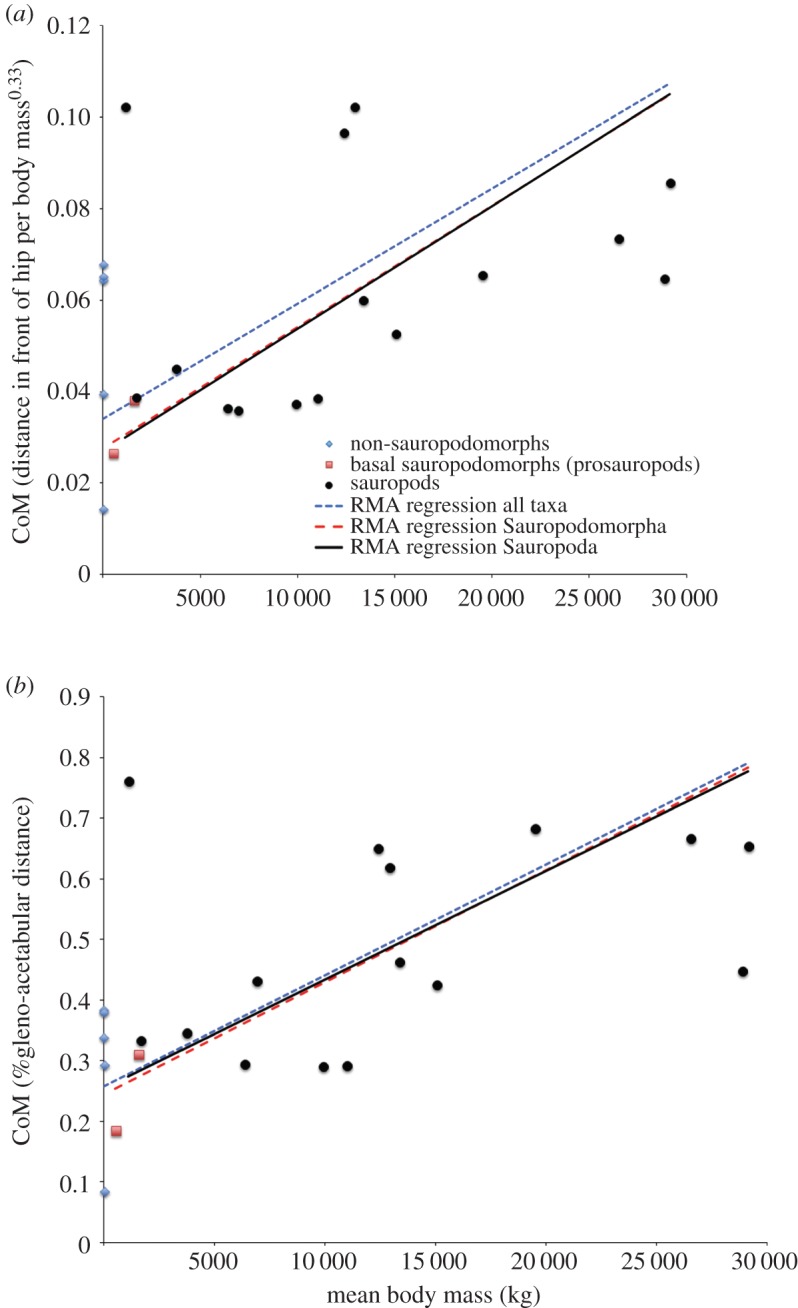
Reduced major axis regression of CoM against mean body mass using raw data for all taxa modelled in this study with CoM normalized by (*a*) distance in front of the hip divided by body mass^0.33^ and (*b*) as a fraction of gleno-acetabular distance. Regression statistics for (*a*) distance in front of the hip divided by body mass^0.33^ are: all taxa RMA regression slope = 2.52 × 10^−6^, intercept = 0.034, *r*^2^ = 0.157, *p* = 0.068; Sauropodomorpha RMA regression slope = 2.65 × 10^−6^, intercept = 0.276, *r*^2^ = 0.172, *p* = 0.098; Sauropoda RMA regression slope = 2.68 × 10^−6^, intercept = 0.027, *r*^2^ = 0.088, *p* = 0.282. Regression statistics for (*b*) as a fraction of gleno-acetabular distance are: all taxa RMA regression slope = 1.83 × 10^−5^, intercept = 0.258, *r*^2^ = 0.327, *p* = 0.005; Sauropodomorpha RMA regression slope = 1.85 × 10^−5^, intercept = 0.244, *r*^2^ = 0.243, *p* = 0.045; Sauropoda RMA regression slope = 1.80 × 10^−5^, intercept = 0.253, *r*^2^ = 0.138, *p* = 0.172.

Figure 4 shows the raw CoM predictions from the three model iterations (initial, max cranial and max caudal) for all taxa with normalization conducted using distance cranial to the hip divided by body mass^0.33^ (figure 4*a*) and as a fraction of gleno-acetabular distance (figure 4*b*). Raw CoM predictions with different degrees of skeletal pneumaticity in the neck and thoracic body segments are also shown (figure 4). Figure 5 shows reduced major axis (RMA) regression of raw CoM data against body mass for three taxonomic groups (all taxa, sauropodomorphs only, and sauropods only), again normalized by (figure 5*a*) distance cranial to the hip divided by body mass^0.33^ and (figure 5*b*) as a fraction of gleno-acetabular distance. In both cases, we find a weak positive linear relationship between relative CoM positions and body mass (figure 5).

**Figure 6.**
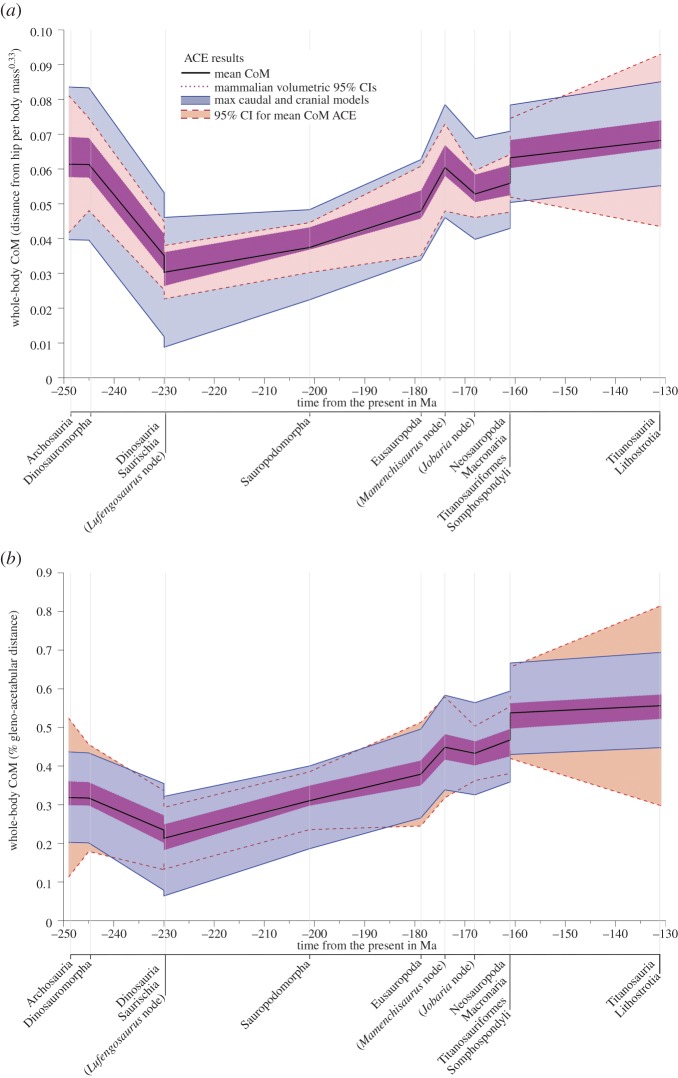
Estimated evolutionary patterns in whole-body CoM position along the craniocaudal axis of the body with data normalized by (*a*) distance in front of the hip divided by body mass^0.33^ and (*b*) as a fraction of gleno-acetabular distance.

Analysis of our ACE mean CoM data using Pagel's lambda (*λ*) suggests a significant phylogenetic signal (*λ* = 0.86) in CoM over sauropod evolution (figure 6). Qualitative assessment of our ACE for mean CoM over sauropod evolution suggests three trends (figure 6). First, in the Middle Triassic (approx. 245 to approx. 230 Ma), we find a caudad CoM shift from the ancestral position (approx. 0.3 gleno-acetabular lengths from the hip) in basal dinosauromorphs to a minimum of approx. 0.2 gleno-acetabular lengths from the hip in Saurischia (figure 6). This shift coincides with, and is plausibly associated with, the onset and progressive evolution of bipedalism in various dinosaur lineages. Second, we find a subsequent, steady craniad shift in the Late Triassic and Early–Middle Jurassic (approx. 230 Ma onwards), reaching approx. 0.45 gleno-acetabular lengths from the hip in Middle Jurassic sauropods (figure 6). This shift coincides with, and is plausibly associated with, the evolution of obligate quadrupedalism [15] and increased body size in the early sauropods. Third, we find a notable craniad shift in the Late Jurassic (approx. 161 Ma) reaching approx. 0.55 gleno-acetabular lengths from the hip in early Titanosauriformes (figure 6), represented by the brachiosaurid *Giraffatitan* (figure 1). This craniad mean CoM position is maintained within the brachiosaurid sister clade Somphospondyli (including the titanosaurian radiation), and thus in all titanosauriform lineages that survived into the Cretaceous (figures 1 and 6).

**Figure 7.**
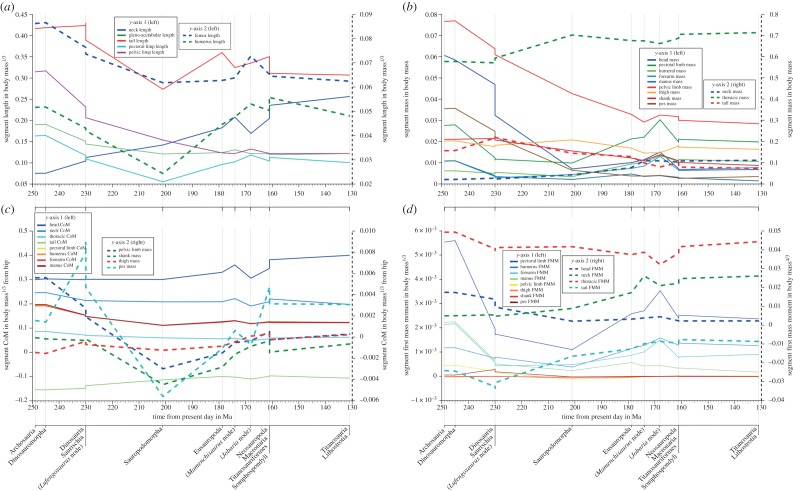
Estimated evolutionary patterns in individual body segment properties, expressed as (*a*) segment length normalized by body mass^0.33^, (*b*) segment mass as a proportion of body mass, (*c*) distance of segment CoM position from the hip normalized by body mass^0.33^ and (*d*) segment first mass moment normalized by body mass^1.33^.

Analysis of correlation in PICs using Spearman's rho (*ρ*) indicates that the strongest significant correlations were found between mean whole-body CoM position (figure 6) and the first mass moment (FMM, the product of segment mass and segment CoM) of the neck segment (*ρ* 0.98, figure 7*d*). Analysis of significant correlation in the components of neck FMM (figure 7*b,c*) suggests that changes in both neck CoM position (*ρ* 0.97) and neck mass (*ρ* 0.94) were similarly important to the effects of the neck on whole-body CoM position. Still significant but less strongly correlated was neck length (*ρ* 0.80), although this parameter cannot be fully separated from CoM position, barring considerable morphological change. Head CoM position (obviously strongly related to neck morphology) also shows a positive association with whole-body CoM (*ρ* 0.93; figure 7*c*).

The next strongest association with a cranially shifted whole-body CoM that was found was an increasing FMM of the thoracic segment (*ρ* 0.86; figure 7*d*). Analysis of FMM components suggests that changes in both segment mass (*ρ* 0.80; figure 7*b*) and segment CoM position (*ρ* 0.78; figure 7*c*) were similarly important to the effects of the thoracic segment on whole-body CoM position. Interestingly, only a weak to moderate negative association is evident between whole-body CoM and tail segment FMM (*ρ* −0.46; figure 7*d*). Of the FMM components, only the tail segment CoM shows a significant relationship (*ρ* −0.44; figure 7*d*).

Significant correlation was also found between whole-body CoM and our two measures of body size—estimated whole-body mass (*ρ* 0.83) and gleno-acetabular distance (*ρ* 0.65)—indicating that larger sauropods tend to have a more craniad whole-body CoM position, in agreement with the relatively weak trend seen in raw CoM data (figure 5). Weaker, but still notable, correlations were found between whole-body CoM and pectoral limb segment FMM (*ρ* 0.69; figure 7*d*), segment CoM position (*ρ* 0.77; figure 7*c*), and segment mass (*ρ* 0.77; figure 7*b*). Pectoral limb length showed a similar correlation (*ρ* 0.68; figure 7*a,b*). In the pelvic limb, significant correlations were weaker, and recovered only for mass (*ρ* 0.60; figure 7*b*) and length (*ρ* 0.49; figure 7). Additional discussion of patterns in individual body segment properties (figure 7) can be found in the electronic supplementary material.

**Figure 8.**
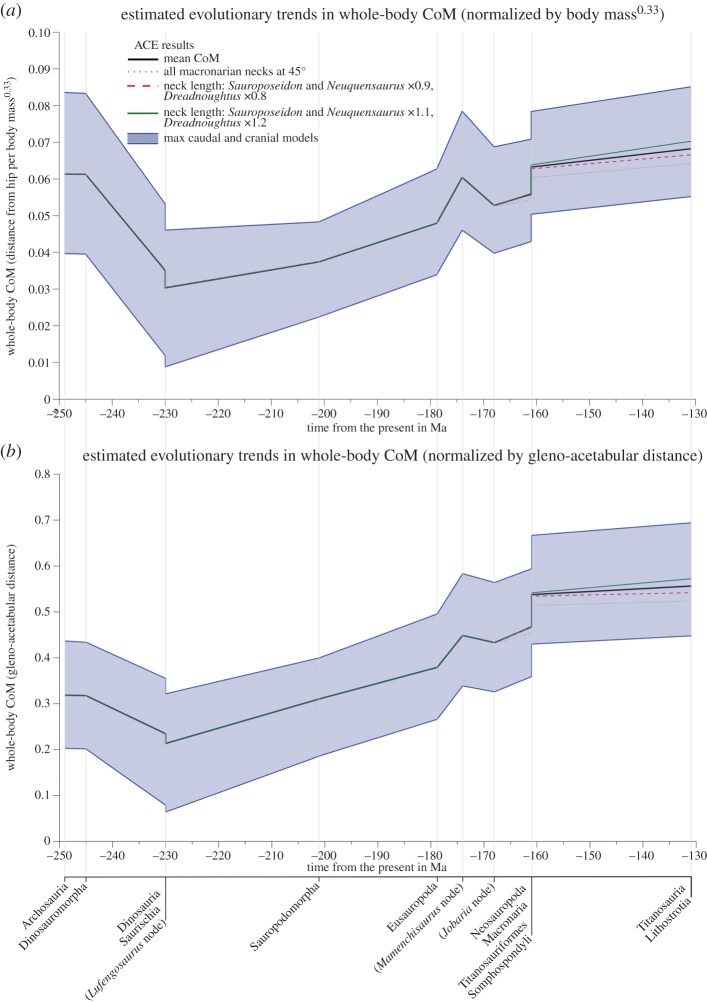
Comparison of our original estimated evolutionary patterns in whole-body CoM position (figure 6) to alternative reconstructions with inclined necks in macronarian taxa and increased/decreased neck lengths in *Sauroposeidon*, *Dreadnoughtus* and *Neuquensaurus*.

Extensive additional sensitivity analyses (see electronic supplementary material S1, figures S5–12) indicated that only neck orientation and high degrees of skeletal incompleteness in the neck (i.e. uncertain total neck length) have a notable impact on CoM evolution results (figure 8; see also electronic supplementary material, figures S5–12). Re-orienting all macronarian necks to highly inclined postures resulted in caudad and dorsad shifts in whole-body CoM (electronic supplementary material, figure S5) and moderately weakened the notable craniad shift in CoM seen in Late Jurassic titanosauriforms (figure 8). Changing neck length in *Sauroposeidon, Dreadnoughtus* and *Neuquensaurus* had a much smaller impact on CoM evolution (figure 8), with 10–20% shorter necks in these taxa only slightly weakened the sharp cranial shift in Late Jurassic titanosauriforms. Increasing neck length in these taxa exacerbated the aforementioned pattern (figure 8).

## 4. Discussion

### 4.1. Sensitivity analyses and uncertainties in centre-of-mass estimations

This analysis has a number of limitations that are largely inherent to studies of form and function in fossil vertebrates. Convex hulling generates volumetric reconstructions that are objectively based on the three-dimensional size and shape of fossilized skeletons. Thus, the patterns identified in our initial ‘mean’ model iteration (figures 4–6) are driven directly by similarities and differences in the three-dimensional size and shape of fossilized skeletons. However, the absence of soft tissue preservation means we must accept high levels of uncertainty in quantitative estimates of body size and shape (figures 2, 4 and 6). Indeed, this is confounded further in many instances by incomplete skeletal preservation, and herein we have employed a method that minimizes this effect as far as possible [10] (see also electronic supplementary material, S1) and additionally allows us to acknowledge and quantify associated errors through careful sensitivity tests (figure 8 and electronic supplementary material, figures S5–12), which is difficult if not impossible using more indirect, qualitative or subjective approaches [7].

Our maximum caudal and cranial model iterations represent highly implausible, if not untenable, body shape reconstructions and the model iterations constructed using the 95% CIs associated with average mammalian convex hull expansion [9] likely represent a more plausible approximation of volumetric error in our data (figures 4 and 6). If model iterations constructed using the 95% CIs associated with average mammalian convex hull expansion [9] are accepted as maximal error models then the three patterns in sauropodomorph CoM evolution noted above appear reasonably robust, particularly when normalized by gleno-acetabular distance (figure 6*b*). However, these current confidence intervals are based solely on mammalian taxa and clearly considerable data from living non-mammalian taxa are required to establish a more exhaustive and robust confidence intervals.

Our analysis provides the first quantitative insights into the potential nature and magnitude of differential levels of skeletal pneumaticity on CoM positions in archosaurs (figure 4). Wedel [22] attempted to provide some quantitative estimates of the potential magnitude of overall mass reduction in sauropods resulting from ‘empty’ air space in pneumatic vertebrae. Based on measurements from individual vertebrae from a variety of sauropod taxa, Wedel [22] suggested that air space proportion (ASP, the proportion of internal bone volume occupied by air) may have ranged between 0.32 and 0.89, and suggested ‘it seems reasonable to conclude that most sauropod vertebrae contained at least 50% air, by volume.’ As yet there has been no systematic study of how ASP varies within the body of an individual sauropod, or indeed, across taxa that would inform (quantitatively) on temporal and phylogenetic trends in ASP. We therefore mimicked the effect of differential ASP across our sauropod taxa by varying the density of our approximated cervical and thoracic vertebrae volumes across a range equivalent to 0.5–0.9 ASP (figure 4). This analysis demonstrates that increasing ASP in cervical and thoracic vertebrae yields more caudal CoM positions (as expected), and indeed that highly differential degrees of ASP across taxa could potentially alter relative CoM positions, thereby exacerbating or negating trends in CoM evolution seen here (figures 4 and 6).

Alternative reconstructions of sauropods with poorly preserved necks did not, by themselves, significantly impact ACE mean CoM predictions (figure 8). However, neck posture in macronarians (which does not exert an influence on our analysis of body proportions; figure 7) did have a much larger quantitative impact on CoM evolution, moderately weakening the notable craniad shift in Late Jurassic titanosauriforms (figure 8). However, again our alternative neck postures were deliberately inclined by extreme amounts, beyond existing quantitative estimates of habitual posture for individual taxa [17,18], and thus the data shown in figure 8 represent an extreme representation of the neck posture effects (figure 3) on CoM evolution.

Our sample of modelled taxa also represents only a small proportion of the total number of sauropodomorph species currently described. However, our sample does include at least one representative from each major sauropodomorph subclade, with the exception of Rebbachisauridae (as noted above). Rebbachisauridae is currently known only from the mid-Cretaceous and represents a basal clade of Diplodocoidea [1]. From within Rebbachisauridae, only *Nigersaurus* is potentially complete enough for volumetric reconstruction and body shape evaluation. Qualitative assessment of the skeleton of *Nigersaurus* suggests it would not have impacted significantly on our results. *Nigersaurus* has 13 cervical vertebrae that are not especially elongate [26] and so its neck is crudely similar to the short necks of dicraeosaurids (one fewer cervical) and *Jobaria* (the same number of cervical vertebrae and immediate outgroup to Neosauropoda in our study). Other titanosaurian subclades, not represented herein, have been named in the literature, but none of these preserve suitably complete skeletons, and most of these clades currently have limited support and comprise only a few putative taxa [27]. The 95% CIs for our ACE mean CoM data provide a measure of the uncertainty surrounding CoM predictions resulting from the inter-related effects of taxon sampling and branch lengths (figure 6). These suggest a notably higher degree of uncertainty surrounding ACE CoM estimations for Titanosauria and Lithostrotia, reflecting their relatively long branches lengths (figure 1 and electronic supplementary material, table S2; see electronic supplementary material, figures S2 and S3 for additional analysis).

As recognition of the high levels of uncertainty in our data (figures 2, 4, 6 and 8), resulting from factors inherent to studies of evolutionary form-function in fossil vertebrates, we restrict possible interpretations to large-scale trends in our data, which are supported by major changes in the three-dimensional proportions of fossilized skeletons (figure 7) noting the limitations we have highlighted where appropriate. We have made our volumetric reconstructions freely available, so that other workers can build on our analysis as new data become available, or so that alternative methods for reconstructing or modifying segments and body shapes as well as estimating phylogenetic patterns can be attempted.

### 4.2. Temporal and phylogenetic patterns based on mean mass property data

Our mean CoM data, and indeed, any single model iteration shown in figures 4–8 represent volumetric reconstructions in which the skeletal : body volume ratio is standardized across taxa. Thus, in these cases, patterns evident are driven directly by similarities and differences in the three-dimensional size and shape of fossilized skeletons. A highly elongate neck has been cited as ‘the most important key innovation’ in sauropod evolution [3]. Our new results reveal not only the evolutionary variation of relative neck size in sauropods, but also the central, but previously unquantified, role it played in the evolution of overall body shape and mass distribution, which we quantitatively represent for the first time using inertial properties (figures 6 and 7). Traditionally, neck elongation has been considered critically important because it potentially allowed more efficient food uptake by enabling a much larger feeding envelope, making food accessible that was out of the reach of other herbivores [3,17–21,28]. Given the apparent importance for feeding ecology, it is surprising (even given the relatively low sample size herein) that neither relative neck sizes (figure 7) nor whole-body CoM positions (figure 6) show any systematic correlation to skull functional morphology and inferred mechanics [29–33]. Recent morphometric and biomechanical analyses have supported the existence of two cranial morphofunctional types within Sauropoda: a ‘broad-crowned’ dental morphotype with robust skulls adapted to acquiring and processing relatively coarser fodder, and a ‘narrow-crowned’ dental morphology with reduced dentition and jaw adductor musculature that likely limited food choice [29]. At least some taxa displaying this latter morphotype have been hypothesized to rely heavily on branch stripping through specialized neck motions [29–30]. Our analyses show that both functional groups contain taxa with relatively long and short necks: the ‘narrow-crowned’ group includes titanosaurs and diplodocids with relatively long necks and other diplodocoids with relatively short necks, whereas the ‘broad-crowned’ group contains the extremely long-necked *Mamenchisaurus* and the shorter-necked *Camarasaurus* and *Jobaria*. Given our new findings, it is possible that both broad-crowned and narrow-crowned sauropods varied in neck length depending on other environmental and ecological parameters, such as the lushness of the habitat (e.g. a larger feeding envelope might be less necessary in environments where edible plants are plentiful) or the intensity of predation pressure. Alternatively, neck-driven changes in CoM may have interacted with feeding ecology in more complex ways. For example, it has been suggested that sauropods with more caudad CoM positions, such as diplodocids, were more capable of rearing bipedally to reach higher vegetation, while the more craniad CoM positions may have rendered other taxa incapable of such extended upright feeding [34]. Given we find that neck enlargement appears primarily responsible for the more craniad CoM positions in derived sauropods, it is possible that there was a shift away from feeding using a bipedal rearing strategy as neck elongation opened up increasingly larger feeding envelopes.

The temporal–phylogenetic patterns in relative CoM suggested in our analyses appear, however, to have stronger implications for locomotion. Specifically, more caudad CoM positions in basal dinosaurs are consistent with the mechanical demands of efficient and stable bipedalism [13], most obviously by enabling the vertical alignment of the centre of pressure and CoM while simultaneously maintaining a net extensor moment about the hind limb joints at mid-stance [35]. Our dataset supports the inference that Late Triassic bipedal basal sauropodomorphs might have evolved CoM positions ‘intermediate’ between the more caudad positions of basal bipedal dinosaurs and the more craniad loci of quadrupedal basal sauropods (figure 6), although the small magnitude of this difference relative to our error bars, and the mixed signals in our raw predictions for individual taxa (figure 4), mandate caution in this interpretation.

Increasing body size and the evolution of obligate quadrupedality in sauropodomorphs close to the sauropod radiation (figure 6) do not appear to be coincident with discrete or sharp shifts in the relative proportions of individual body segments (even segment lengths, which are not subject to the same error margins as mass properties). Rather, changes in segment proportions reflect the gradual craniad trend in overall CoM that started in basal sauropodomorphs, with continued increases in the length and masses of the neck and pectoral limbs, and relative decreases in the pelvic limbs and head (figure 7). Interestingly, although relative tail masses decreased slightly, relative tail length continued to increase in basal sauropods (figure 7), with extreme elongation in diplodocids [1], which probably accounts for the absence of a notable craniad shift in overall CoM in association with increased body size and quadrupedality in this lineage (figures 4 and 6; see also figure S4 in electronic supplementary material, S1).

The most striking link to locomotor evolution is the marked craniad shift in CoM in titanosauriform sauropods during the Late Jurassic (*ca* 160 Ma). The magnitude of this cranial shift is such that highly disparate skeletal : body volume ratios would be required to eliminate it completely (figures 6 and 8), although clearly moderate disparity in skeletal : body volume ratios could dilute this apparently sudden shift such that it falls more in line as a continuation of the gradual craniad trend in CoM positions seen throughout the Jurassic (figure 6). These cranial CoM positions, underpinned by increased neck size and maintained into the Cretaceous, are the most extreme positions in Sauropodomorpha (figure 6), and appear to be temporally coincident with the widespread appearance of ‘wide-gauge’ sauropod trackways in the fossil record [36–38]. The Jurassic sauropod footprint record is dominated by ‘narrow-gauge’ trackways in which opposing prints are beneath the body, close to the body midline. In contrast, ‘wide-gauge’ trackways, in which opposing prints are placed well lateral of the midline, dominate the Cretaceous trackway record, seemingly reflecting the emergence and diversification of Titanosauriformes [36–38].

Wilson & Carrano [37] proposed that titanosaurs (or a slightly more inclusive grouping of titanosauriforms [36]) possessed anatomical specializations in their limb girdles and long bones, as well as an overall wider body that led to the wide-gauge locomotion recorded in fossil trackways. It is interesting that the predominantly neck-driven craniad shift we have identified in Titanosauriformes is not concurrent with significant shifts in the relative mass or gross dimensions of limb segments (figure 7). Our data indicate that pectoral limb lengths increased in Early–Middle Jurassic Eusauropoda, and pelvic limb lengths continued to shorten until slightly later Eusauropoda (*Mamenchisaurus* node, approx. 174 Ma). However, subsequently, pelvic and pectoral limbs stabilized at similar relative lengths (approx. 0.1 body masses^1/3^) prior to the sharp craniad shift in the Late Jurassic. Broadly, similar patterns are evident for limb masses. Pectoral limb masses increased to a peak of approximately 0.03 body mass in Middle Jurassic eusauropods (*Jobaria* node, approx. 169 Ma) before declining to approximately 0.02 body mass (similar to estimated overall pelvic limb mass) in Late Jurassic Titanosauriformes (approx. 162 Ma). Our newly identified neck-driven craniad shift in overall CoM (figures 6 and 7) pre-dates the anatomical specializations noted in titanosaur limb girdles and long bones [37], perhaps suggesting that these osteological changes, and wide-gauge locomotion in general, were responsive to neck elongation and craniad CoM migration. The observation that CoM position remained relatively stable after the evolution of modified limb girdles and long bones in titanosaurs provides further indirect support for this argument.

Tail reduction in dinosaurs has previously been associated with a reduction in the size of the caudofemoralis longus (CFL) muscle, which serves as the principal locomotor muscle in most non-avian Reptilia [13]. It is plausible that tail reduction in sauropods is indicative of the decreasing importance of the CFL during locomotion in animals with a more craniad CoM. Indeed, within sauropods, tail reduction is most extreme in derived titanosaurs [1], and based on qualitative osteological analysis previous workers have hypothesized a reduction in the size of the CFL during titanosaur evolution [39,40]. Furthermore, these taxa also show a number of instances of enlarged or even novel muscle attachments on the pectoral girdle and forelimb in comparison to other sauropods [2,41,42]. The significant craniad shift in CoM in Titanosauriformes revealed by our new whole-body analysis provides a link between these anatomical patterns and suggests a systematic shift in locomotor anatomy (see discussion below), with derived Titanosauriformes possessing a more craniad CoM and enlarged forelimb musculature [2,41,42], but reduced tail-based hindlimb retractors [39,40]. Larger forelimb musculature would be expected in animals with a more cranially positioned CoM (reflecting more weight borne on the forelimbs), and would be beneficial in terms of countering reduced effective mechanical advantage of the limbs (mediolaterally) in the more sprawled postures suggested by wide-gauge trackways.

In contrast to these hypotheses, Henderson [5] proposed that size-correlated changes in body shape, ‘independent of clade’ (i.e. phylogeny), might have instead been responsible for differences in trackway gauge within sauropods. Specifically, Henderson [5] suggested that the body's CoM shifted forward as body size increased, and subsequently, this more cranially positioned CoM favoured wide-gauge locomotion. Although we find a relatively strong correlation between whole-body mass and CoM in our phylogenetic patterns (figures 6 and 7), our raw dataset shows a weak positive correlation between body size and CoM position within sauropodomorphs, with considerable scatter about the best-fit lines (figure 5). Overall, our larger and more phylogenetically and temporally widespread dataset exhibits a much stronger phylogenetic--temporal signal for CoM disparity in sauropodomorphs (figures 6 and 7), rather than purely size-driven trends, which is consistent with the pattern of locomotor evolution recorded by trackway gauge width [36–38].

Quantitative palaeoecological analysis indicates that titanosauriform body fossils and wide-gauge trackways are found primarily in inland paleaoenvironments, whereas non-titanosaurs and narrow-gauge trackways are often recovered in coastal palaeoenvironments [36]. It is tempting to speculate that our support for a concomitant craniad shift in CoM might provide the morphological mechanism for an evolutionary change in locomotion (narrow-gauge to wide-gauge), which is in turn connected to shifts in habitat preferences that facilitated the radiation of titanosaurs during the Cretaceous, whereas all other sauropod lineages dwindled and ultimately went extinct by the early Late Cretaceous (figure 1). However, such a scenario remains highly speculative, particularly in the absence of a clear mechanistic link between CoM and quadrupedal gait.

Modifying weight distribution in autonomous quadrupedal robots has been shown to systematically alter gait patterns, with weighted forelimbs producing lateral sequence gaits and weighted hindlimbs generating diagonal sequence gaits [43]. This link between mass distribution and gait has yet to be investigated in living quadrupedal animals, and these results may not have direct relevance for sauropods given that the CoM shifts (figure 6) appear to be driven predominantly by changes in the axial body segments, rather than the limbs (figure 7), although it is possible that titanosaurs may have evolved more muscular pectoral girdles [2,41,42] and reduced hip extensor musculature [39,40]. Clearly, more data on how CoM interacts with locomotor biomechanics in living quadrupedal animals are needed to better inform studies of extinct taxa. However, the uniqueness of the sauropod body plan in general, and the predominant role of their characteristically elongate neck in driving the evolutionary history of their body plan (figures 6 and 7), limit the extent to which extant taxa can serve as direct analogues for sauropod dinosaurs. This means that more direct modelling approaches, supported by basic principles established in extant animals, are likely to be key to addressing these and other controversies to further our understanding of the links between functional anatomy, ecology and macroevolutionary diversity in sauropodomorph dinosaurs.

## 5. Conclusion

Applying any consistent skeleton : body volume ratio to the sample of taxa modelled in this study yields patterns in body shape evolution that appear concurrent with major macroevolutionary and biomechanical events in sauropodomorph history (figure 6). A caudad shift in CoM in Middle Triassic Saurischia, associated with the evolution of bipedalism in various dinosaur lineages, was reversed in Late Triassic sauropodomorphs. A craniad CoM shift coincided with the evolution of quadrupedalism in the Late Triassic, followed by a more striking craniad shift in Late Jurassic–Cretaceous titanosauriforms, which included the largest sauropods (figure 6). These craniad CoM shifts are strongly correlated with neck enlargement (figure 7), which has long been considered the most important innovation in sauropod evolution and pivotal to their gigantism. However, all predictions are associated with a high degree of uncertainty resulting from incomplete skeletal remains, the absence of soft tissue preservation in fossils, and a relatively low sample size that results in long phylogenetic branch lengths (figure 6). Currently, uncertainty in the relative size of body segment volumes represents the most limiting factor in the robustness of CoM estimates, and clearly additional data from living archosaurs are required to better constrain confidence intervals in skeleton : body volume ratios applied to extinct taxa. Overall, this study highlights the difficulty of reconstructing the overall pattern of body shape evolution in sauropodomorphs, and by inference all fossil vertebrates, with high degree of confidence.

## Supplementary Material

ESM1: supplementary information on methods, and additional data.
