# Supplementary material for: Temporal and phylogenetic evolution of the sauropod dinosaur body plan
Source: R Soc Open Sci. 2016 Mar 30;3(3):150636. doi: 10.1098/rsos.150636 (PMC4821263; doi:10.1098/rsos.150636)
Supplement: ESM1: supplementary information on methods, and additional data. [file rsos150636supp1.docx]

Electronic Supplementary Material (ESM1) – additional methods, results and data

for

**Temporal and phylogenetic evolution of the sauropod dinosaur body plan**

Karl T. Bates, Philip D. Mannion, Peter L. Falkingham, Stephen L. Brusatte, John R. Hutchinson, Alejandro Otero, William I. Sellers, Corwin Sullivan, Kent A. Stevens & Vivian Allen.

^*^Corresponding author. E-mail: [k.t.bates@liverpool.ac.uk](mailto:k.t.bates@liverpool.ac.uk)

**Material and Methods**

***Sauropodomorph*** ***systematics:*** *Sauroposeidon* includes the material originally described as ‘*Paluxysaurus*’ [44], based on the synonymization proposed by D’Emic [45], although note that the ‘*Paluxysaurus*’ material was the basis for our model. Although originally regarded as a brachiosaurid [44], more recent analyses have demonstrated its position as a non-titanosaurian somphospondylan [45-47]. *Giraffatitan* follows the generic separation of *Brachiosaurus* into *B*. *altithorax* and *G*. *brancai* by Taylor [48]. We originally conducted our analysis twice, using two different tree topologies reflecting controversy in basal sauropodomorph relationships, with *Plateosaurus* and *Lufengosauru*s recovered as successively more nested sauropodomorph taxa, or as sister taxa, following trees derived from Yates [15] and Upchurch et al. [49], respectively. However, the nature of the differences between these two tree topologies meant that qualitative differences in CoM trends did not occur, and quantitative differences were minor (Fig. S1). We have therefore only followed the tree derived from Yates [15], with *Plateosaurus* and *Lufengosauru*s regarded as successively more nested sauropodomorph taxa in our analysis (Fig 1). Although *Cetiosaurus* and *Patagosaurus* have not consistently been recovered as a monophyletic Cetiosauridae [50], the largest and most recent phylogenetic analyses have recovered this grouping [2,51], including preliminary work on a detailed revision of *Patagosaurus* [52]. As such, we follow these papers in treating *Cetiosaurus* and *Patagosaurus* as sister taxa, and placed as eusauropods less derived than *Mamenchisaurus* in our tree topology.

We used a simplified, high-level phylogeny of the sauropod branch of Archosauria, with branch lengths based on first-occurrence data for fossils of each group, as the basis for estimation of ancestral node-states for each mass property parameter over the course of sauropod evolution (Fig. 1). The taxa, minimum ages and basis for node ages in our analysis are tabulated in Table S2. Our taxon sample resulted in a number of zero-length branches and in these cases we added 1 million years to node ages. Similar studies have used this approach (e.g. [4,13,53-56], adding between 1-5 million years to zero-length branches. However, the introduction of such forced constraints may potentially have an appreciable effect on some evolutionary analyses [56] and so to assess the sensitivity of our results to the substitution of zero-length branches with the value of 1 million years we carried out additional analyses. Firstly we re-ran our analysis of CoM evolution (Fig. 4) again with all branch lengths set to 1 million years (Fig. S2). All qualitative trends in CoM evolution were completely maintained when all branch lengths are standardized at 1 million years. However, quantitative differences are found. Specifically, the CoM positions for all nodes are displaced cranially relative to the original predictions, with nodes with larger true branch lengths experiencing greater craniad displacement (Fig. S2). Furthermore the 95% confidence interval about the mean ACE CoM prediction is both smaller and constant in magnitude relative to the original analysis (Fig. 6), such that the upper and lower intervals always lie within the range defined by the max caudal and cranial model iterations (Fig. S2). This indicates that the relatively long branch lengths for Titanosauria and Lithostrotia are responsible for the large 95% confidence intervals about the ACE mean CoM prediction in the original analysis (Fig. 6).

Secondly, we also re-ran our analysis 100 times systematically varying zero branch lengths in sequence with values between 0.01-1 (i.e. 10,000 to 1 million years). Plotting 95% confidence interval values for whole-body CoM at each alternative branch lengths value tested (0.01-1) shows that values plateau at approximately 0.3 million years suggesting that there little sensitivity to the value chosen for branch length between 0.3-1 million years (Fig. S3). Given these results and that other studies have either used 1 million years (e.g. [4,13,54-56]) or indeed advocated substituting zero branch lengths with higher values (e.g. 3-5 million years, [53]) in similar macroevolutionary studies of fossils, it seems justified to standardize zero branch lengths at 1 million years in this study.

**Additional Results**

***Additional whole-body CoM data:*** Figure S4 shows estimated evolutionary trends in whole-body CoM position along the craniocaudal axis of the body with all sauropod data included (i.e. including side-branches in the phylogeny). This plot therefore incorporates Cetiosauridae (node 7a), Diplodocoidea (node 10a), Diplodocidae (node 10b), Diplodocinae (node 10c; *Barosaurus*+*Diplodocus*) and Dicraeosauridae (node 10d), which are not shown in Figure 4 in the main text.

***Full description of the evolution of body segment mass properties:*** We found significant, strong phylogenetic signals in femur length (λ = 0.98), tail length (λ = 0.94), total pelvic limb length (λ = 0.91), and gleno-acetabular length (λ = 0.82), and weaker signals in total pectoral limb length (λ = 0.73), humeral length (λ = 0.68) and neck length (λ = 0.64) (Fig 7a, Tables S33-34). Qualitative assessment of patterns in ancestral state estimation (Fig 7a) indicates that, relative to body mass, overall limb lengths became shorter early along the lineage that led to sauropods, particularly within Late Triassic/Early Jurassic taxa (Fig 7a), and that while pectoral limb lengths increased again in Middle Jurassic Eusauropoda (~180 Ma), pelvic limb lengths continued to shorten until slightly later Eusauropoda (*Mamenchisaurus* node, ~174 Ma), after which pelvic and pectoral limbs stabilised at similar relative lengths (~0.1 body masses^1/3^). Estimated trends in relative humeral and femoral lengths were similar to those of their respective entire limbs (Fig 7a, Tables S33-34).

Ancestral state estimation of tail length and neck length indicates that they approximate inverse proportionality. After remaining constant at ~0.42 body mass^1/3^ in Middle Triassic Dinosauromorpha, tails became progressively shorter in Late Triassic–Early Jurassic Sauropodomorpha, reaching a minimum of ~0.27 body mass^1/3^ in Early Jurassic Sauropoda (~200Ma, Fig 7a). After a moderate lengthening in later Eusauropoda, tail lengths stabilised at ~0.3 body mass^1/3^ in Late Jurassic Titanosauriformes (~162 Ma, Fig 7a). Sauropod necks, however, got steadily longer from Triassic forms (~0.075 body mass^1/3^) onwards, with a dramatic increase of around 0.1 body mass^1/3^ between Middle Jurassic Eusauropoda (*Mamenchisaurus* node, ~174 Ma) and Late Jurassic Titanosauriformes (~162 Ma, Fig 7a). Interestingly, in Late Jurassic and Cretaceous Titanosauriformes, both neck and tail length stabilise at similar values (0.3 body mass^1/3^). Patterns in gleno-acetabular distance indicate a trend toward overall relatively shorter bodies over sauropod evolution, but with most change occurring earlier, dropping from ~0.2 body mass^1/3^ in Middle Triassic dinosauromorphs (~245 Ma) to stabilise at ~0.15 body mass^1/3^ in Late Triassic ~(230 Ma) and subsequent sauropodomorphs (Fig 7a).

Significant phylogenetic signals for relative segment masses were more prevalent but generally weaker than those for length (Fig 7b, Tables S33-34). Significant signals were found for tail mass (λ = 0.75), total pelvic mass (λ = 0.62), thigh mass (λ = 0.62), thoracic mass (λ = 0.57) and shank mass (λ = ~0.57). Equivocal signals were found for manus mass (λ = ~0.47), pes mass (λ = 0.45), total pectoral limb mass (λ = 0.43) and neck mass (λ = 0.43), with weak signals presented for humeral segment mass (λ = 0.42), head mass (λ = 0.37) and forearm segment mass (λ = 0.37).

Estimated evolutionary trends in relative tail mass (Fig 7b) indicate that, after a brief increase from ~0.16 body mass in Middle Triassic dinosauromorphs (~245 Ma) to ~0.22 body mass in Late Triassic sauropodomorphs (~230 Ma), relative tail mass decreased steadily to ~0.08 body mass in Late Jurassic Titanosauriformes (~162 Ma). In contrast, relative neck mass increased steadily from ~0.02 body mass in Late Triassic Sauropodomorpha (~230 Ma) to ~0.15 body mass (relatively more massive than the tail) in Late Jurassic–Cretaceous Titanosauriformes (~162 Ma onwards, Fig 7b). Relative thoracic mass shows fairly steep increases in early sauropod evolution (Fig 7b), increasing from ~0.55 body mass in basal dinosauromorphs and Late Triassic sauropodomorphs (~230 Ma) to ~0.7 body mass in Early Jurassic eusauropods (~180 Ma), remaining relatively stable in subsequent sauropod evolution. Head mass shows a strong decline over almost all of sauropod evolution, from ~0.06 body mass in Late Triassic dinosaurs (~230 Ma), to just ~0.008 body mass in Late Jurassic Titanosauriformes (~162 Ma).

Estimated evolutionary trends in relative pelvic limb mass show overall decreases throughout most of sauropod evolution, declining steadily from ~0.08 body mass in Middle Triassic dinosauromorphs (~245 Ma) to just ~0.02 body mass in Late Jurassic Titanosauriformes (~162 Ma, Fig 7b). Relative pelvic limb mass proportions also evolved: the mass of the pedal segment dropped rapidly from ~0.035 body mass in Middle Triassic dinosauromorphs (~245 Ma) to ~0.005 body mass in Early Jurassic eusauropods (~180 Ma). Estimates of femoral and crural mass are roughly proportionally equal for most of sauropod evolution, but both show moderate declines throughout, decreasing from ~0.02 body mass in Middle Triassic dinosauromorphs (~245 Ma) to ~0.01 in Late Jurassic and later Titanosauriformes (~162 Ma, Fig 7b).

Estimated overall pectoral limb mass shows a more complex pattern (Fig 7b). After an initial steep decline from ~0.028 body mass in Middle Triassic dinosauromorphs (~245 Ma) to ~0.01 body mass in Late Triassic sauropodomorphs (~230 Ma), pectoral limb mass reversed direction, back to a peak of ~0.03 body mass in Middle Jurassic eusauropods (*Jobaria* node, ~169 Ma) before declining to ~0.02 (similar to estimated overall pelvic limb mass) in Late Jurassic Titanosauriformes (~162 Ma). Trends in estimated pectoral limb segments were highly conservative (i.e. pectoral limb segment mass proportions remained relatively constant) until Middle Jurassic Eusauropoda (~179-174 Ma), when the mass of the humeral segment increased from ~0.008 body mass to ~0.01 body mass (Fig 7b).

Significant phylogenetic signals for segment CoM positions (relative to the hip) were generally strong (Fig 7c, Tables S33-34). These strong signals were found for relative CoM position of the humeral segment (λ = 0.89), the pectoral limb as a whole (λ = 0.89), the forearm segment (λ = 0.87), the manual segment (λ = 0.86), neck segment (λ = 0.82), thoracic segment (λ = 0.80), tail segment (λ = 0.77) and head segment (λ = 0.74). As expected, estimated trends in CoM position for all pectoral limb segments (and the limb as a whole) showed similar patterns (Fig 7c). From an initially more cranial position of ~0.2 body mass^1/3^ forward of the hip in Middle Triassic dinosauromorphs (~245 Ma), pectoral limb CoMs moved caudally to ~0.1 body mass^1/3^ in Late Triassic / Early Jurassic Sauropodomorpha (~202 Ma), after which their relative positions remained relatively stable. From an initial position ~0.25 body mass^1/3^ from the hip in Middle Triassic dinosauromorphs (~245 Ma), neck CoM shifted caudally to ~0.13 body mass^1/3^ from the hip in Late Triassic / Early Jurassic sauropodomorphs (*Lufengosaurus* node, ~202 Ma), where it remained until a cranial shift in Late Jurassic Titanosauriformes (~162 Ma) reverted neck CoM to ~0.25 body mass^1/3^ from the hip (Fig 7c). As expected, estimates of evolutionary shifts in head CoM position (i.e. the end of the neck) were very similar to estimated patterns seen in neck length (see above) - stasis (or very slight cranial shift) at ~0.3 body mass^1/3^ from the hip in Triassic dinosauromorphs and early sauropodomorphs, followed by a rapid cranial shift between Late Jurassic neosauropods (~164 Ma) and Titanosauriformes (~162 Ma, Fig 7c).

Estimates of CoM for the thoracic segment suggest that its mass distribution shifted caudally over sauropod evolution, from ~0.9 body mass^1/3^ in Middle Triassic dinosauromorphs (~245 Ma) to ~0.6 body mass^1/3^ in Late Jurassic Titanosauriformes (~162 Ma, Fig 7c). Tail CoM showed a cranial shift in mass distribution, shifting steadily from ~-0.15 body mass^1/3^ from the hip (note: negative values indicate a position caudal to the hip) in Middle Triassic dinosauromorphs (~245 Ma) to ~-0.1 body mass^1/3^ in Late Jurassic Titanosauriformes (~162 Ma, Fig 7c).

Significant phylogenetic signals for the first mass moment (FMM) of segments were found for the forelimb segments ((λ = 0.347-0.407), the head (λ = 0.394), neck (λ = 0.357), tail (λ = 0.536) and the thoracic (λ = 0.397) segments (Tables S33-34, Fig 7). Notable trends (Fig 7) include the progressive increase in FMM of the tail (note that tail FMM is negative, imposing an effective extensor moment about the hip, hence increases indicate a reduction in the FMM relative to the hip) during early sauropodomorph evolution, before stabilization at around ~164-169Ma at approximately -0.01 body mass^4/3^ (unnamed *Jobaria* node and basal neosauropods). Thoracic and particularly neck segment FMM closely parallel those of whole body CoM, particularly within sauropodomorphs (Fig 6). After reaching a minimum in basal bipedal dinosaurs, thoracic FMM remains relatively stable before a sharp increase at Somphospondyli in the Late Jurassic (~161Ma). Neck FMM parallels whole-body CoM even more closely, following the same trends as thoracic FMM except in showing a gradual increase throughout early sauropodomorph evolution, correlating with the gradual craniad shift in whole-body CoM that characterizes early Jurassic sauropod evolution (Fig 6).

**Additional sensitivity analyses**

In our previous computational volumetric studies of dinosaurs we have systematically and extensively quantified the impact of a number of poorly constrained parameters on mass and CoM predictions in dinosaurs [9-14, 57-58]. This has included the size and shape of body volumes and respiratory structures [9-14, 57-58], the impact of mounting posture such as inter-vertebral distance and ribcage ‘flare’ (e.g. [12,14]) and aspects of skeletal completeness and taphonomic distortion [10]. This previous body of research has consistently shown that the largest source of uncertainty (and therefore likely error) in dinosaur mass predictions derives from the size of reconstructed body volumes [9-14, 57-58]. It is for this reason that we expanded our minimum convex hull well beyond the upper 95% confidence interval suggested by our previous study of mammalian body volumes ([9] see Material and Methods in main text). Indeed, our 100% expansion of head, neck, tail and all limb segments are more than three times greater than the upper 95% confidence interval from Sellers et al. [9]. Our decision to expand significantly beyond previous 95% confidence intervals reflects our cautious approach to volumetric reconstructions [9-14, 57-58], the additional uncertainty associated with reconstructed dinosaur body volumes (e.g. different body shapes and sizes to living animals) and the goal to incorporate additional error margins to account for the more modest effects of skeletal articulation and incompleteness (e.g. [10,12,14]). However, to further quantify likely error and evaluate the robustness of our conclusions regarding CoM and body segment evolution in sauropods, we also present further sensitivity tests in this section. These sensitivity tests specifically focus on the size of reconstructed zero-density respiratory volumes and, in a subsequent section, errors associated with skeletal completeness in specific taxa modeled in this study (see below).

**Neck orientation sensitivity analysis**

In our analysis we employed a standardized reference posture in all models in which the neck and tail segments were fully extended horizontally from the main body segment (e.g. Fig 2, MovieS1). Standardizing posture is crucial for meaningful comparison of body segment proportions and their impact on whole-body CoM, and previous work has shown that large errors can potentially result from using non-standardised postures across models [14]. However, osteology hints that sauropod necks may have emerged as straight, collinear extensions of the anterior dorsal column [17-18,59]. In a neutral state of deflection, therefore, the base of the neck seems to have been inclined according to the slope of the back, i.e., sub-horizontal in diplodocids with their relatively short forelimbs, and increasingly sloped in those macronarians with relatively taller forelimbs [17-18]. The inclination of the back in taxa such as *Brachiosaurus* and *Giraffatitan* resulted in the extreme head elevation of 10 m [17] without the need to reconstruct the neck with wedged-shape vertebrae to form a giraffe-like curve, or to regard them as habitually dorsiflexed as a behavioral adaptation (e.g. [19-20]). However, given the disparity in neck reconstructions in the literature and ongoing controversy regarding this important issue [17-21,59], we carried out a sensitivity analysis on neck posture, with two specific reasons in mind. Firstly, to quantify from a methodological perspective, what impact our assumption of horizontal neck postures had on our results. Secondly, neck orientation might have been biologically and ecologically significant in sauropod dinosaurs, in particular potentially impacting upon feeding ecology. Under the assumption of a standardized horizontal neck posture, our results demonstrate that neck enlargement/elongation is a principal driver of the progressive cranial shift in CoM during sauropod evolution (Figs 6-7). Given that more derived sauropods (macronarians) have been suggested by some workers to have had raised or inclined neck postures (e.g. [19-20]), we sought to test if this phylogenetic and temporal cranial trend in CoM was significantly weakened if our models of macronarian taxa were re-posed with inclined neck segments (given that inclining the neck upwards would displace the neck and head segment masses caudally, thereby moving the overall CoM backwards relative to a model with a horizontal neck). We therefore ran two sensitivity analyses related to neck orientation; one in which the neck segments of all macronarians were rotated dorsally by 45 degrees (Fig. 3a), and a second one in which the neck of *Giraffatitan* was posed in the osteologically straight, undeflected state (Fig. 3b) [17]. Note that in the simple “necks inclined to 45 degrees” models all other body segments remained posed in the standardised postures used throughout this analysis. Applying this rotation to the models in the postures in actual mounted skeletons results in a much higher neck angle relative to the ground (e.g. around 68 degrees to the horizontal in *Giraffatitan*. Exclusion of any curvature (e.g. S-shaped ‘swan-like’ curvature) also maximized the neck and head CoM displacements in these models. Thus are we confident that our models cover the range of habitual neck postures postulated for sauropods to-date [17-21,59].

The results from this sensitivity analysis are shown in Figure S5 and Figure 8 in the main text. Increasing neck inclination results in caudad and dorsad shifts in the whole-body CoM in both scenarios tested (Fig S5a), with the magnitude of the displacement being greatest in taxa with relatively longer necks (Fig S5). The caudad displacement incurred in the ONP *Giraffatitan* model was extremely modest (Fig S5), being equivalent to just 2% gleno-acetabular distance. The displacements incurred from 45 degree neck inclinations were greater (Fig S5), hence we re-ran our phylogenetic CoM analysis with this data to see if it altered our original conclusions about CoM evolution in Sauropoda (Fig. 8). Interestingly the caudad CoM displacements incurred do not greatly impact phylogenetic analysis of CoM evolution (Fig 8); while there is weakening of the notable craniad shift in CoM seen in Late Jurassic Titanosauriformes this sharp craniad displacement is still present even under the extreme neck inclination scenarios explored here (Fig 8).

**Zero-density respiratory volumes**

In all our models, we attempted to produce similarly proportioned respiratory volumes in the thoracic and neck segments (e.g. the depth of zero-density thoracic respiratory volumes were always approximately half to two-thirds the depth of the ribcage; Fig. S6). Thus we attempted to produce zero-density volumes that were a similar proportion of the total accommodation space potentially available within thoracic segments, and similar proportion to the three-dimensional size of cervical vertebrate in neck segments. However, minor inconsistencies/human error in our approach, variation in skeletal completeness, and potentially real but unknown biological variation in respiratory anatomy within sauropods mean that uncertainty should be acknowledged and its effects explored quantitatively. A number of independent studies have concluded that the exact size chosen for zero-density respiratory volumes has a modest impact on overall mass and CoM predictions for dinosaurs [10-14,57-58,60]. These studies have largely focused on non-avian theropod dinosaurs where the whole body CoM is situated somewhat closer to the hip joint and therefore lies caudal to all zero-density respiratory volumes included in these reconstructions. In sauropods, the whole-body CoM lies more cranially and inside the largest “thoracic” zero-density volume included in our models and those of other workers (e.g. [5-6]). It would therefore be expected that the exact size chosen for zero-density structures would have relatively less impact on CoM predictions for sauropods due to volume/mass being subtracted more symmetrically about the whole-body CoM. To test if this is the case, and whether or not uncertainty and/or minor inconsistencies in the size of respiratory volumes impacts on our conclusions regarding CoM evolution (Fig. 6), we conducted a sensitivity analysis on impact of respiratory volume size on whole body CoM in our *Giraffatitan* model (Fig S6).

Figure S6a shows the original zero-density respiratory volumes in the thoracic and neck segments of our *Giraffatitan* model, along with two alternative reconstructions (Fig S6b-c). Our original thoracic respiratory structure had a volume of 5 m^3^ and our neck respiratory structure a volume of 0.332 m^3^. In our first alternative reconstruction (Fig. S6b), we reduced the dorso-ventral depth of the zero-density structures in both the thoracic and neck segments by approximately 33% and also reduced their medio-lateral dimensions by 5-30% at various points along their lengths. In our second alternative reconstruction (Fig. S6c), we expanded the thoracic zero-density structure to completely fill the ribcage and added approximately 33% to depth of neck zero-density structure and increased its medio-lateral dimensions by 5-30% at various points along its length. In our opinion, these alternative reconstructions are highly implausible, and indeed we are unaware of any living vertebrate with a ribcage entirely filled by a zero-density respiratory structure. The smaller structures (Fig. S6b) had volumes of 3.078 m^3^ (thoracic) and 0.231 m^3^ (neck), while the larger structures (Fig. S6c) had volumes of 7.363 m^3^ (thoracic) and 0.627 m^3^ (neck) respectively. The smaller alternative reconstructions (Fig. S6b) resulted in caudad displacements in the whole-body CoM of 0.026m in the Plus21% model, 0.047m in the most caudal model and 0.007m in the most cranial model. The larger alternative reconstructions (Fig. S6c) resulted in craniad displacements in the whole-body CoM of 0.064m in the Plus21% model, 0.096m in the most caudal model and a caudad displacement of 0.009m in the most cranial model. Thus even highly implausible zero-density volumes result in only minor displacement of whole-body CoM relative to the overall size of *Giraffatitan* (e.g. gleno-acetabular distance is ~3.17m, so a 0.064m displacement would be a ~2% relative shift) and indeed the displacements in normalized CoM are modest (Tables S39-40). Specifically, the maximum quantitative change in normalized CoM position equates to -3.17% (i.e. max caudal) to +2.67% (i.e. max cranial) when normalized by distance in front of the hip/body mass^0.33^ (Table S35), and -5.58% (i.e. max caudal) to +2.52% (i.e. max cranial) relative to the original CoM predictions when normalized by gleno-acetabular distance (Table S36). Given the relatively modest CoM displacements caused by these highly implausible alternative zero-density structures, and of course the generous maximal expansions of body volumes relative to the skeleton noted above, we feel it is justified to exclude a full sensitivity analysis of respiratory structures across our entire data set.

**Skeletal completeness tests**

***Overview:*** Although our convex hulling approach minimizes the impact of skeletal reconstruction by only utilizing the geometric extremes of body segments, it is important to recognize and test for the impact of specimen incompleteness on volumetric predictions [10]. Herein, we review skeletal completeness in our sample of modeled taxa before exploring its impact on our results in a number of different ways, following on from analyses presented in our previous study [10]. Firstly, we explore the issue of completeness on modeling results conceptually using simple geometric shapes to illustrate again that our convex hulling approach minimizes the impact of skeletal reconstruction by only utilizing the geometric extremes of body segments (Figs S8-9). Subsequently, we conduct a number of discrete case study experiments to quantify the potential margins of error resulting from skeletal incompleteness in sauropod dinosaurs (Figs S7, S10-S12).

Table S1 contains a brief description and quantitative scoring based on [61] of skeletal completeness in each of the sauropod taxa modeled in this study. The completeness metric provided is based on bone count for each genera [61] and provides only a very rough guide to the suitability of any given taxon for volumetric modeling. In general the percentage bone count will tend to underestimate the suitability of animal for volumetric reconstruction using convex hulling for a number of reasons, including most importantly:

1. Bone completeness metrics include both left and right sides of the animals. In volumetric models, digital scans of the one side of the animal (e.g. the left hind limb) can be digitally mirrored to recreate the other side (i.e. right hind limb) of the animal.
2. Certain parts of the skeleton are composed of numerous small bones (e.g. skull, manus and pes), which constitute an extremely small proportion of the animals volume, but a relatively high proportion of the skeletal completeness percentage.
3. Convex hulling minimize the need for geometric complexity, hence minimizes the need for the full percentage of bones to be present within any one ‘body segment.’

These concepts were explored in our previous reconstruction of *Dreadnoughtus* [10]. Lacovara et al. [62] state that “approximately 45.3% of the bones expected in a complete titanosaurian skeleton, and (depending on how bones are counted) up to 70.4% of the postcranial elements” are preserved in *Dreadnoughtus*. However, a significant proportion of the missing skeletal elements in *Dreadnoughtus* are those from within the skull, manus and pes (with the former two segments largely unrepresented in the preserved material). These segments make up a very small proportion of body volume (hence body mass) in sauropods (see Fig. 7), with the skull, for example, making up less than 1% total body mass/volume in all our volumetric models. In our previous reconstruction of *Dreadnoughtus* [10] we produced a volumetric reconstruction in which convex hulls were generated only around the preserved elements of the skeleton and compared this volume to that of the fully reconstructed convex hull body volume (Fig. S7). The volumetric reconstruction using only preserved skeletal parts equated to 88.5% of the fully reconstructed body volume (if the separation between preserved cervical vertebrae produced independently of our work by Lacovara et al. [62] is accepted as correct; this value is reduced to 83.2% if the neck is completely excluded and considered wholly incomplete), despite the fact that only 45% of the bones are preserved. All three factors listed above contribute to this discrepancy and explain why completeness by bone count underestimates the suitability of this animal for volumetric reconstruction [10].

With these factors in mind we review the skeletal completeness of each taxon individually below. In the discussion below only one of each paired appendicular element needs to be fully preserved for this bone to be “completely known”, with missing elements mirrored to complete the mount. To choose an extreme example, a skeleton preserving a skull and complete vertebral series, but only the left appendicular elements, would be considered complete for the purposes of its volumetric reconstruction.

***Plateosaurus:*** Our reconstruction of *Plateosaurus* is based on a complete and articulated skeleton of a single individual [63].

***Lufengosaurus:*** Our reconstruction of *Lufengosaurus* is based on the mounted specimen of a single individual known from an almost complete skeleton (IVPP V15) [64]. This comprises a full vertebral series, although some of the elements are incomplete, and near-complete appendicular skeleton, with all elements completely preserved on at least one side of the skeleton (PDM pers. obs. 2007).

***Cetiosaurus:*** Our reconstruction of *Cetiosaurus* is based on the referred specimen LCM G468.1968, a mounted partial skeleton of a single individual, described by Upchurch and Martin [65]. This specimen consists of nearly all of the presacral vertebrae (Cv2 onwards), the sacrum, 13 anterior–middle caudal vertebrae, ribs and chevrons, and a partial femur, although many of the individual vertebral elements are incomplete [58]. The remainder of the mounted specimen was modelled primarily from OUMNH specimens (including the lectotype) of *Cetiosaurus* [66], which preserve fragmentary dorsal and sacral elements, approximately 30 anterior–middle caudal vertebrae, scapulocoracoids, a sternal plate, humeri, ulnae, ilia, a pubis and ischium, femora, a tibia and fibula [67]. Elements unknown in *Cetiosaurus* were modelled from other sauropods [66].

***Patagosaurus:*** A cast based on PVL 4170 (mounted at MACN), representing a single individual, was used for our reconstruction of *Patagosaurus*. PVL 4170 preserves 8 cervical vertebrae (including an articulated series representing the cervical to dorsal vertebral transition), 10 dorsal vertebrae, the sacrum, the anterior portion of the tail, caudal vertebrae from the middle–posterior region of the tail, pectoral and pelvic girdles, a partial humerus, and femora [68]. Other parts of the mount were based on referred specimens of *Patagosaurus* or other sauropods.

***Mamenchisaurus:*** The mounted cast at IVVP was used in this study and is based on multiple specimens that together comprise a completely known skeleton [69].

***Jobaria:*** *Jobaria* is based on a composite mounted cast of several partial, anatomically overlapping specimens (MNN TIG) that essentially comprise a complete skeleton [70].

***Apatosaurus:*** CM 3018 is an essentially complete specimen of a single individual of *Apatosaurus*, with a near-complete vertebral column and all appendicular elements completely preserved on at least one side of the skeleton [71].

***Barosaurus:*** *Barosaurus* is based on AMNH 6341, which preserves the posterior half of the neck, the complete dorsal and sacral vertebral series, caudal vertebrae 1–29, and nearly all appendicular elements (excluding the manus) from at least one side of the skeleton.

***Diplodocus:*** Our reconstruction of *Diplodocus* is based on DMNS 1494, which preserves most of the vertebral column, as well as most appendicular elements (excluding the humerus, manus and pes) from at least one side of the skeleton ([72-73]; PDM pers. obs. 2008). Missing elements in the mount are based on other *Diplodocus* skeletons.

***Amargasaurus:*** *Amargasaurus* is known from a single individual (MACN N-15) preserving the skull and complete presacral vertebral series, a partial sacrum, a small number of caudal vertebrae from different regions of the tail, a scapulocoracoid, humerus, radius, ulna, partial ilium, femur, tibia, fibula, astragalus, and two metatarsals ([74]; PDM pers. obs. 2013).

***Dicraeosaurus:*** Our reconstruction of *Dicraeosaurus* is based on a composite mount of several skeletons (MfN MB.R specimens) that together comprise a near-complete skeleton. The majority of the mount is based on one individual (skeleton “m”) that preserves a partially articulated vertebral series from Cv3 to the 19th caudal vertebra, and pelvic and hindlimb elements (excluding the pes) from at least one side of the skeleton [75]. Most of the remaining elements are based on a second individual (skeleton “o”) that overlaps with the other skeleton.

***Camarasaurus:*** Our reconstruction of *Camarasaurus* is based primarily on CM 11338, an essentially complete skeleton of one individual [72], supplemented with information from AMNH 664.

***Giraffatitan:*** *Giraffatitan* is based on a composite mount (MfN MB.R specimens), primarily composed of the lectotype, a single individual preserving partial cervical and dorsal series, distal caudal vertebrae, pectoral girdle, forelimb, partial femur, tibia and fibula, and supplemented with the paralectotype (skull and anterior half of the neck), as well as material from additional skeletons. The right femur is based on a referred specimen, whereas the incomplete left femur is from the lectotype and has been reconstructed [48]. Despite the lack of anatomical overlap between specimens, the ratio of humerus to femur length is comparable to other brachiosaurids (e.g. *Brachiosaurus*).

***Sauroposeidon:*** The reconstruction of *Sauroposeidon* is based on referred material originally named *Paluxysaurus* [44]. This comprises remains of several individuals from one locality, representing most of the postcranial skeleton (as well as some cranial elements). The different individuals found at the locality appear to have been of approximately the same size, with only a small amount of size variation apparent in duplicated elements, limiting problems associated with scaling. Although serial counts are problematic and the most limiting factor of any reconstruction, seven middle cervical vertebrae were found in articulation [44], giving some indication of the elongation of the neck.

***Dreadnoughtus:*** *Dreadnoughtus* is known from partially articulated skeletons of two individuals that preserve two cervical vertebrae, most of the dorsal vertebrae, the sacrum, a nearly complete tail, pectoral and pelvic girdle, a humerus, radius and ulna, a femur, tibiae, fibulae, and some metatarsals [62]. As such, aside from the neck (see Sensitivity Analysis below), our reconstruction of *Dreadnoughtus* is based on an essentially complete postcranial skeleton.

***Rapetosaurus:*** *Rapetosaurus* is known from most of a skeleton of a young individual (FMNH PR 2209), with only parts of the tail missing [76].

***Neuquensaurus:*** Our reconstruction of *Neuquensaurus* is based on a composite mount of several individuals (MLP specimens), comprising most postcranial elements [40,77], and these include some associated vertebral series [78-79].

***Summary:*** Our analysis is therefore based on sauropodomorph genera known from near-complete skeletal remains, albeit not always from one individual animal. A number of taxa lack complete cervical and caudal vertebral series, which is problematic with dinosaur fossils in general, particularly with the most distal caudal vertebrae (e.g. [80]). *Dreadnoughtus* represents the most extreme case in this respect, preserving only two cervical vertebrae [62]. Overall neck length in *Dreadnoughtus* represents by far the most uncertain aspect of body size/shape in our analysis, and the initial reconstruction of Lacovara *et al.* [62] used in our model is based on that of *Futalognkosaurus*, a relatively closely related taxon, also from the Late Cretaceous of Argentina, which preserves the complete presacral axial column, as well as the sacrum and first caudal vertebra [81]. After addressing the issue of skeletal completeness and volumetrically modeling conceptually through use of geometric shapes, we subsequently investigate the specific impact of uncertain or unknown neck and tail segment lengths on our CoM predictions for sauropods.

***Convex hulling and geometric completeness***: In our previous study [10] we conducted an experiment using 3D geometric shapes to demonstrate that a minimum convex hull generated around an object will vary little - if at all - as a result of minor levels of incompleteness, as long as the overall size and shape of that object (as defined by its geometric extremes) are maintained. To demonstrate this we generated a series of convex hull volumes around basic geometric shapes (spheres and cylinders; Figs S8-9) that had been subjected to increasing levels of random decimation. In the case of a sphere, the calculated convex hull volume remained above 98% the original value even when 80% of polygons making up that sphere have been removed ([10], Figs S8b). In cylindrical shapes the impact of decimation/incompleteness is even less significant (Fig. S9): indeed, as long as the geometry of the circular ends of the cylinder were maintained there was absolutely no change in the volume of the convex hull volume ([10], Figs S9). Only when the ends of the cylinder were decimated simultaneously was volume change incurred (Fig. S9b). It follows from this that the CoM of the convex hull will experience only minor changes with increasing levels of shape incompleteness. This was indeed the case with both randomly decimated spheres (Fig. S8c) and cylinders (Fig. S9c), with maximum CoM errors less 2.5% when extremely high levels of decimation are applied to both shapes.

***Neck shape and length sensitivity tests:*** Here we conduct a series of additional sensitivity experiments to test directly how skeletal completeness in the neck impacts on overall CoM predictions in sauropods. First, we produced an alternative (hypothetical) reconstruction of the neck segment in *Giraffatitan* using only the skeletal components preserved in *Dreadnoughtus* (Fig. S10). In our previous study [10] we demonstrated that this ‘incomplete’ neck model yielded a 9% lower volume for the neck segment relative to the original convex hull model of *Giraffatitan*, which corresponds to a 0.9% lower overall body volume [10]. This resulted in whole-body CoM displacements of 0.007m, 0.006m and 0.009m in the Plus 21%, most caudal and most cranial models respectively. In terms of normalised CoM position these absolute displacements equate to a maximum error of 0.003 gleno-acetabular length (i.e. a 0.3% error when normalised by gleno-acetabular distance). This result is consistent with the results of geometric shape decimation (Fig. S8-9) which demonstrate that as long as the overall gross size and shape of the object/body segment are preserved, completeness will not impact on resulting minimum convex hull volume and subsequently on mass property predictions [10].

The above neck analysis *Giraffatitan* largely represents an analysis of shape effects on convex hull reconstructions of volume, and the results will also apply to other body segments, notably the tail, when approximate total length is known but not all component parts are preserved and/or perfectly reconstructed in the skeletal mounts. However, the above analysis does not account for uncertainties in total neck length, which as discussed above and in our previous work [10], is poorly constrained in *Dreadnoughtus*. Building again on our previous work [10] we therefore analysed four additional neck reconstructions in our *Dreadnoughtus* model, in which overall neck length was decreased by 10% and 20% and increased by 10% and 20% (Fig S11). These decreases/increases were achieved by increasing the spacing between each preserved vertebra by equal amounts, which will have a larger (i.e. worst case scenario) impact on convex volume than increasing neck length by simply displacing only the most cranial vertebrae [10]. Zero-density respiratory structures in the neck were also increased/decreased in length accordingly in each reconstruction. Decreasing and increasing neck length by 10% yielded a 9% change in neck volume relative to the original convex hull model [10], with 20% changes resulting an 18% change in volume. However, the impact on neck mass was slightly lower, as changing neck length also demands compensatory changes to the volume of the zero-density respiratory structure in the neck segment. The whole-body CoM predictions from the Plus 21% iterations of these models are compared to the original reconstructions in Fig. S13c-d. Changing neck length by 10% resulted in CoM displacement equivalent to 0.025, or 2.5%, gleno-acetabular length, and 20% changes approximately double that at around 0.05, or 5%, gleno-acetabular length (Fig. S11d). The displacement resulting from 20% changes in neck length equate to approximately 50% of our original maximum caudal and maximum cranial error bars based on zero and maximal expansion of the convex hulls (Fig. S11d). Although this assessment is entirely qualitative, we would acknowledge that this magnitude of CoM displacement consumes a moderate proportion of the redundancy we aimed to build into our error bars by expanding volumes by three times the magnitude of the 95% confidence interval found by Sellers et al. [9] for average mammalian convex hull expansions. It is worth noting that 20% changes to neck in *Dreadnoughtus* represents a considerable change relative the original estimation (shortening/lengthening by more than 2.1m) and, for example, that the whole-body CoM of the maximum cranial iteration of the model with a 20% increase in neck length lies extremely close to the glenoid at 0.91 (or 91%) gleno-acetabular length. A 30% increase in neck length would likely result in the whole-body CoM of the maximal cranial iteration lying in front of shoulder joint, within the neck segment itself, which is untenable. It is further worth emphasizing that *Dreadnoughtus* represents the worst-case scenario in terms of neck length uncertainty in our analysis. Other taxa (e.g. *Rapetosaurus*, see above) do lack the most proximal cervical vertebrae and so their true neck is not completely certain. However, uncertainty in volume in these instances only applies to this missing cranial portion of the neck (which is also the thinnest portion of the neck) and errors are likely to be relatively low and similar in magnitude to that demonstrated below in our analysis of tail lengths (see below).

To investigate how the described uncertainty in *Dreadnoughtus* neck length impacts on our analysis of CoM evolution within sauropodomorphs, we re-ran our evolutionary analysis of CoM with this additional neck length data to see if it altered our original conclusions about CoM evolution (Fig. 8). This analysis also included +/-10% changes to overall neck length in our models of *Sauroposeidon* and *Neuquensaurus,* as described in the main text. These taxa are both known from several individuals that allow partial, composite neck reconstructions. We therefore included them in our additional neck length to examine the sensitivity of the notable (and seemingly neck-driven) cranial shift in whole-body CoM observed in titanosauriforms in the Late Jurassic–Early Cretaceous (Fig. 6). The results of this analysis are presented in Figure 8 in the main text.

***Tail length sensitivity tests:*** Our original *Dreadnoughtus* model included a 10% extension in tail length relative to the preserved skeletal material to account for missing distal caudal vertebrae, based on the reconstruction of Lacovara et al. [62]. Figure S14 shows this initial reconstruction along with alternative tail reconstructions, with 5%, 20% and 30% preserved tail length added. These models demonstrate extremely modest sensitivity to absolute and normalized whole-body CoM position to uncertainties in tail length resulting from unpreserved distal caudal vertebrae. Absolute whole-body CoM displacements relative to the original model are 0.005m, 0.013m and 0.029m for the Plus 5%, Plus 20% and Plus 30% reconstructions shown in Figure S12. These displacements equate to normalized shifts of just 0.001, 0.004 and 0.007 gleno-acetabular lengths respectively in animal with a total body length of >25m [62].

**Supplementary Figures**

**
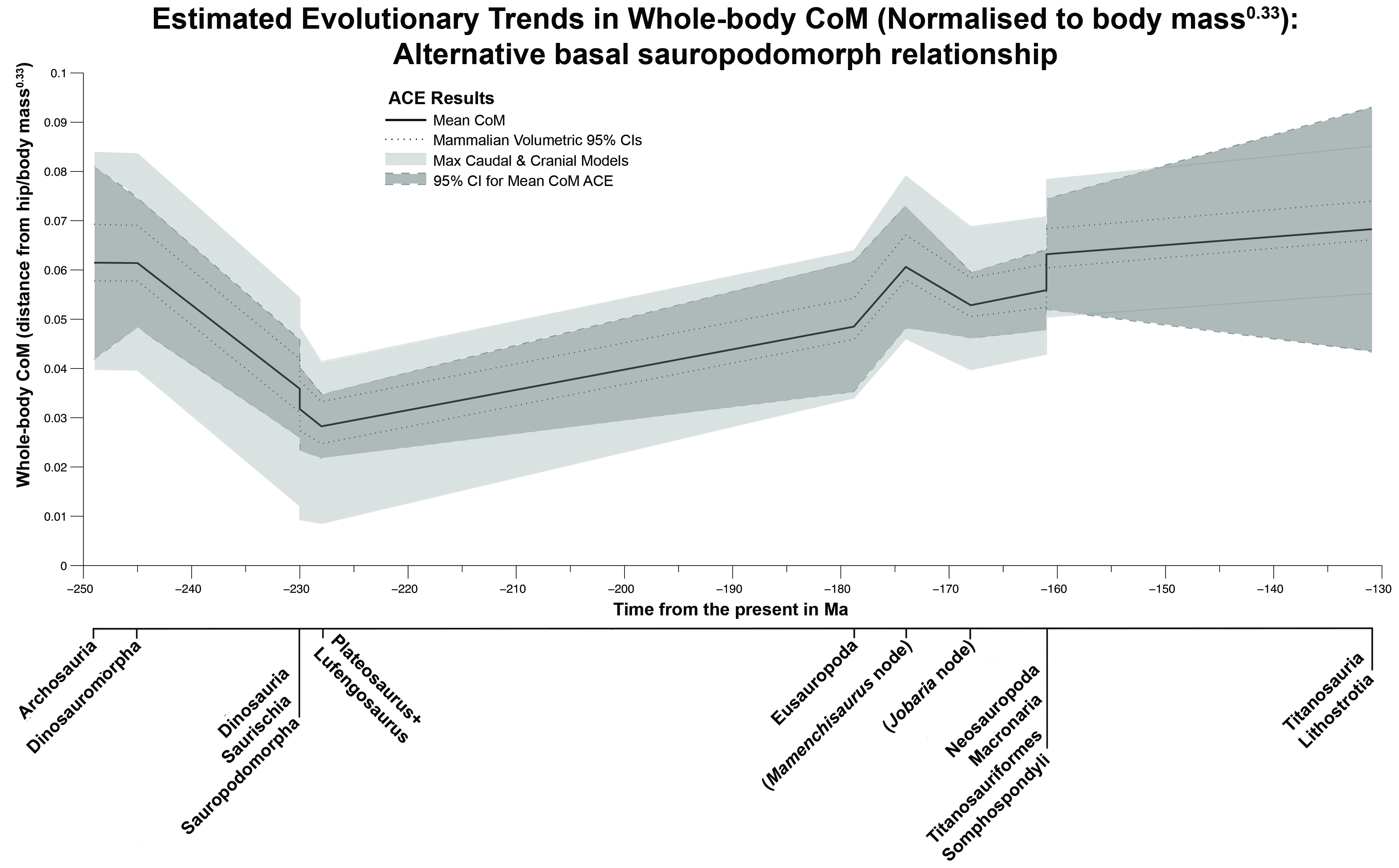
**

**Figure S1.** Estimated evolutionary trends in whole-body CoM position along the craniocaudal axis of the body with data normalized by distance in front of the hip divided by body mass^0.33^ using the alternative phylogeny of Upchurch et al. [49] in which *Plateosaurus* and *Lufengosauru*s are recovered as sister taxa.

**
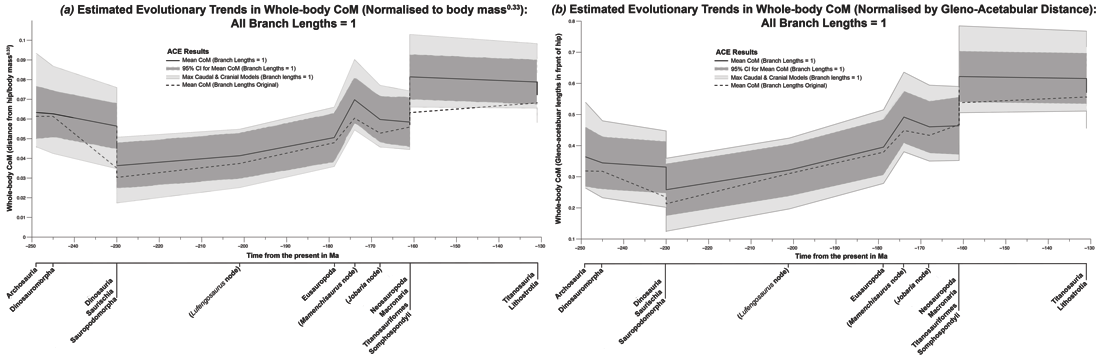
**

**Figure S2.** Estimated evolutionary trends in whole-body CoM position along the craniocaudal axis of the body with data normalized by ***(a)*** distance in front of the hip divided by body mass^0.33^ and ***(b)*** as a fraction of gleno-acetabular distance with all branch lengths standardized at 1 million years.

**
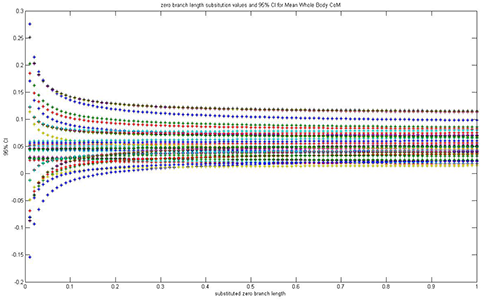
**

**Figure S3.** 95% confidence interval values for whole-body CoM in our analysis for the range of alternative branch lengths values (0.01-1) tested. Values plateau at approximately 0.3 million years suggesting that there little sensitivity to the value chosen for branch length between 0.3-1 million years.

**
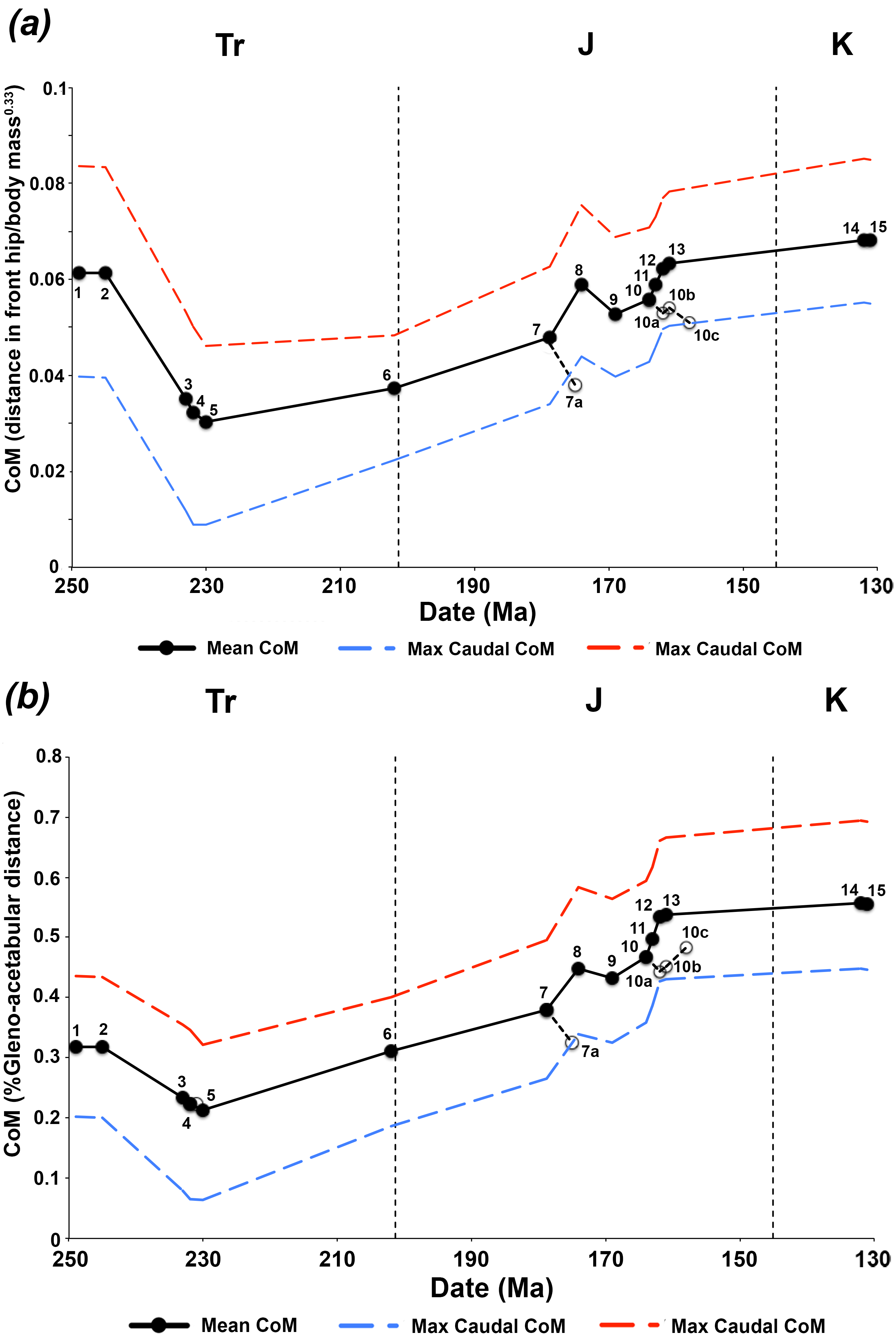
**

**Figure S4.** Estimated evolutionary trends in whole-body CoM position along the craniocaudal axis of the body with data normalized as *(a)* distance cranial to the hip divided by body mass^0.33^ and *(b)* a fraction of gleno-acetabular distance, with all sauropod data included (i.e. including side-branches in the phylogeny not shown in Figs 6, 8). Black circles and solid black line depicts the trend along the main evolutionary sequence using the ‘Plus21%” model (side branches/nodes represented by dashed black line and open circles), with red and blue lines representing the most caudal and cranial CoM models (see Fig 2) at main sequence nodes. Node numbers correspond to those in Fig 1 and Tables 2-3 and 33-37 (for reference, node 1 is Archosauria, node 3 is Dinosauria, node 6 is Sauropodomorpha and node 8 is Eusauropoda, node 7a is Cetiosauridae, node 10a is Diplodocoidea, node 10b is Diplodocidae, node 10c is Diplodocinae (*Barosaurus*/*Diplodocus*) and node 10d is Dicraeosauridae).

**
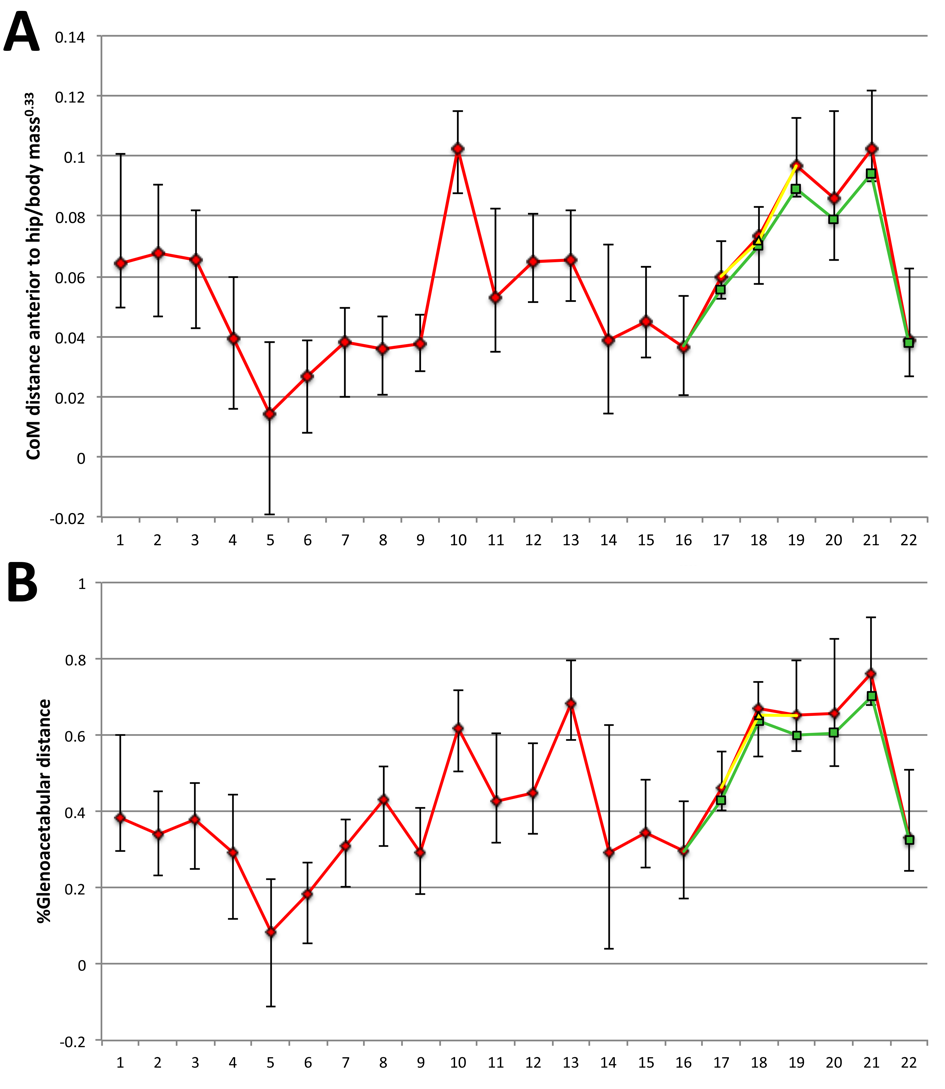
**

**Figure S5.** Results of neck orientation sensitivity analyses. Raw CoM predictions for all taxa with normalization conducted using *(a)* distance cranial to the hip divided by body mass^0.33^ and *(b)* as a fraction of gleno-acetabular distance with sensitivity analysis of neck orientation in the macronarian taxa shown. The green line/data points show results with all macronarian necks inclined to 45 degrees, and the yellow line/data point shows the original raw data but with the neck of *Giraffatitan* articulated in the ONP of Stevens [17]. Neck inclination results in caudad shifts in CoM relative to the original models with horizontal necks, with the magnitude of displacement strongly correlated to overall neck length.


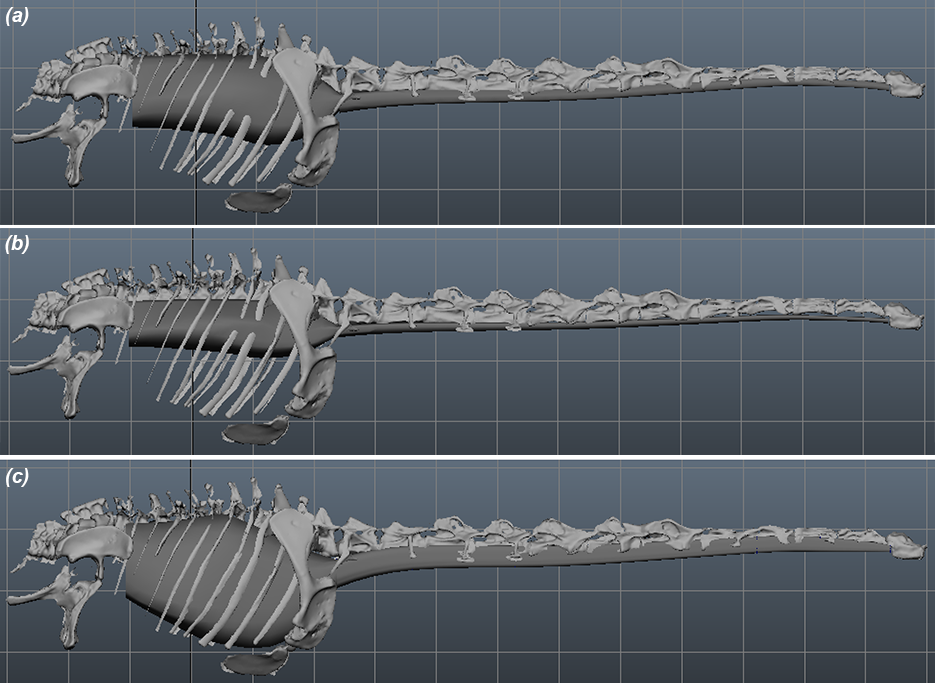


**Figure S6.** Sensitivity analysis on zero-density respiratory volumes in our *Giraffatitan* model. *(a)* Original zero-density volumes in the thoracic and neck segments are shown above the *(b)* reduced and *(c)* greatly enlarged alternative reconstructions.


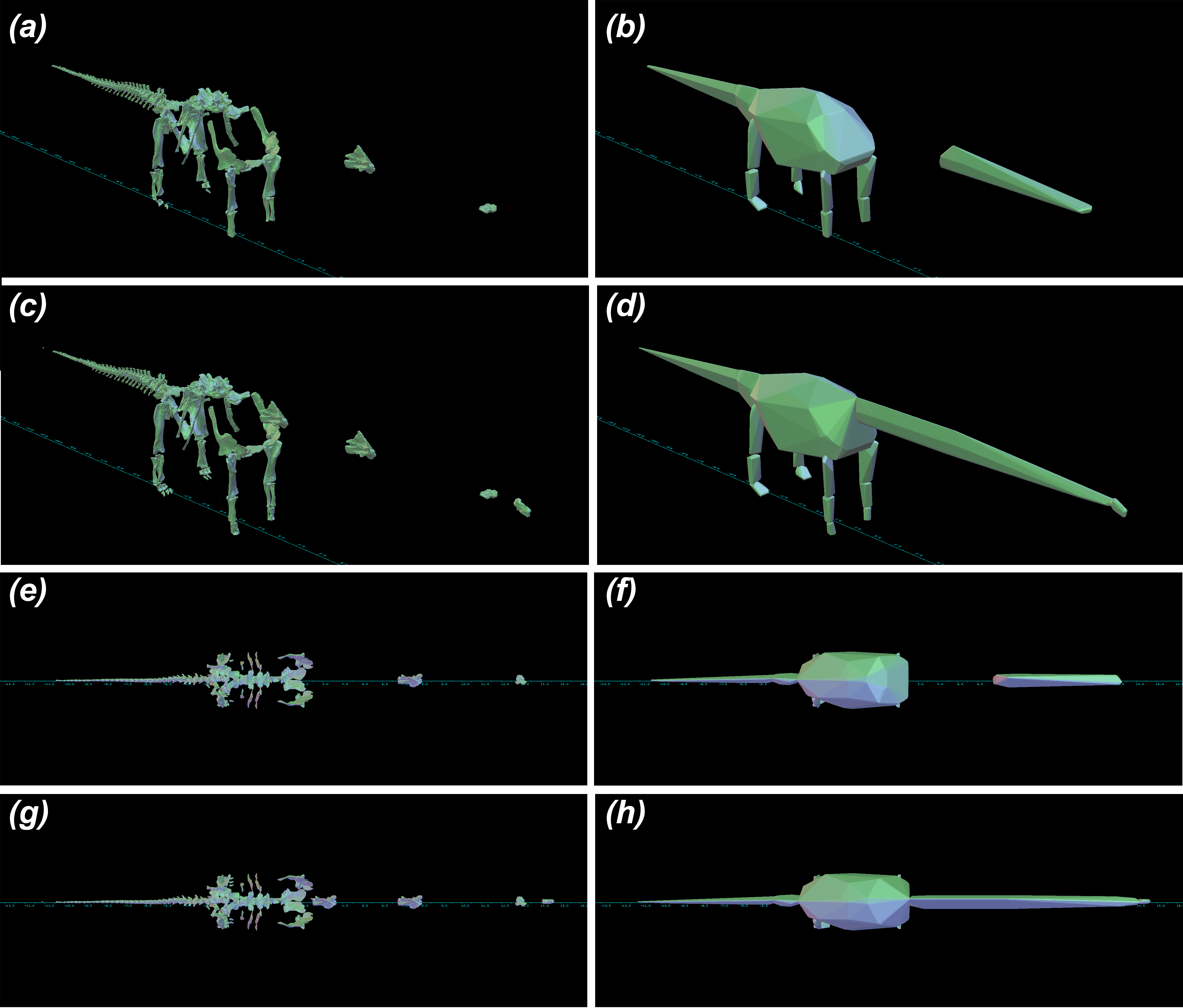


**Figure S7.** Skeletal and volumetric reconstruction of *Dreadnoughtus*, with explicit depiction of preserved and reconstructed skeletal components and their impact on the whole body convex hull reconstruction. ***(a & e)*** The preserved skeletal components and ***(b & f)*** convex hulls in generated around only those preserved components (in oblique and dorsal views), yielding a total volume of 23.83m^3^, which equates to 88.5% of the volume of full body convex hull reconstruction, shown in ***(d)*** and ***(h)***. Figure reproduced from [10].


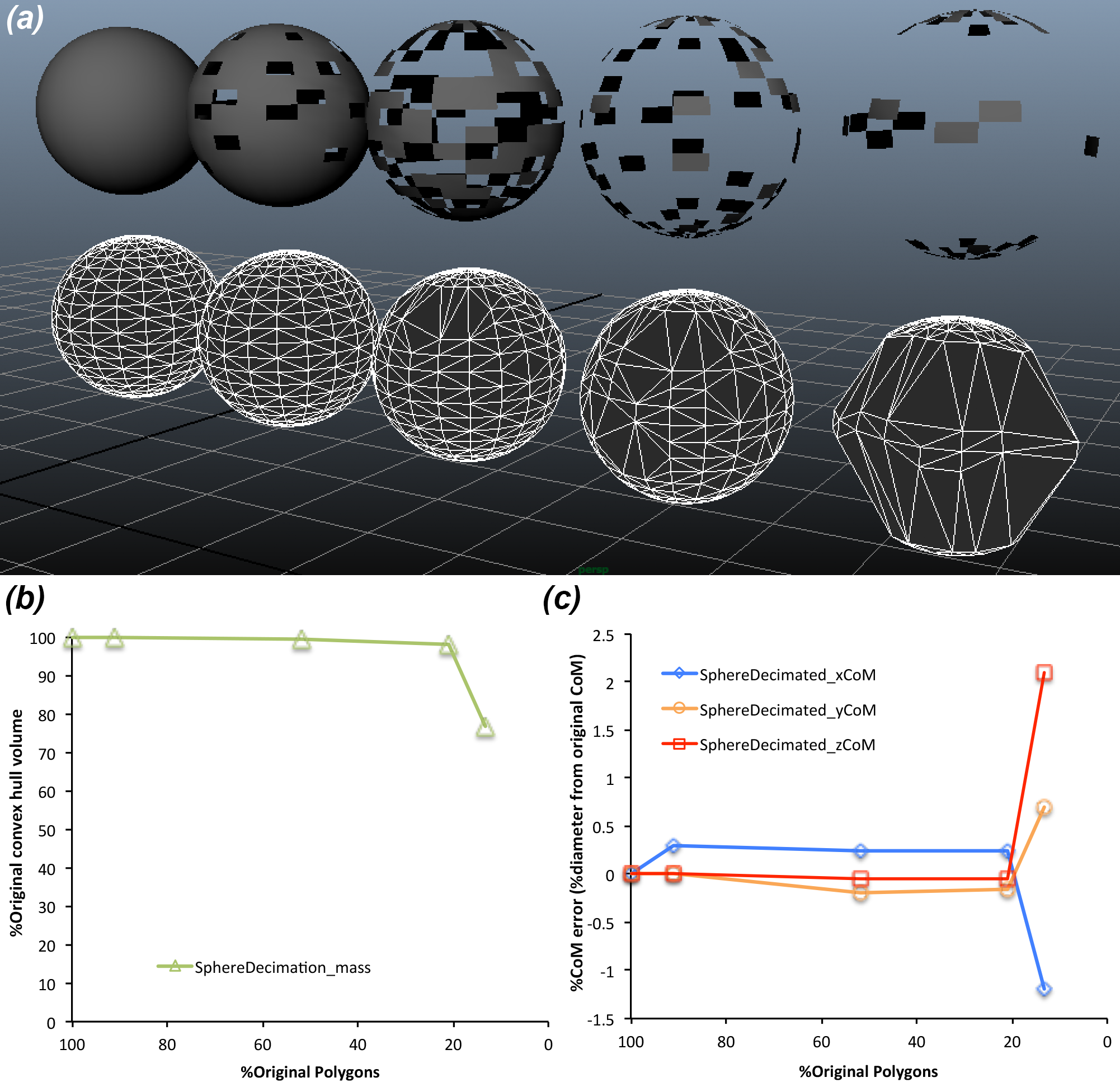


**Figure S8.** *(a)* Demonstration of the effect of object completeness on minimum convex hull volume reconstruction, using a series of increasingly randomly decimated spheres. (b) In our previous study we demonstrated that the impact on the volume/mass of the reconstructed convex hull was minimal up to levels of approximately 80% decimation [10], but here *(c)* we also show that the same is true for the CoM of reconstructed convex hulls, with errors of less than 2.5% on any given co-ordinate axis with more than 80% polygon decimation.


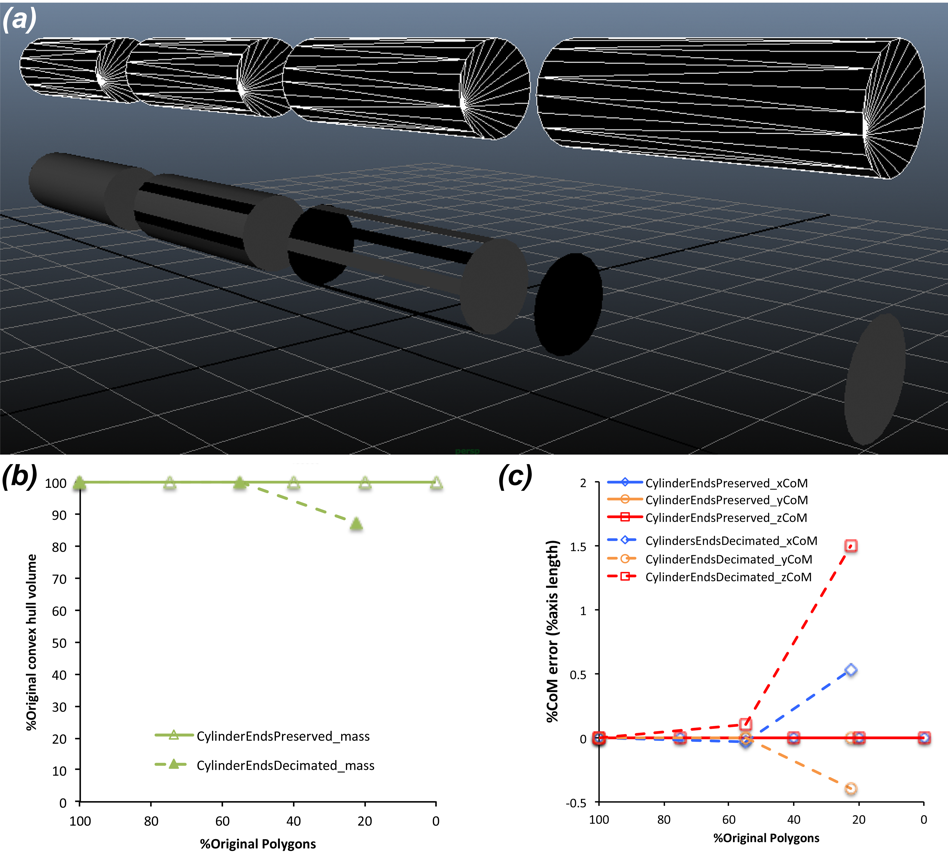


**Figure S9.** *(a)* Demonstration of the effect of object completeness on minimum convex hull volume reconstruction, using a series of increasingly randomly decimated cylinders. *(b)* In our previous study we demonstrated that there was absolutely no impact on the volume/mass of the reconstructed convex hull when the ends of the cylinders were preserved but the remainder of the cylinder removed. *(c)* As a result there is also absolutely no impact on CoM under these circumstances. When the ends of the cylinder are simultaneously decimated then error is incurred, but errors in CoM are less than 2% on any given co-ordinate axis with approximately 80% polygon decimation.


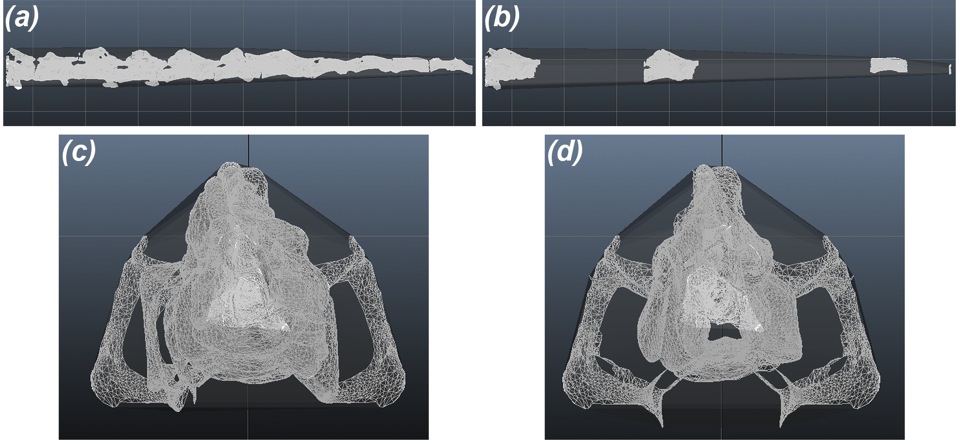


**Fig. S10.** Comparison of the minimum convex hull volumes generated for the neck segment in *Giraffatitan* when the *(a & c)* full skeleton is used versus a model *(b & d)* in which only the elements preserved in *Dreadnoughtus* are used*. (a-b)* Show right lateral views and *(c-d)* cranial views (reproduced from [10]).


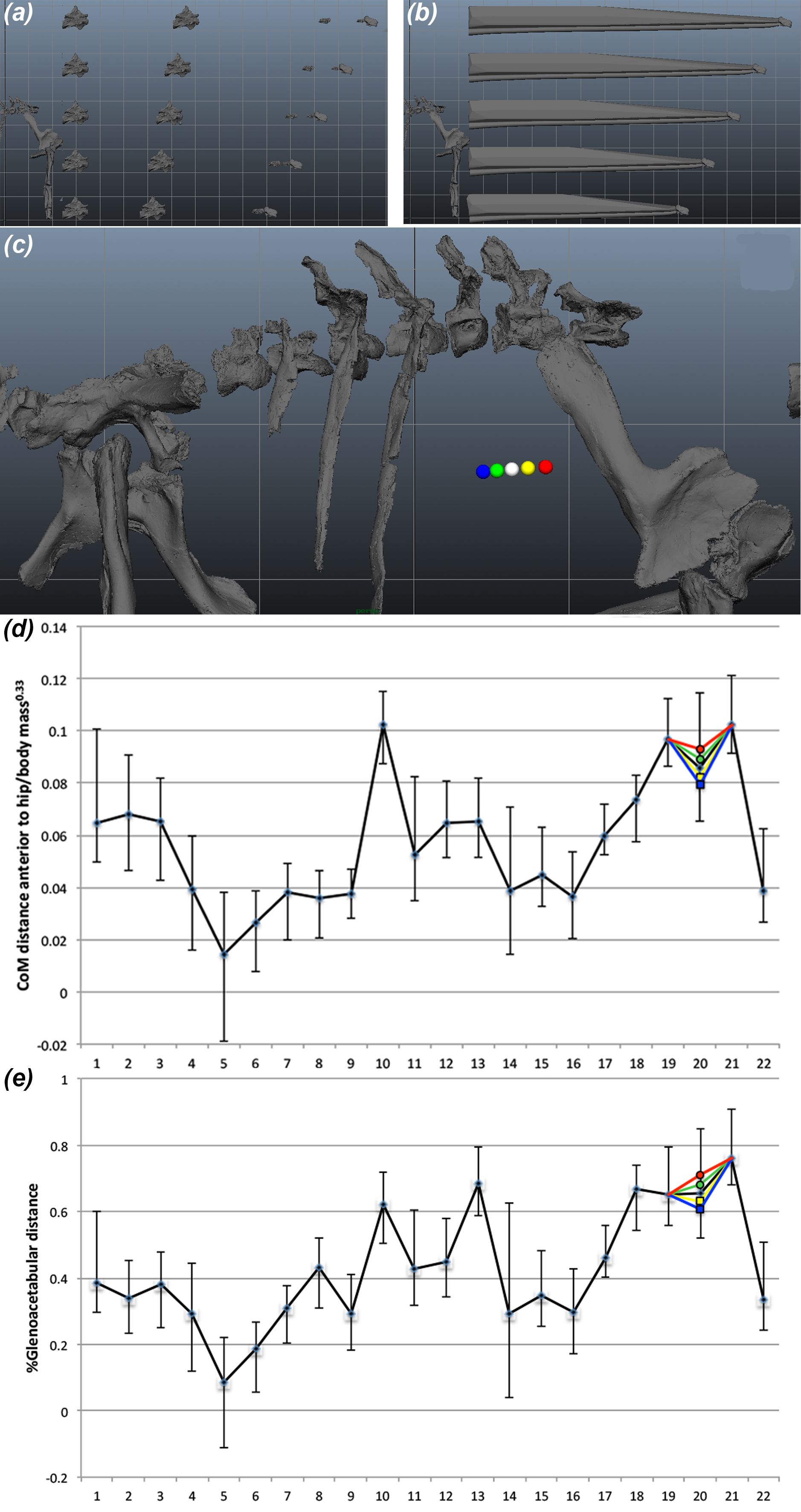


**Fig S11**. Alternative neck length *(a)* skeletal and *(b)* convex hull volume reconstructions in *Dreadnoughtus* (from top to bottom in *a-b* reconstructions are: Plus 20%, Plus 10%, the original reconstruction (based on Lacovara et al. [62]), Minus 10% and Minus 20% overall neck length), with the effect on overall CoM position shown *(c)* within the model (white sphere initial prediction [Fig 4], the blue and green spheres representing the more caudal positions given by reducing neck length by 20% and 10%, and yellow and red spheres the more cranial positions given by increasing neck length by 10% and 20%). *(d-e)* Raw CoM data with the initial model of *Dreadnoughtus* *(d)* normalized by body mass^0.33^ and *(e)* gleno-acetabular distance, with the colours of lines/data points matching the CoM positions of the respective models as shown in *(c)*.


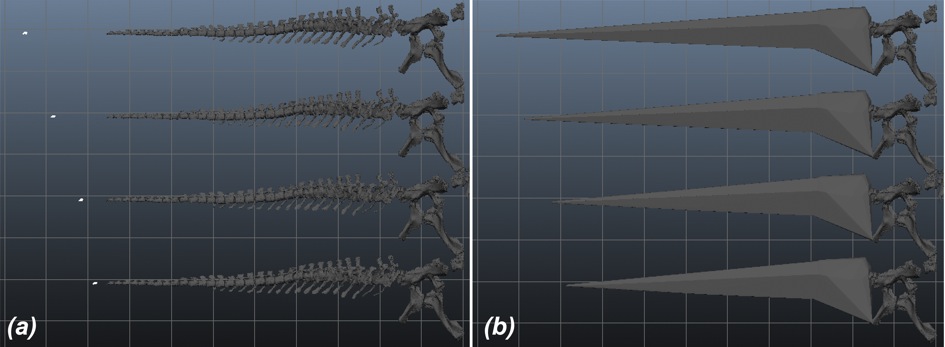


**Figure S12.** Alternative tail length *(a)* skeletal and *(b)* convex hull volume reconstructions in *Dreadnoughtus* From top to bottom in *a-b* reconstructions are: Plus 30%, Plus 20% Plus 10% (i.e. the original reconstruction used in Figures 4-7 and in [10] based on Lacovara et al. [62]) and Plus 5% models. In the skeletal reconstruction (a) the white bone represents a duplicate of the most distal caudal vertebrae preserved in the fossil, displaced further distally by the appropriate distance in each reconstruction.

**Supplementary Tables**

**Table S1.** Taxon and specimen information, including notes on skeletal completeness with completeness scores (percentages) for each sauropodomorph genera from Mannion and Upchurch [61].

| **Taxon** | **Specimen Number** | **Institution** | **Digitization method** | **Completeness metric [61] and notes** |
| --- | --- | --- | --- | --- |
| *Marasuchus lilloensis* | NHMUK R14101 | Natural History Museum, London, UK | Laser Surface Scan [13] | Ribcage Missing. Chest cavity assumed to be roughly ellipsoid, fitted to available landmarks: Pelvic depth, Width of pectoral girdle, curvature of dorsal vertebrae. |
| *Heterodontosaurus tucki* | UCMP 12916 | University of California Museum of Paleontology, Berkeley, USA | Laser Surface Scan [13] | Partial Ribcage Only. Chest reconstructed by fitting convex hull to available ribs and other landmarks (as above). |
| *Staurikosaurus pricei* | MCZ 1669 | Museum of Comparative Zoology, Harvard, USA | Laser Surface Scan [13] | Sculpted reconstruction by Orlando Grillo, based on type specimen MCZ 1669. |
| *Coelophysis bauri* | CMNH 10971 (Composite Mount) | Cleveland Museum of Natural History, USA | Laser Surface Scan [13] | Complete. Some (minor) elements sculpted when cast originally reconstructed. |
| *Plateosaurus engelhardti* | GPIT1 & GPIT2 | Eberhardt-Karls- Universitat Tubingen, Germany | CT Scan [13] | Near complete. Mostly GPIT1, some scaled elements from GPIT2 used, assembled by Heinrich Mallison (Humboldt MFN)  (100%) |
| *Lufengosaurus*  *huenei* | IVPP V15 | Institute of Vertebrate Paleontology & Paleoanthropology, Beijing, China | Photogrammetry | Near-complete  (88%) |
| *Cetiosaurus oxoniensis* | LCM G468.1968 | New Walk Museum & Art Gallery, Leicester, UK | Laser Surface Scan | Near-complete vertebral column, partial pelvis and femur  (43%) |
| *Patagosaurus fariasi* | PVL 4170 (cast mounted at MACN) | Museo Argentino de Ciencias Naturales ‘Bernardino Rivadavia’, Buenos Aires, Argentina | Photogrammetry | Cast based on nearly complete specimen PVL 4170  (34%) |
| *Mamenchisaurus sp.* | IVPP V456-V458 | Institute of Vertebrate Paleontology & Paleoanthropology, Beijing, China | Photogrammetry | Cast composite based largely on IVPP V456-V458  (72%) |
| *Jobaria tiguidensis* | MNN TIG3 | Australian Museum, Sydney, Australia | Photogrammetry | Near-complete cast  (95%) |
| *Apatosaurus louisae* | CM 3018 | Carnegie Museum of Natural History, Pittsburgh, USA | Photogrammetry | Near-complete  (97%) |
| *Barosaurus lentus* | AMNH 6341 | American Museum of Natural History, New York, USA | Photogrammetry | Near-complete  (65%) |
| *Diplodocus longus* | DMNS 1494 | Denver Museum of Nature and Science, Colorado, USA | Laser Surface Scan | Near-complete  (100%) |
| *Amargasaurus cazaui* | MACN N-15 | Museo Argentino de Ciencias Naturales ‘Bernardino Rivadavia’, Buenos Aires, Argentina | Photogrammetry | Cast based on near-complete specimen (missing distal tail and the manual and pedal elements)  (43%) |
| *Dicraeosaurus sp.* | MfN | Museum für Naturkunde, [Berlin](http://en.wikipedia.org/wiki/Berlin) | Laser Surface Scan | Near-complete  (70%) |
| *Camarasaurus lentus* | AMNH 664, and CM 11338 | N/A | CAD sculpted model [18] | Generated using data from AMNH 664, and CM 11338  (100%) |
| *Giraffatitan brancai* | MfN MB.R.2181 | Museum für Naturkunde, [Berlin](http://en.wikipedia.org/wiki/Berlin), Germany | Laser Surface Scan [9-10] | Near-complete  (90%) |
| *Sauroposeidon* (“*Paluxysaurus*”) *proteles* | FWMSH 93B-10 | Fort Worth Museum of Science and History, Texas, USA | Photogrammetry | Partial skeleton  (70%) |
| *Dreadnoughtus*  *schrani* | MPM-PV 1156 |  | Laser surface scan [10], [62] | Near-complete  (45%) |
| *Rapetosaurus krausei* | FMNH PR 2209 | Field Museum of Natural History, Chicago, USA | Photogrammetry | Near-complete (juvenile)  (48%) |
| *Neuquensaurus australis* | MLP-CS (several specimen numbers) | Museo de La Plata, Argentina | Photogrammetry | Near-complete, based on several individuals, cast plus original bones  (70%) |

**Table S2.** Taxa, minimum ages and basis for node ages. Note that our analyses require non-zero length branch lengths and so node ages used in analyses (where different) are shown in parentheses. MDS = more derived sauropodomorphs.

| **Taxon** | **Minimum age (Ma)** | **Basis for age** |
| --- | --- | --- |
| Archosauria | 249 | Earliest poposauroids [82] |
| Dinosauromorpha | 245 | *Nyasasaurus* [83] |
| *Marasuchus* | 241 | [84] |
| Dinosauria | 230 (232) | The age of Saurischia |
| *Heterodontosaurus* | 201 | [85] |
| Saurischia | 230 (231) | The age of Theropoda and Sauropodomorpha |
| Theropoda | 230 | The age of *Staurikosaurus* |
| *Staurikosaurus* | 230 | [86] |
| *Coelophysis* | 228 | [83] |
| Sauropodomorpha | 230 | The age of the Ischigualasto and Santa Maria formations [85] |
| *Plateosaurus* | 228 | [86] |
| Unnamed clade (*Lufengosaurus* + MDS) | 201 (202) | The age of *Lufengosaurus* |
| *Lufengosaurus* | 201 | [64-65] |
| Eusauropoda | 178.8 | Cañadón Asfalto Formation specimen [64-65] |
| Cetiosauridae | 174 (175) | The age of *Patagosaurus* |
| *Patagosaurus* | 174 | [65] |
| *Cetiosaurus* | 169 | [67] |
| Unnamed clade (*Mamenchisaurus* + MDS) | 174 | The age of Cetiosauridae |
| *Mamenchisaurus* | 164 | [2] |
| Unnamed clade (*Jobaria* + MDS) | 168 (169) | The age of *Jobaria* |
| *Jobaria* | 168 | [87] |
| Neosauropoda | 161 (164) | The age of Diplodocoidea and Macronaria |
| Diplodocoidea | 161 (163) | The age of Diplodocidae |
| Diplodocidae | 161 | Georgian diplodocid [88] |
| *Apatosaurus* | 157 | [2] |
| Diplodocinae | 157 (158) | The age of *Barosaurus* and *Diplodocus* |
| *Barosaurus* | 157 | [2] |
| *Diplodocus* | 157 | [2] |
| Dicraeosauridae | 161 | The age of Diplodocidae |
| *Dicraeosaurus* | 157 | [2] |
| *Amargasaurus* | 131 | [89] |
| Macronaria | 161 (163) | The age of Titanosauriformes and Diplodocoidea |
| *Camarasaurus* | 157 | [2] |
| Titanosauriformes | 161 (162) | ‘French *Bothriospondylus*’ [67] |
| *Giraffatitan* | 157 | [2] |
| Somphospondyli | 161 | The age of Brachiosauridae |
| *Sauroposeidon* | 126 | [44] |
| Titanosauria | 131 (132) | UK titanosaurs [90] |
| *Dreadnoughtus* | 83.6 | [62] |
| Lithostrotia | 131 | UK titanosaurs [90] |
| *Neuquensaurus* | 86.3 | [91] |
| *Rapetosaurus* | 72.1 | [92] |

**Table S3. Branch lengths leading to nodes.**

| **Node** | **Branch length (Myr)** |
| --- | --- |
| Dinosauromorpha | 4 |
| Dinosauria | 13 |
| Saurischia | 1 |
| Theropoda | 1 |
| Sauropodomorpha | 1 |
| Unnamed clade (*Lufengosaurus* + MDS) | 28 |
| Eusauropoda | 23.2 |
| Cetiosauridae | 3.8 |
| Unnamed clade (*Mamenchisaurus* + MDS) | 1 |
| Unnamed clade (*Jobaria* + MDS) | 5 |
| Neosauropoda | 5 |
| Diplodocoidea | 1 |
| Diplodocidae | 2 |
| Diplodocinae | 3 |
| Dicraeosauridae | 2 |
| Macronaria | 1 |
| Titanosauriformes | 1 |
| Somphospondyli | 1 |
| Titanosauria | 29 |
| Lithostrotia | 1 |

**Table S4.** Length of selected body segments in meters (GA, gleno-acetabular distance).

|  | Femur | Hindlimb | Humerus | Forelimb | GA distance | Tail | Neck |
| --- | --- | --- | --- | --- | --- | --- | --- |
| *Alligator* | 0.130 | 0.360 | 0.092 | 0.210 | 0.340 | 0.570 | 0.130 |
| *Marasuchus* | 0.053 | 0.202 | 0.032 | 0.105 | 0.119 | 0.248 | 0.040 |
| *Heterodontosaurus* | 0.108 | 0.382 | 0.080 | 0.193 | 0.240 | 0.364 | 0.102 |
| *Staurikosaurus* | 0.216 | 0.688 | 0.107 | 0.321 | 0.346 | 1.265 | 0.243 |
| *Coelophysis* | 0.172 | 0.506 | 0.097 | 0.242 | 0.437 | 1.400 | 0.318 |
| *Plateosaurus* | 0.570 | 1.410 | 0.350 | 0.850 | 1.140 | 2.560 | 0.980 |
| *Lufengosaurus* | 0.716 | 1.787 | 0.266 | 0.600 | 1.400 | 3.100 | 1.650 |
| *Cetiosaurus* | 1.380 | 2.570 | 0.782 | 1.757 | 1.540 | 7.020 | 3.970 |
| *Patagosaurus* | 1.279 | 2.450 | 1.010 | 2.170 | 2.670 | 9.320 | 3.320 |
| *Mamenchisaurus* | 1.200 | 2.370 | 1.060 | 2.060 | 3.750 | 5.480 | 7.600 |
| *Jobaria* | 1.860 | 3.350 | 1.340 | 3.040 | 2.964 | 8.270 | 3.890 |
| *Apatosaurus* | 1.760 | 3.655 | 1.140 | 2.500 | 4.280 | 9.970 | 5.970 |
| *Barosaurus* | 1.660 | 2.740 | 1.740 | 3.050 | 2.499 | 13.200 | 9.670 |
| *Diplodocus* | 1.583 | 2.893 | 0.997 | 2.074 | 2.851 | 13.123 | 5.306 |
| *Amargasaurus* | 0.950 | 1.997 | 0.670 | 1.440 | 1.969 | 4.550 | 2.230 |
| *Dicraeosaurus* | 1.216 | 2.237 | 0.749 | 1.563 | 2.224 | 8.330 | 2.502 |
| *Camarasaurus* | 1.550 | 2.820 | 1.080 | 2.290 | 2.980 | 6.650 | 3.730 |
| *Giraffatitan* | 1.890 | 3.630 | 2.160 | 4.130 | 3.171 | 7.600 | 8.860 |
| *Sauroposeidon* | 1.490 | 2.900 | 1.190 | 2.610 | 3.330 | 7.270 | 8.040 |
| *Dreadnoughtus* | 1.910 | 3.290 | 1.600 | 2.990 | 3.890 | 7.730 | 10.580 |
| *Rapetosaurus* | 0.685 | 1.310 | 0.492 | 1.070 | 1.368 | 1.760 | 3.342 |
| *Neuquensaurus* | 0.680 | 1.600 | 0.398 | 0.987 | 1.357 | 5.480 | 1.830 |

**Table S5.** Body segment and whole model mass properties from *Alligator* convex hull model

| **Body segments** | **Volume** | **Density** | **Mass** | **CoM X** | **CoM Y** |
| --- | --- | --- | --- | --- | --- |
| Head | 0.001192 | 1000 | 1.192 | 0.249 | 0.324 |
| Neck | 0.000128 | 1000 | 0.128 | 0.424 | 0.345 |
| Trunk | 0.003996 | 1000 | 3.996 | 0.182 | 0.345 |
| Tail | 0.000647 | 1000 | 0.647 | -0.182 | 0.346 |
| Humerus | 0.000024 | 1000 | 0.024 | 0.353 | 0.294 |
| Forearm | 0.000014 | 1000 | 0.014 | 0.358 | 0.221 |
| Hand | 0.000016 | 1000 | 0.016 | 0.350 | 0.152 |
| Humerus | 0.000024 | 1000 | 0.024 | 0.353 | 0.294 |
| Forearm | 0.000014 | 1000 | 0.014 | 0.358 | 0.221 |
| Hand | 0.000016 | 1000 | 0.016 | 0.350 | 0.152 |
| Femur | 0.000108 | 1000 | 0.108 | 0.019 | 0.288 |
| Crus | 0.000111 | 1000 | 0.111 | 0.025 | 0.157 |
| Foot | 0.000100 | 1000 | 0.100 | 0.022 | 0.033 |
| Femur | 0.000108 | 1000 | 0.108 | 0.019 | 0.288 |
| Crus | 0.000111 | 1000 | 0.111 | 0.025 | 0.157 |
| Foot | 0.000100 | 1000 | 0.100 | 0.022 | 0.033 |
|  |  |  |  |  |  |
| Axial total | 0.005963 | 1000 | 5.963 | 0.161 | 0.341 |
| Hind limb total | 0.000319 | 1000 | 0.319 | 0.022 | 0.163 |
| Fore limb total | 0.000054 | 1000 | 0.054 | 0.353 | 0.233 |
| Whole body | 0.006709 | 1000 | 6.709 | 0.151 | 0.322 |
|  |  |  |  |  |  |
| **Respiratory structures** |  |  |  |  |  |
| Head | 8.38249e-05 | 1000 | 0.084 | 0.569 | 0.349 |
| Neck | 2.58228e-05 | 1000 | 0.026 | 0.418 | 0.334 |
| Trunk | 0.0001991 | 1000 | 0.199 | 0.257 | 0.345 |

**Table S6.** Body segment and whole model mass properties from *Marasuchus* convex hull model

| **Body segments** | **Volume** | **Density** | **Mass** | **CoM X** | **CoM Y** |
| --- | --- | --- | --- | --- | --- |
| Head | 0.000009 | 1000 | 0.009 | 0.18 | 0.179 |
| Neck | 0.000003 | 1000 | 0.003 | 0.15 | 0.181 |
| Trunk | 0.00011 | 1000 | 0.11 | 0.052 | 0.17 |
| Tail | 0.000021 | 1000 | 0.021 | -0.094 | 0.174 |
| Humerus | 0.000001 | 1000 | 0.001 | 0.119 | 0.144 |
| Forearm | 0.000002 | 1000 | 0.002 | 0.119 | 0.107 |
| Hand | 0.000002 | 1000 | 0.002 | 0.123 | 0.073 |
| Humerus | 0.000001 | 1000 | 0.001 | 0.119 | 0.144 |
| Forearm | 0.000002 | 1000 | 0.002 | 0.119 | 0.107 |
| Hand | 0.000002 | 1000 | 0.002 | 0.123 | 0.073 |
| Femur | 0.000003 | 1000 | 0.003 | -0.002 | 0.181 |
| Crus | 0.000003 | 1000 | 0.003 | -0.001 | 0.095 |
| Foot | 0.000004 | 1000 | 0.004 | 0.0095 | 0.0275 |
| Femur | 0.000003 | 1000 | 0.003 | -0.002 | 0.181 |
| Crus | 0.000003 | 1000 | 0.003 | -0.001 | 0.095 |
| Foot | 0.000004 | 1000 | 0.004 | 0.0095 | 0.0275 |
|  |  |  |  |  |  |
| Axial total | 0.000143 | 1000 | 0.143 | 0.0407 | 0.1714 |
| Hind limb total | 0.00001 | 1000 | 0.01 | 0.0029 | 0.0938 |
| Fore limb total | 0.000005 | 1000 | 0.005 | 0.1206 | 0.1008 |
| Whole body | 0.000173 | 1000 | 0.173 | 0.0409 | 0.1583 |
|  |  |  |  |  |  |
| **Respiratory structures** |  |  |  |  |  |
| Head | 3.805E-08 | 1000 | 0.00003805 | 0.18 | 0.181 |
| Neck | 1.65254E-05 | 1000 | 0.0165254 | 0.075 | 0.171 |
| Trunk | 5.964E-08 | 1000 | 0.00005964 | 0.147 | 0.177 |

**Table S7.** Body segment and whole model mass properties from *Heterodontosaurus* convex hull model.

| **Body segments** | **Volume** | **Density** | **Mass** | **CoM X** | **CoM Y** |
| --- | --- | --- | --- | --- | --- |
| Head | 0.000212 | 1000 | 0.212 | 0.366 | 0.35 |
| Neck | 0.000026 | 1000 | 0.026 | 0.285 | 0.357 |
| Trunk | 0.001706 | 1000 | 1.706 | 0.112 | 0.327 |
| Tail | 0.000327 | 1000 | 0.327 | -0.188 | 0.345 |
| Humerus | 0.000012 | 1000 | 0.012 | 0.24 | 0.252 |
| Forearm | 0.000006 | 1000 | 0.006 | 0.239 | 0.18 |
| Hand | 0.000005 | 1000 | 0.005 | 0.238 | 0.13 |
| Humerus | 0.000012 | 1000 | 0.012 | 0.24 | 0.252 |
| Forearm | 0.000006 | 1000 | 0.006 | 0.239 | 0.18 |
| Hand | 0.000005 | 1000 | 0.005 | 0.238 | 0.13 |
| Femur | 0.000026 | 1000 | 0.026 | 0 | 0.297 |
| Crus | 0.00005 | 1000 | 0.05 | -0.002 | 0.181 |
| Foot | 0.000043 | 1000 | 0.043 | 0.0028 | 0.106 |
| Femur | 0.000026 | 1000 | 0.026 | 0 | 0.297 |
| Crus | 0.00005 | 1000 | 0.05 | -0.002 | 0.181 |
| Foot | 0.000043 | 1000 | 0.043 | 0.0028 | 0.106 |
|  |  |  |  |  |  |
| Axial total | 0.002271 | 1000 | 2.271 | 0.0945 | 0.3321 |
| Hind limb total | 0.000119 | 1000 | 0.119 | -0.0021 | 0.1485 |
| Fore limb total | 0.000023 | 1000 | 0.023 | 0.2393 | 0.2067 |
| Whole body | 0.002555 | 1000 | 2.555 | 0.0882 | 0.3142 |
|  |  |  |  |  |  |
| **Respiratory structures** |  |  |  |  |  |
| Head | 2.6829E-06 | 1000 | 0.003 | 0.353 | 0.362 |
| Neck | 0.0005 | 1000 | 0.500 | 0.132 | 0.323 |
| Trunk | 0.00001574 | 1000 | 0.016 | 0.279 | 0.339 |

**Table S8.** Body segment and whole model mass properties from *Staurikosaurus* convex hull model.

| **Body segments** | **Volume** | **Density** | **Mass** | **CoM X** | **CoM Y** |
| --- | --- | --- | --- | --- | --- |
| Head | 0.000828 | 1000 | 0.828 | 0.734 | 0.678 |
| Neck | 0.000547 | 1000 | 0.547 | 0.496 | 0.675 |
| Trunk | 0.008665 | 1000 | 8.665 | 0.167 | 0.626 |
| Tail | 0.002 | 1000 | 2.308 | -0.418 | 0.639 |
| Humerus | 0.000062 | 1000 | 0.062 | 0.348 | 0.549 |
| Forearm | 0.000034 | 1000 | 0.034 | 0.352 | 0.464 |
| Hand | 0.000049 | 1000 | 0.049 | 0.356 | 0.36 |
| Humerus | 0.000062 | 1000 | 0.062 | 0.348 | 0.549 |
| Forearm | 0.000034 | 1000 | 0.034 | 0.352 | 0.464 |
| Hand | 0.000049 | 1000 | 0.049 | 0.356 | 0.36 |
| Femur | 0.000248 | 1000 | 0.248 | 0.001 | 0.549 |
| Crus | 0.000338 | 1000 | 0.338 | 0 | 0.307 |
| Foot | 0.000372 | 1000 | 0.372 | 0.0351 | 0.0742 |
| Femur | 0.000248 | 1000 | 0.248 | 0.001 | 0.549 |
| Crus | 0.000338 | 1000 | 0.338 | 0 | 0.307 |
| Foot | 0.000372 | 1000 | 0.372 | 0.0351 | 0.0742 |
|  |  |  |  |  |  |
| Axial total | 0.01204 | 1000 | 12.348 | 0.1103 | 0.6341 |
| Hind limb total | 0.000958 | 1000 | 0.958 | 0.0139 | 0.2792 |
| Fore limb total | 0.000145 | 1000 | 0.145 | 0.3516 | 0.4652 |
| Whole body | 0.014246 | 1000 | 14.554 | 0.1024 | 0.5840 |
|  |  |  |  |  |  |
| **Respiratory structures** |  |  |  |  |  |
| Head | 0.00003765 | 1000 | 0.0377 | 0.721 | 0.701 |
| Neck | 0.0014 | 1000 | 1.400 | 0.215 | 0.641 |
| Trunk | 0.000051 | 1000 | 0.051 | 0.524 | 0.671 |

**Table S9.** Body segment and whole model mass properties from *Coelophysis* convex hull model.

| **Body segments** | **Volume** | **Density** | **Mass** | **CoM X** | **CoM Y** |
| --- | --- | --- | --- | --- | --- |
| Head | 0.000589 | 1000 | 0.589 | 0.861 | 0.535 |
| Neck | 0.000389 | 1000 | 0.389 | 0.6 | 0.546 |
| Trunk | 0.008484 | 1000 | 8.484 | 0.181 | 0.514 |
| Tail | 0.004 | 1000 | 4.053 | -0.473 | 0.544 |
| Humerus | 0.000017 | 1000 | 0.017 | 0.419 | 0.426 |
| Forearm | 0.000021 | 1000 | 0.021 | 0.417 | 0.318 |
| Hand | 0.000017 | 1000 | 0.017 | 0.408 | 0.253 |
| Humerus | 0.000017 | 1000 | 0.017 | 0.419 | 0.426 |
| Forearm | 0.000021 | 1000 | 0.021 | 0.417 | 0.318 |
| Hand | 0.000017 | 1000 | 0.017 | 0.408 | 0.253 |
| Femur | 0.000113 | 1000 | 0.113 | -0.009 | 0.474 |
| Crus | 0.000144 | 1000 | 0.144 | -0.013 | 0.308 |
| Foot | 0.000103 | 1000 | 0.103 | 0.003 | 0.101 |
| Femur | 0.000113 | 1000 | 0.113 | -0.009 | 0.474 |
| Crus | 0.000144 | 1000 | 0.144 | -0.013 | 0.308 |
| Foot | 0.000103 | 1000 | 0.103 | 0.003 | 0.101 |
|  |  |  |  |  |  |
| Axial total | 0.013462 | 1000 | 13.515 | 0.027 | 0.525 |
| Hind limb total | 0.00036 | 1000 | 0.360 | -0.007 | 0.301 |
| Fore limb total | 0.000055 | 1000 | 0.055 | 0.415 | 0.331 |
| Whole body | 0.014292 | 1000 | 14.345 | 0.028 | 0.512 |
|  |  |  |  |  |  |
| **Respiratory structures** |  |  |  |  |  |
| Head | 0.000028 | 1000 | 0.0280 | 0.85 | 0.541 |
| Neck | 0.0017 | 1000 | 1.700 | 0.264 | 0.536 |
| Trunk | 0.00007 | 1000 | 0.070 | 0.628 | 0.529 |

**Table S10.** Body segment and whole model mass properties from *Plateosaurus* convex hull model.

| **Body segments** | **Volume** | **Density** | **Mass** | **CoM X** | **CoM Y** |
| --- | --- | --- | --- | --- | --- |
| Head | 0.004278 | 1000 | 4.278 | 2.469 | 1.306 |
| Neck | 0.007209 | 1000 | 7.209 | 1.773 | 1.374 |
| Trunk | 0.364117 | 1000 | 364.117 | 0.59 | 1.239 |
| Tail | 0.083 | 1000 | 82.591 | -0.923 | 1.315 |
| Humerus | 0.002555 | 1000 | 2.555 | 1.211 | 0.816 |
| Forearm | 0.000971 | 1000 | 0.971 | 1.223 | 0.518 |
| Hand | 0.001305 | 1000 | 1.305 | 1.22 | 0.294 |
| Humerus | 0.002555 | 1000 | 2.555 | 1.211 | 0.816 |
| Forearm | 0.000971 | 1000 | 0.971 | 1.223 | 0.518 |
| Hand | 0.001305 | 1000 | 1.305 | 1.22 | 0.294 |
| Femur | 0.007323 | 1000 | 7.323 | 0.012 | 1.107 |
| Crus | 0.007496 | 1000 | 7.496 | 0.026 | 0.591 |
| Foot | 0.004767 | 1000 | 4.767 | 0.060 | 0.151 |
| Femur | 0.007323 | 1000 | 7.323 | 0.012 | 1.107 |
| Crus | 0.007496 | 1000 | 7.496 | 0.026 | 0.591 |
| Foot | 0.004767 | 1000 | 4.767 | 0.060 | 0.151 |
|  |  |  |  |  |  |
| Axial total | 0.458604 | 1000 | 458.195 | 0.353 | 1.255 |
| Hind limb total | 0.019586 | 1000 | 19.586 | 0.029 | 0.677 |
| Fore limb total | 0.004831 | 1000 | 4.831 | 1.216 | 0.615 |
| Whole body | 0.507438 | 1000 | 507.029 | 0.345 | 1.199 |
|  |  |  |  |  |  |
| **Respiratory structures** |  |  |  |  |  |
| Head | 0.000271 | 1000 | 0.271 | 2.462 | 1.354 |
| Neck | 0.112302 | 1000 | 112.302 | 0.776 | 1.301 |
| Trunk | 0.002939 | 1000 | 2.939 | 1.672 | 1.333 |

**Table S11.** Body segment and whole model mass properties from *Lufengosaurus* convex hull model.

| **Body segments** | **Volume** | **Density** | **Mass** | **CoM X** | **CoM Y** |
| --- | --- | --- | --- | --- | --- |
| Head | 0.007117 | 1000 | 7.117 | 2.768 | 1.027 |
| Neck | 0.054518 | 1000 | 54.518 | 1.707 | 1.041 |
| Trunk | 1.02872 | 1000 | 1028.72 | -0.028 | 0.921 |
| Tail | 0.161707 | 1000 | 161.707 | -2.046 | 0.914 |
| Humerus | 0.003465 | 1000 | 3.465 | 0.555 | 0.381 |
| Forearm | 0.004861 | 1000 | 4.861 | 0.546 | 0.167 |
| Hand | 0.002272 | 1000 | 2.272 | 0.559 | -0.014 |
| Humerus | 0.003465 | 1000 | 3.465 | 0.555 | 0.381 |
| Forearm | 0.004861 | 1000 | 4.861 | 0.546 | 0.167 |
| Hand | 0.002272 | 1000 | 2.272 | 0.559 | -0.014 |
| Femur | 0.023792 | 1000 | 23.792 | -0.735 | 0.74 |
| Crus | 0.017588 | 1000 | 17.588 | -0.775 | 0.127 |
| Foot | 0.007099 | 1000 | 7.099 | -0.792 | -0.383 |
| Femur | 0.023792 | 1000 | 23.792 | -0.735 | 0.74 |
| Crus | 0.017588 | 1000 | 17.588 | -0.775 | 0.127 |
| Foot | 0.007099 | 1000 | 7.099 | -0.792 | -0.383 |
|  |  |  |  |  |  |
| Axial total | 1.252062 | 1000 | 1252.062 | -0.197 | 0.926 |
| Hind limb total | 0.048479 | 1000 | 48.479 | -0.758 | 0.353 |
| Fore limb total | 0.010598 | 1000 | 10.598 | 0.552 | 0.198 |
| Whole body | 1.370216 | 1000 | 1370.216 | -0.226 | 0.874 |
|  |  |  |  |  |  |
| **Respiratory structures** |  |  |  |  |  |
| Head | 0.000418 | 1000 | 0.418 | 2.725 | 1.047 |
| Neck | 0.142458 | 1000 | 142.458 | 0.26 | 0.988 |
| Trunk | 0.006075 | 1000 | 6.075 | 1.644 | 0.973 |

**Table S12.** Body segment and whole model mass properties from *Cetiosaurus* convex hull model.

| **Body segments** | **Volume** | **Density** | **Mass** | **CoM X** | **CoM Y** |
| --- | --- | --- | --- | --- | --- |
| Head | 0.019 | 1000 | 19.356 | 5.86 | 2.816 |
| Neck | 0.392 | 1000 | 392.371 | 2.972 | 2.835 |
| Trunk | 3.878 | 1000 | 3877.563 | 0.373 | 2.57 |
| Tail | 0.719 | 1000 | 718.391 | -2.582 | 2.594 |
| Humerus | 0.068 | 1000 | 67.621 | 1.532 | 1.628 |
| Forearm | 0.049 | 1000 | 49.173 | 1.536 | 0.88 |
| Hand | 0.029 | 1000 | 29.315 | 1.592 | 0.416 |
| Humerus | 0.068 | 1000 | 67.621 | 1.532 | 1.628 |
| Forearm | 0.049 | 1000 | 49.173 | 1.536 | 0.88 |
| Hand | 0.029 | 1000 | 29.315 | 1.592 | 0.416 |
| Femur | 0.154 | 1000 | 154.446 | -0.606 | 2.063 |
| Crus | 0.092 | 1000 | 92.196 | -0.649 | 0.915 |
| Foot | 0.025 | 1000 | 25.427 | -0.572 | 0.276 |
| Femur | 0.154 | 1000 | 154.446 | -0.606 | 2.063 |
| Crus | 0.092 | 1000 | 92.196 | -0.649 | 0.915 |
| Foot | 0.025 | 1000 | 25.427 | -0.572 | 0.276 |
|  |  |  |  |  |  |
| Axial total | 5.008 | 1000 | 5007.681 | 0.174 | 2.595 |
| Hind limb total | 0.271 | 1000 | 272.069 | -0.617 | 1.507 |
| Fore limb total | 0.146 | 1000 | 146.109 | 1.545 | 1.133 |
| Whole body | 5.842 | 1000 | 5844.037 | 0.169 | 2.421 |
|  |  |  |  |  |  |
| **Respiratory structures** |  |  |  |  |  |
| Head | 0.001021 | 1000 | 1.021 | 5.803 | 2.873 |
| Neck | 0.499672 | 1000 | 499.672 | 0.728 | 2.714 |
| Trunk | 0.08394 | 1000 | 83.94 | 2.849 | 2.702 |

**Table S13.** Body segment and whole model mass properties from *Patagosaurus* convex hull model.

| **Body segments** | **Volume** | **Density** | **Mass** | **CoM X** | **CoM Y** |
| --- | --- | --- | --- | --- | --- |
| Head | 0.076351 | 1000 | 76.351 | 4.954 | 3.169 |
| Neck | 0.357408 | 1000 | 357.408 | 2.814 | 3.229 |
| Trunk | 6.253757 | 1000 | 6253.757 | -0.229 | 2.868 |
| Tail | 1.149526 | 1000 | 1149.526 | -3.310 | 2.931 |
| Humerus | 0.064315 | 1000 | 64.315 | 1.271 | 1.912 |
| Forearm | 0.042894 | 1000 | 42.894 | 1.285 | 0.934 |
| Hand | 0.049893 | 1000 | 49.893 | 1.372 | 0.312 |
| Humerus | 0.064315 | 1000 | 64.315 | 1.271 | 1.912 |
| Forearm | 0.042894 | 1000 | 42.894 | 1.285 | 0.934 |
| Hand | 0.049893 | 1000 | 49.893 | 1.372 | 0.312 |
| Femur | 0.113799 | 1000 | 113.799 | -1.383 | 2.144 |
| Crus | 0.08265 | 1000 | 82.65 | -1.44 | 1.067 |
| Foot | 0.019864 | 1000 | 19.864 | -1.409 | 0.433 |
| Femur | 0.113799 | 1000 | 113.799 | -1.383 | 2.144 |
| Crus | 0.08265 | 1000 | 82.65 | -1.44 | 1.067 |
| Foot | 0.019864 | 1000 | 19.864 | -1.409 | 0.433 |
|  |  |  |  |  |  |
| Axial total | 7.837042 | 1000 | 7837.042 | -0.492 | 2.897 |
| Hind limb total | 0.216313 | 1000 | 216.313 | -1.407 | 1.575 |
| Fore limb total | 0.157102 | 1000 | 157.102 | 1.307 | 1.137 |
| Whole body | 8.583872 | 1000 | 8583.872 | -0.472 | 2.766 |
|  |  |  |  |  |  |
| **Respiratory structures** |  |  |  |  |  |
| Head | 0.005051 | 1000 | 5.051 | 4.900 | 3.279 |
| Neck | 0.946148 | 1000 | 946.148 | 0.165 | 2.788 |
| Trunk | 0.040155 | 1000 | 40.155 | 3.165 | 2.989 |

**Table S14.** Body segment and whole model mass properties from *Mamenchisaurus* convex hull model.

| **Body segments** | **Volume** | **Density** | **Mass** | **CoM X** | **CoM Y** |
| --- | --- | --- | --- | --- | --- |
| Head | 0.076351 | 1000 | 76.351 | 10.414 | 2.799 |
| Neck | 2.074853 | 1000 | 2074.853 | 5.354 | 2.815 |
| Trunk | 8.782424 | 1000 | 8782.424 | 0.104 | 2.679 |
| Tail | 0.62881 | 1000 | 628.81 | -3.887 | 2.99 |
| Humerus | 0.059678 | 1000 | 59.678 | 2.101 | 1.678 |
| Forearm | 0.026724 | 1000 | 26.724 | 2.087 | 0.704 |
| Hand | 0.006259 | 1000 | 6.259 | 2.13 | 0.187 |
| Humerus | 0.059678 | 1000 | 59.678 | 2.101 | 1.678 |
| Forearm | 0.026724 | 1000 | 26.724 | 2.087 | 0.704 |
| Hand | 0.006259 | 1000 | 6.259 | 2.13 | 0.187 |
| Femur | 0.086291 | 1000 | 86.291 | -1.675 | 1.956 |
| Crus | 0.03219 | 1000 | 32.19 | -1.672 | 0.97 |
| Foot | 0.039279 | 1000 | 39.279 | -1.512 | 0.299 |
| Femur | 0.086291 | 1000 | 86.291 | -1.675 | 1.956 |
| Crus | 0.03219 | 1000 | 32.19 | -1.672 | 0.97 |
| Foot | 0.039279 | 1000 | 39.279 | -1.512 | 0.299 |
|  |  |  |  |  |  |
| Axial total | 11.562 | 1000 | 11562.438 | 0.897 | 2.721 |
| Hind limb total | 0.158 | 1000 | 157.76 | -1.634 | 1.342 |
| Fore limb total | 0.093 | 1000 | 92.661 | 2.099 | 1.296 |
| Whole body | 12.063 | 1000 | 12063.280 | 0.849 | 2.663 |
|  |  |  |  |  |  |
| **Respiratory structures** |  |  |  |  |  |
| Head | 0.005051 | 1000 | 5.051 | 10.401 | 2.904 |
| Neck | 0.468044 | 1000 | 468.044 | 6.062 | 2.526 |
| Trunk | 1.825384 | 1000 | 1825.384 | 0.405 | 2.708 |

**Table S15.** Body segment and whole model mass properties from *Jobaria* convex hull model.

| **Body segments** | **Volume** | **Density** | **Mass** | **CoM X** | **CoM Y** |
| --- | --- | --- | --- | --- | --- |
| Head | 0.185 | 1000 | 184.896 | 5.839 | 4.335 |
| Neck | 1.264 | 1000 | 1264.496 | 3.186 | 4.405 |
| Trunk | 9.367 | 1000 | 9366.898 | -0.208 | 3.726 |
| Tail | 0.789 | 1000 | 789.157 | -4.135 | 3.637 |
| Humerus | 0.154 | 1000 | 153.608 | 1.545 | 2.758 |
| Forearm | 0.158 | 1000 | 157.584 | 1.5 | 1.311 |
| Hand | 0.046 | 1000 | 46.355 | 1.475 | 0.46 |
| Humerus | 0.154 | 1000 | 153.608 | 1.545 | 2.758 |
| Forearm | 0.158 | 1000 | 157.584 | 1.5 | 1.311 |
| Hand | 0.046 | 1000 | 46.355 | 1.475 | 0.46 |
| Femur | 0.209 | 1000 | 209.137 | -1.339 | 2.542 |
| Crus | 0.163 | 1000 | 162.8 | -1.363 | 1.198 |
| Foot | 0.054 | 1000 | 53.837 | -1.386 | 0.43 |
| Femur | 0.209 | 1000 | 209.137 | -1.339 | 2.542 |
| Crus | 0.163 | 1000 | 162.8 | -1.363 | 1.198 |
| Foot | 0.054 | 1000 | 53.837 | -1.386 | 0.43 |
|  |  |  |  |  |  |
| Axial total | 11.605 | 1000 | 11605.447 | -0.009 | 3.804 |
| Hind limb total | 0.426 | 1000 | 425.774 | -1.354 | 1.761 |
| Fore limb total | 0.358 | 1000 | 357.547 | 1.516 | 1.822 |
| Whole body | 13.173 | 1000 | 13172.089 | -0.007 | 3.562 |
|  |  |  |  |  |  |
| **Respiratory structures** |  |  |  |  |  |
| Head | 0.007 | 1000 | 7.475 | 5.803 | 4.498 |
| Neck | 1.662 | 1000 | 1662.434 | 0.19 | 4.02 |
| Trunk | 0.093 | 1000 | 92.723 | 3.491 | 4.122 |

**Table S16.** Body segment and whole model mass properties from *Apatosaurus* convex hull model.

| **Body segments** | **Volume** | **Density** | **Mass** | **CoM X** | **CoM Y** |
| --- | --- | --- | --- | --- | --- |
| Head | 0.023 | 1000 | 23.459 | 8.252 | 4.436 |
| Neck | 2.615 | 1000 | 2615.156 | 4.8185 | 4.3486 |
| Trunk | 20.118 | 1000 | 20187.653 | -0.049 | 4.008 |
| Tail | 1.861 | 1000 | 1861.204 | -4.825 | 4.012 |
| Humerus | 0.232 | 1000 | 232.339 | 2.283 | 3.12 |
| Forearm | 0.103 | 1000 | 103.013 | 2.39 | 2.16 |
| Hand | 0.026 | 1000 | 25.956 | 2.546 | 1.497 |
| Humerus | 0.275313 | 1000 | 275.313 | 2.434 | 3.06 |
| Forearm | 0.103 | 1000 | 103.013 | 2.39 | 2.16 |
| Hand | 0.026 | 1000 | 25.956 | 2.546 | 1.497 |
| Femur | 0.351 | 1000 | 351.27 | -1.889 | 3.273 |
| Crus | 0.209 | 1000 | 208.568 | -1.809 | 1.885 |
| Foot | 0.084621 | 1000 | 84.621 | -1.635 | 0.706 |
| Femur | 0.351 | 1000 | 351.27 | -1.889 | 3.273 |
| Crus | 0.209 | 1000 | 208.568 | -1.809 | 1.885 |
| Foot | 0.084621 | 1000 | 84.621 | -1.635 | 0.706 |
|  |  |  |  |  |  |
| Axial total | 24.617 | 1000 | 24687.472 | 0.114 | 4.045 |
| Hind limb total | 0.645 | 1000 | 644.459 | -1.830 | 2.487 |
| Fore limb total | 0.361 | 1000 | 361.308 | 2.332 | 2.730 |
| Whole body | 26.628 | 1000 | 26699.006 | 0.086 | 3.938 |
|  |  |  |  |  |  |
| **Respiratory structures** |  |  |  |  |  |
| Head | 0.0010 | 1000 | 0.991 | 8.207 | 4.482 |
| Neck | 4.603 | 1000 | 4602.856 | 0.297 | 3.908 |
| Trunk | 0.292 | 1000 | 291.948 | 4.865 | 4.099 |

**Table S17.** Body segment and whole model mass properties from *Barosaurus* convex hull model.

| **Body segments** | **Volume** | **Density** | **Mass** | **CoM X** | **CoM Y** |
| --- | --- | --- | --- | --- | --- |
| Head | 0.085 | 1000 | 84.790 | 12.085 | 4.043 |
| Neck | 3.586 | 1000 | 3586.466 | 5.798 | 4.012 |
| Trunk | 10.174 | 1000 | 10173.56 | 0.166 | 3.308 |
| Tail | 1.528 | 1000 | 1528.423 | -4.877 | 3.365 |
| Humerus | 0.161 | 1000 | 160.854 | 1.951 | 2.11 |
| Forearm | 0.13 | 1000 | 130.113 | 1.972 | 1.103 |
| Hand | 0.02 | 1000 | 19.808 | 1.966 | 0.363 |
| Humerus | 0.161 | 1000 | 160.854 | 1.951 | 2.11 |
| Forearm | 0.13 | 1000 | 130.113 | 1.972 | 1.103 |
| Hand | 0.02 | 1000 | 19.808 | 1.966 | 0.363 |
| Femur | 0.164 | 1000 | 163.719 | -0.797 | 2.677 |
| Crus | 0.098 | 1000 | 97.571 | -0.771 | 1.51 |
| Foot | 0.077 | 1000 | 77.147 | -0.715 | 0.853 |
| Femur | 0.164 | 1000 | 163.719 | -0.797 | 2.677 |
| Crus | 0.098 | 1000 | 97.571 | -0.771 | 1.51 |
| Foot | 0.077 | 1000 | 77.147 | -0.715 | 0.853 |
|  |  |  |  |  |  |
| Axial total | 15.373 | 1000 | 15373.239 | 1.044 | 3.482 |
| Hind limb total | 0.339 | 1000 | 338.437 | -0.771 | 1.925 |
| Fore limb total | 0.311 | 1000 | 310.775 | 1.961 | 1.577 |
| Whole body | 16.673 | 1000 | 16671.663 | 1.008 | 3.344 |
|  |  |  |  |  |  |
| **Respiratory structures** |  |  |  |  |  |
| Head | 0.0037 | 1000 | 3.681 | 12.067 | 4.056 |
| Neck | 1.4852 | 1000 | 1485.208 | 0.956 | 3.280 |
| Trunk | 0.3674 | 1000 | 367.414 | 6.448 | 3.790 |

**Table S18.** Body segment and whole model mass properties from *Diplodocus* convex hull model.

| **Body segments** | **Volume** | **Density** | **Mass** | **CoM X** | **CoM Y** |
| --- | --- | --- | --- | --- | --- |
| Head | 0.027 | 1000 | 26.776 | 8.501 | 3.13 |
| Neck | 0.788 | 1000 | 788.264 | 4.859 | 3.214 |
| Trunk | 6.948 | 1000 | 6947.948 | 1.169 | 2.817 |
| Tail | 2.884 | 1000 | 1301.851 | -2.769 | 3.323 |
| Humerus | 0.071 | 1000 | 70.912 | 2.845 | 1.733 |
| Forearm | 0.029 | 1000 | 28.975 | 2.846 | 0.844 |
| Hand | 0.014 | 1000 | 14.403 | 2.869 | 0.274 |
| Humerus | 0.071 | 1000 | 70.912 | 2.845 | 1.733 |
| Forearm | 0.029 | 1000 | 28.975 | 2.846 | 0.844 |
| Hand | 0.014 | 1000 | 14.403 | 2.869 | 0.274 |
| Femur | 0.150 | 1000 | 149.62 | -0.036 | 2.258 |
| Crus | 0.092 | 1000 | 91.697 | -0.057 | 1.009 |
| Foot | 0.026 | 1000 | 26.243 | 0.131 | 0.287 |
| Femur | 0.150 | 1000 | 149.62 | -0.036 | 2.258 |
| Crus | 0.092 | 1000 | 91.697 | -0.057 | 1.009 |
| Foot | 0.026 | 1000 | 26.243 | 0.131 | 0.287 |
|  |  |  |  |  |  |
| Axial total | 10.647 | 1000 | 9064.839 | 0.946 | 2.925 |
| Hind limb total | 0.268 | 1000 | 267.56 | -0.027 | 1.637 |
| Fore limb total | 0.114 | 1000 | 114.29 | 2.848 | 1.324 |
| Whole body | 11.410 | 1000 | 9828.539 | 0.937 | 2.818 |
|  |  |  |  |  |  |
| **Respiratory structures** |  |  |  |  |  |
| Head | 0.0010 | 1000 | 0.988 | 8.407 | 3.217 |
| Neck | 1.3934 | 1000 | 1393.371 | 1.418 | 2.949 |
| Trunk | 0.1187 | 1000 | 118.738 | 4.575 | 2.921 |

**Table S19.** Body segment and whole model mass properties from *Amargasaurus* convex hull model.

| **Body segments** | **Volume** | **Density** | **Mass** | **CoM X** | **CoM Y** |
| --- | --- | --- | --- | --- | --- |
| Head | 0.029 | 1000 | 29.467 | 3.304 | 2.236 |
| Neck | 0.327 | 1000 | 326.774 | 1.991 | 2.542 |
| Trunk | 2.351 | 1000 | 2350.974 | -0.202 | 2.209 |
| Tail | 0.331 | 1000 | 330.981 | -2.681 | 1.863 |
| Humerus | 0.023 | 1000 | 22.945 | 1.01 | 1.349 |
| Forearm | 0.013 | 1000 | 12.972 | 0.985 | 0.785 |
| Hand | 0.017 | 1000 | 17.328 | 1.018 | 0.362 |
| Humerus | 0.023 | 1000 | 22.945 | 1.01 | 1.349 |
| Forearm | 0.013 | 1000 | 12.972 | 0.985 | 0.785 |
| Hand | 0.017 | 1000 | 17.328 | 1.018 | 0.362 |
| Femur | 0.044 | 1000 | 43.596 | -0.969 | 1.534 |
| Crus | 0.029 | 1000 | 28.612 | -0.983 | 0.764 |
| Foot | 0.007 | 1000 | 7.355 | -0.989 | 0.289 |
| Femur | 0.044 | 1000 | 43.596 | -0.969 | 1.534 |
| Crus | 0.029 | 1000 | 28.612 | -0.983 | 0.764 |
| Foot | 0.007 | 1000 | 7.355 | -0.989 | 0.289 |
|  |  |  |  |  |  |
| Axial total | 3.038 | 1000 | 3038.196 | -0.202 | 2.207 |
| Hind limb total | 0.08 | 1000 | 79.563 | -0.976 | 1.142 |
| Fore limb total | 0.053 | 1000 | 53.245 | 1.007 | 0.890 |
| Whole body | 3.304 | 1000 | 3303.812 | -0.201 | 2.114 |
|  |  |  |  |  |  |
| **Respiratory structures** |  |  |  |  |  |
| Head | 0.002 | 1000 | 2.347 | 3.220 | 2.283 |
| Neck | 0.454 | 1000 | 453.538 | 0.173 | 1.89 |
| Trunk | 0.0155 | 1000 | 15.5 | 1.921 | 2.096 |

**Table S20.** Body segment and whole model mass properties from *Dicraeosaurus* convex hull model.

| **Body segments** | **Volume** | **Density** | **Mass** | **CoM X** | **CoM Y** |
| --- | --- | --- | --- | --- | --- |
| Head | 0.028 | 1000 | 28.252 | 4.323 | 2.366 |
| Neck | 0.243 | 1000 | 243.377 | 2.699 | 2.643 |
| Trunk | 3.552 | 1000 | 3552.298 | 0.326 | 2.277 |
| Tail | 0.859 | 1000 | 858.533 | -1.904 | 2.400 |
| Humerus | 0.030 | 1000 | 29.912 | 1.625 | 1.440 |
| Forearm | 0.012 | 1000 | 12.175 | 1.621 | 0.855 |
| Hand | 0.010 | 1000 | 9.837 | 1.669 | 0.392 |
| Humerus | 0.030 | 1000 | 29.912 | 1.625 | 1.440 |
| Forearm | 0.012 | 1000 | 12.175 | 1.621 | 0.855 |
| Hand | 0.010 | 1000 | 9.837 | 1.669 | 0.392 |
| Femur | 0.091 | 1000 | 90.878 | -0.687 | 1.729 |
| Crus | 0.059 | 1000 | 58.502 | -0.704 | 0.691 |
| Foot | 0.009 | 1000 | 9.026 | -0.555 | 0.199 |
| Femur | 0.091 | 1000 | 90.878 | -0.687 | 1.729 |
| Crus | 0.059 | 1000 | 58.502 | -0.704 | 0.691 |
| Foot | 0.009 | 1000 | 9.026 | -0.555 | 0.199 |
|  |  |  |  |  |  |
| Axial total | 4.682 | 1000 | 4682.460 | 0.065 | 2.319 |
| Hind limb total | 0.159 | 1000 | 158.406 | -0.686 | 1.258 |
| Fore limb total | 0.052 | 1000 | 51.924 | 1.632 | 1.104 |
| Whole body | 5.104 | 1000 | 5103.120 | 0.050 | 2.229 |
|  |  |  |  |  |  |
| **Respiratory structures** |  |  |  |  |  |
| Head | 0.002 | 1000 | 1.947 | 4.284 | 2.462 |
| Neck | 0.678 | 1000 | 678.442 | 0.659 | 2.397 |
| Trunk | 0.027 | 1000 | 27.259 | 2.586 | 2.424 |

**Table S21.** Body segment and whole model mass properties from *Camarasaurus* convex hull model.

| **Body segments** | **Volume** | **Density** | **Mass** | **CoM X** | **CoM Y** |
| --- | --- | --- | --- | --- | --- |
| Head | 0.097 | 1000 | 96.898 | 7.320 | 3.308 |
| Neck | 0.957 | 1000 | 957.317 | 4.730 | 3.418 |
| Trunk | 10.597 | 1000 | 10596.536 | 1.486 | 2.636 |
| Tail | 0.775 | 1000 | 774.758 | -2.275 | 2.805 |
| Humerus | 0.068 | 1000 | 68.361 | 3.130 | 1.728 |
| Forearm | 0.032 | 1000 | 31.698 | 3.096 | 0.836 |
| Hand | 0.031 | 1000 | 31.065 | 3.128 | 0.184 |
| Humerus | 0.068 | 1000 | 68.361 | 3.130 | 1.728 |
| Forearm | 0.032 | 1000 | 31.698 | 3.096 | 0.836 |
| Hand | 0.031 | 1000 | 31.065 | 3.128 | 0.184 |
| Femur | 0.222 | 1000 | 221.693 | 0.125 | 0.203 |
| Crus | 0.089 | 1000 | 88.624 | 0.096 | 0.757 |
| Foot | 0.034 | 1000 | 33.877 | 0.323 | 0.100 |
| Femur | 0.222 | 1000 | 221.693 | 0.125 | 0.203 |
| Crus | 0.089 | 1000 | 88.624 | 0.096 | 0.757 |
| Foot | 0.034 | 1000 | 33.877 | 0.323 | 0.100 |
|  |  |  |  |  |  |
| Axial total | 12.426 | 1000 | 12425.509 | 1.547 | 2.712 |
| Hind limb total | 0.345 | 1000 | 344.194 | 0.137 | 0.336 |
| Fore limb total | 0.131 | 1000 | 131.124 | 3.121 | 1.147 |
| Whole body | 13.378 | 1000 | 13376.145 | 1.505 | 2.559 |
|  |  |  |  |  |  |
| **Respiratory structures** |  |  |  |  |  |
| Head | 0.004 | 1000 | 4.037 | 7.277 | 3.401 |
| Neck | 3.346 | 1000 | 3346.439 | 1.756 | 2.768 |
| Trunk | 0.132 | 1000 | 132.017 | 4.628 | 3.326 |

**Table S22.** Body segment and whole model mass properties from *Giraffatitan* convex hull model.

| **Body segments** | **Volume** | **Density** | **Mass** | **CoM X** | **CoM Y** |
| --- | --- | --- | --- | --- | --- |
| Head | 0.059 | 1000 | 59.450 | 11.630 | 4.711 |
| Neck | 2.461 | 1000 | 2461.003 | 5.393 | 4.808 |
| Trunk | 19.851 | 1000 | 19850.916 | 0.107 | 4.237 |
| Tail | 0.775 | 1000 | 774.758 | -4.374 | 4.754 |
| Humerus | 0.299 | 1000 | 298.781 | 1.628 | 2.260 |
| Forearm | 0.161 | 1000 | 160.674 | 1.720 | 0.567 |
| Hand | 0.086 | 1000 | 85.978 | 1.874 | -0.459 |
| Humerus | 0.299 | 1000 | 298.781 | 1.628 | 2.260 |
| Forearm | 0.161 | 1000 | 160.674 | 1.720 | 0.567 |
| Hand | 0.086 | 1000 | 85.978 | 1.874 | -0.459 |
| Femur | 0.294 | 1000 | 294.191 | -1.771 | 3.515 |
| Crus | 0.193 | 1000 | 193.057 | -1.843 | 1.862 |
| Foot | 0.036 | 1000 | 35.694 | -1.715 | 1.009 |
| Femur | 0.294 | 1000 | 294.191 | -1.771 | 3.515 |
| Crus | 0.193 | 1000 | 193.057 | -1.843 | 1.862 |
| Foot | 0.036 | 1000 | 35.694 | -1.715 | 1.009 |
|  |  |  |  |  |  |
| Axial total | 23.146 | 1000 | 23146.127 | 0.549 | 4.316 |
| Hind limb total | 0.523 | 1000 | 522.942 | -1.794 | 2.734 |
| Fore limb total | 0.546 | 1000 | 545.433 | 1.694 | 1.333 |
| Whole body | 25.284 | 1000 | 25282.877 | 0.501 | 4.122 |
|  |  |  |  |  |  |
| **Respiratory structures** |  |  |  |  |  |
| Head | 0.004 | 1000 | 3.600 | 11.561 | 4.823 |
| Neck | 5.000 | 1000 | 5000.385 | 0.504 | 4.500 |
| Trunk | 0.333 | 1000 | 332.544 | 5.324 | 4.579 |

**Table S23.** Body segment and whole model mass properties from *Sauroposeidon* convex hull model.

| **Body segments** | **Volume** | **Density** | **Mass** | **CoM X** | **CoM Y** |
| --- | --- | --- | --- | --- | --- |
| Head | 0.085 | 1000 | 84.790 | 10.074 | 3.490 |
| Neck | 1.715 | 1000 | 1715.182 | 5.172 | 3.483 |
| Trunk | 8.757 | 1000 | 8756.508 | -0.199 | 2.927 |
| Tail | 0.801 | 1000 | 800.895 | -4.262 | 2.939 |
| Humerus | 0.074 | 1000 | 74.442 | 1.464 | 2.126 |
| Forearm | 0.050 | 1000 | 50.240 | 1.493 | 1.157 |
| Hand | 0.022 | 1000 | 21.979 | 1.475 | 0.432 |
| Humerus | 0.074 | 1000 | 74.442 | 1.464 | 2.126 |
| Forearm | 0.050 | 1000 | 50.240 | 1.493 | 1.157 |
| Hand | 0.022 | 1000 | 21.979 | 1.475 | 0.432 |
| Femur | 0.092 | 1000 | 91.919 | -1.873 | 2.166 |
| Crus | 0.054 | 1000 | 53.907 | -1.877 | 1.016 |
| Foot | 0.013 | 1000 | 13.103 | -1.860 | 0.373 |
| Femur | 0.092 | 1000 | 91.919 | -1.873 | 2.166 |
| Crus | 0.054 | 1000 | 53.907 | -1.877 | 1.016 |
| Foot | 0.013 | 1000 | 13.103 | -1.860 | 0.373 |
|  |  |  |  |  |  |
| Axial total | 11.358 | 1000 | 11357.375 | 0.402 | 3.016 |
| Hind limb total | 0.159 | 1000 | 158.929 | -1.873 | 1.628 |
| Fore limb total | 0.146 | 1000 | 146.661 | 1.476 | 1.540 |
| Whole body | 11.968 | 1000 | 11968.555 | 0.370 | 2.943 |
|  |  |  |  |  |  |
| **Respiratory structures** |  |  |  |  |  |
| Head | 0.003 | 1000 | 3.151 | 10.019 | 3.543 |
| Neck | 2.629 | 1000 | 2629.116 | 0.101 | 3.018 |
| Trunk | 0.191 | 1000 | 191.234 | 5.482 | 3.261 |

**Table S24.** Body segment and whole model mass properties from *Dreadnoughtus* convex hull model.

| **Body segments** | **Volume** | **Density** | **Mass** | **CoM X** | **CoM Y** |
| --- | --- | --- | --- | --- | --- |
| Head | 0.033 | 1000 | 33.492 | 13.277 | 4.342 |
| Neck | 3.110 | 1000 | 3109.993 | 6.750 | 4.451 |
| Trunk | 21.346 | 1000 | 21345.839 | 0.097 | 3.735 |
| Tail | 1.011 | 1000 | 1011.349 | -4.515 | 3.981 |
| Humerus | 0.186 | 1000 | 186.076 | 1.858 | 2.270 |
| Forearm | 0.097 | 1000 | 97.356 | 1.806 | 1.016 |
| Hand | 0.024 | 1000 | 24.112 | 1.871 | 0.263 |
| Humerus | 0.186 | 1000 | 186.076 | 1.858 | 2.270 |
| Forearm | 0.097 | 1000 | 97.356 | 1.806 | 1.016 |
| Hand | 0.024 | 1000 | 24.112 | 1.871 | 0.263 |
| Femur | 0.246 | 1000 | 246.125 | -1.936 | 2.933 |
| Crus | 0.110 | 1000 | 109.864 | -1.996 | 1.393 |
| Foot | 0.042 | 1000 | 41.910 | -1.765 | 0.700 |
| Femur | 0.246 | 1000 | 246.125 | 1.936 | 2.933 |
| Crus | 0.110 | 1000 | 109.864 | -1.996 | 1.393 |
| Foot | 0.042 | 1000 | 41.910 | -1.765 | 0.700 |
|  |  |  |  |  |  |
| Axial total | 25.500 | 1000 | 25500.673 | 0.743 | 3.833 |
| Hind limb total | 0.398 | 1000 | 397.899 | -1.935 | 2.273 |
| Fore limb total | 0.307 | 1000 | 307.544 | 1.843 | 1.716 |
| Whole body | 26.910 | 1000 | 26911.559 | 0.724 | 3.738 |
|  |  |  |  |  |  |
| **Respiratory structures** |  |  |  |  |  |
| Head | 0.003 | 1000 | 3.429 | 13.214 | 4.379 |
| Neck | 4.303 | 1000 | 4303.674 | 0.331 | 3.885 |
| Trunk | 0.486 | 1000 | 486.478 | 7.377 | 4.087 |

**Table S25.** Body segment and whole model mass properties from *Rapetosaurus* convex hull model.

| **Body segments** | **Volume** | **Density** | **Mass** | **CoM X** | **CoM Y** |
| --- | --- | --- | --- | --- | --- |
| Head | 0.004 | 1000 | 4.186 | 4.242 | 1.533 |
| Neck | 0.165 | 1000 | 165.353 | 2.286 | 1.569 |
| Trunk | 0.782 | 1000 | 782.444 | 0.052 | 1.379 |
| Tail | 0.020 | 1000 | 20.123 | -1.566 | 1.502 |
| Humerus | 0.008 | 1000 | 8.448 | 0.684 | 0.983 |
| Forearm | 0.004 | 1000 | 3.905 | 0.670 | 0.559 |
| Hand | 0.002 | 1000 | 1.770 | 0.678 | 0.249 |
| Humerus | 0.008 | 1000 | 8.448 | 0.684 | 0.983 |
| Forearm | 0.004 | 1000 | 3.905 | 0.670 | 0.559 |
| Hand | 0.002 | 1000 | 1.770 | 0.678 | 0.249 |
| Femur | 0.012 | 1000 | 12.465 | -0.698 | 0.976 |
| Crus | 0.005 | 1000 | 5.077 | -0.705 | 0.492 |
| Foot | 0.003 | 1000 | 3.312 | -0.668 | 0.125 |
| Femur | 0.012 | 1000 | 12.465 | -0.698 | 0.976 |
| Crus | 0.005 | 1000 | 5.077 | -0.705 | 0.492 |
| Foot | 0.003 | 1000 | 3.312 | -0.668 | 0.125 |
|  |  |  |  |  |  |
| Axial total | 0.971 | 1000 | 972.106 | 0.417 | 1.415 |
| Hind limb total | 0.020 | 1000 | 20.854 | -0.695 | 0.723 |
| Fore limb total | 0.014 | 1000 | 14.123 | 0.679 | 0.774 |
| Whole body | 1.039 | 1000 | 1042.060 | 0.379 | 1.369 |
|  |  |  |  |  |  |
| **Respiratory structures** |  |  |  |  |  |
| Head | 0.001 | 1000 | 0.510 | 4.219 | 1.570 |
| Neck | 0.147 | 1000 | 146.885 | 0.204 | 1.431 |
| Trunk | 0.022 | 1000 | 22.251 | 2.387 | 1.474 |

**Table S26.** Body segment and whole model mass properties from *Neuquensaurus* convex hull model.

| **Body segments** | **Volume** | **Density** | **Mass** | **CoM X** | **CoM Y** |
| --- | --- | --- | --- | --- | --- |
| Head | 0.017 | 1000 | 17.465 | 2.886 | 1.659 |
| Neck | 0.070 | 1000 | 70.273 | 1.789 | 1.680 |
| Trunk | 1.073 | 1000 | 1072.899 | 0.213 | 1.494 |
| Tail | 0.149 | 1000 | 148.745 | -2.360 | 1.626 |
| Humerus | 0.013 | 1000 | 13.216 | 0.898 | 1.059 |
| Forearm | 0.017 | 1000 | 17.411 | 0.914 | 0.522 |
| Hand | - | - | - | - | - |
| Humerus | 0.013 | 1000 | 13.216 | 0.898 | 1.059 |
| Forearm | 0.017 | 1000 | 17.411 | 0.914 | 0.522 |
| Hand | - | - | - | - | - |
| Femur | 0.024 | 1000 | 23.769 | -0.428 | 1.234 |
| Crus | 0.014 | 1000 | 13.940 | -0.422 | 0.648 |
| Foot | 0.009 | 1000 | 9.032 | -0.436 | 0.228 |
| Femur | 0.024 | 1000 | 23.769 | -0.428 | 1.234 |
| Crus | 0.014 | 1000 | 13.940 | -0.422 | 0.648 |
| Foot | 0.009 | 1000 | 9.032 | -0.436 | 0.228 |
|  |  |  |  |  |  |
| Axial total | 1.309 | 1000 | 1309.382 | 0.041 | 1.521 |
| Hind limb total | 0.047 | 1000 | 46.741 | -0.428 | 0.865 |
| Fore limb total | 0.030 | 1000 | 30.627 | 0.907 | 0.754 |
| Whole body | 1.463 | 1000 | 1464.118 | 0.047 | 1.447 |
|  |  |  |  |  |  |
| **Respiratory structures** |  |  |  |  |  |
| Head | 0.001 | 1000 | 0.613 | 2.867 | 1.700 |
| Neck | 0.163 | 1000 | 163.398 | 0.396 | 1.552 |
| Trunk | 0.005 | 1000 | 5.016 | 1.892 | 1.560 |

**Table S27.** Summary of mass properties of different model iterations.

| ***Marasuchus*** |  |  |  |  |  |
| --- | --- | --- | --- | --- | --- |
|  | Volume | Density | Mass | CoM X | CoM Y |
| Minimum Convex Hull | 0.0002 | 903.91 | 0.156 | 0.037 | 0.157 |
| Plus 21% | 0.0002 | 918.91 | 0.188 | 0.036 | 0.158 |
| Maximal | 0.0003 | 942.88 | 0.274 | 0.037 | 0.154 |
| Most Caudal | 0.0002 | 929.86 | 0.220 | 0.023 | 0.153 |
| Most Cranial | 0.0002 | 923.75 | 0.201 | 0.051 | 0.157 |
|  |  |  |  |  |  |
| ***Heterodontosaurus*** |  |  |  |  |  |
|  | Volume | Density | Mass | CoM X | CoM Y |
| Minimum Convex Hull | 0.0026 | 797.09 | 2.037 | 0.076 | 0.312 |
| Plus 21% | 0.0030 | 829.07 | 2.515 | 0.080 | 0.318 |
| Maximal | 0.0043 | 878.22 | 3.739 | 0.076 | 0.309 |
| Most Caudal | 0.0035 | 850.94 | 2.960 | 0.045 | 0.304 |
| Most Cranial | 0.0032 | 837.84 | 2.679 | 0.108 | 0.315 |
|  |  |  |  |  |  |
| ***Staurikosaurus*** |  |  |  |  |  |
|  | Volume | Density | Mass | CoM X | CoM Y |
| Minimum Convex Hull | 0.0146 | 897.72 | 13.065 | 0.087 | 0.577 |
| Plus 21% | 0.0175 | 914.71 | 15.966 | 0.089 | 0.583 |
| Maximal | 0.0248 | 939.91 | 23.287 | 0.081 | 0.573 |
| Most Caudal | 0.0206 | 927.73 | 19.110 | 0.026 | 0.531 |
| Most Cranial | 0.0180 | 917.48 | 16.551 | 0.146 | 0.589 |
|  |  |  |  |  |  |
| ***Coelophysis*** |  |  |  |  |  |
|  | Volume | Density | Mass | CoM X | CoM Y |
| Minimum Convex Hull | 0.0143 | 877.90 | 12.547 | -0.0093 | 0.509 |
| Plus 21% | 0.0173 | 896.23 | 15.528 | -0.0023 | 0.510 |
| Maximal | 0.0244 | 926.46 | 22.650 | -0.0216 | 0.510 |
| Most Caudal | 0.0209 | 913.97 | 19.102 | -0.0900 | 0.509 |
| Most Cranial | 0.0172 | 898.32 | 15.417 | 0.0643 | 0.510 |
|  |  |  |  |  |  |
| ***Plateosaurus*** |  |  |  |  |  |
|  | Volume | Density | Mass | CoM X | CoM Y |
| Minimum Convex Hull | 0.5074 | 766.98 | 389.20 | 0.205 | 1.166 |
| Plus 21% | 0.6113 | 808.61 | 494.33 | 0.234 | 0.936 |
| Maximal | 0.8320 | 858.37 | 714.17 | 0.206 | 1.166 |
| Most Caudal | 0.7057 | 832.43 | 587.43 | 0.085 | 1.162 |
| Most Cranial | 0.6055 | 804.02 | 486.81 | 0.328 | 1.169 |
|  |  |  |  |  |  |
|  |  |  |  |  |  |
|  |  |  |  |  |  |
| Table S27 continued |  |  |  |  |  |
| ***Lufengosaurus*** |  |  |  |  |  |
|  | Volume | Density | Mass | CoM X | CoM Y |
| Minimum Convex Hull | 1.370 | 885.30 | 1213.06 | -0.300 | 0.858 |
| Plus 21% | 1.654 | 904.98 | 1496.86 | -0.285 | 0.864 |
| Maximal | 2.226 | 929.40 | 2068.91 | -0.318 | 0.853 |
| Most Caudal | 1.845 | 914.81 | 1687.75 | -0.459 | 0.843 |
| Most Cranial | 1.669 | 905.84 | 1511.92 | -0.163 | 0.865 |
|  |  |  |  |  |  |
| ***Cetiosaurus*** |  |  |  |  |  |
|  | Volume | Density | Mass | CoM X | CoM Y |
| Minimum Convex Hull | 1.370 | 870.13 | 5083.27 | -0.011 | 2.372 |
| Plus 21% | 1.654 | 892.23 | 6287.53 | 0.022 | 2.392 |
| Maximal | 2.226 | 921.97 | 8988.53 | 0.061 | 2.201 |
| Most Caudal | 1.845 | 904.05 | 7160.09 | -0.142 | 2.223 |
| Most Cranial | 1.669 | 896.84 | 6601.51 | 0.211 | 2.290 |
|  |  |  |  |  |  |
| ***Patagosaurus*** |  |  |  |  |  |
|  | Volume | Density | Mass | CoM X | CoM Y |
| Minimum Convex Hull | 8.5839 | 874.63 | 7507.73 | -0.597 | 2.756 |
| Plus 21% | 10.3572 | 896.10 | 9281.04 | -0.577 | 2.766 |
| Maximal | 14.0409 | 923.36 | 12964.72 | -0.603 | 2.735 |
| Most Caudal | 11.4793 | 906.25 | 10403.17 | -0.884 | 2.741 |
| Most Cranial | 10.6451 | 898.91 | 9568.98 | -0.312 | 2.739 |
|  |  |  |  |  |  |
| ***Mamenchisaurus*** |  |  |  |  |  |
|  | Volume | Density | Mass | CoM X | CoM Y |
| Minimum Convex Hull | 12.063 | 791.057 | 9542.738 | 0.606 | 2.654 |
| Plus 21% | 14.577 | 827.093 | 12056.901 | 0.660 | 2.659 |
| Maximal | 19.644 | 871.691 | 17123.730 | 0.915 | 2.666 |
| Most Caudal | 14.773 | 829.386 | 12252.819 | 0.256 | 2.656 |
| Most Cranial | 16.232 | 844.714 | 13711.055 | 1.331 | 2.666 |
|  |  |  |  |  |  |
| ***Jobaria*** |  |  |  |  |  |
|  | Volume | Density | Mass | CoM X | CoM Y |
| Minimum Convex Hull | 13.173 | 859.40 | 11320.99 | -0.098 | 3.485 |
| Plus 21% | 15.898 | 883.47 | 14045.05 | -0.082 | 3.493 |
| Maximal | 21.661 | 914.54 | 19809.63 | -0.016 | 3.481 |
| Most Caudal | 16.780 | 889.65 | 14928.74 | -0.398 | 3.427 |
| Most Cranial | 17.304 | 892.99 | 15452.52 | 0.307 | 3.523 |
|  |  |  |  |  |  |
|  |  |  |  |  |  |
|  |  |  |  |  |  |
| Table S27 continued |  |  |  |  |  |
| ***Apatosaurus*** |  |  |  |  |  |
|  | Volume | Density | Mass | CoM X | CoM Y |
| Minimum Convex Hull | 26.628 | 806.405 | 21473.151 | -0.087 | 3.934 |
| Plus 21% | 32.259 | 838.005 | 27033.500 | -0.051 | 3.940 |
| Maximal | 43.304 | 879.322 | 38078.331 | 0.024 | 3.917 |
| Most Caudal | 34.087 | 846.728 | 28862.680 | -0.465 | 3.885 |
| Most Cranial | 34.300 | 847.638 | 29073.789 | 0.431 | 3.957 |
|  |  |  |  |  |  |
| ***Barosaurus*** |  |  |  |  |  |
|  | Volume | Density | Mass | CoM X | CoM Y |
| Minimum Convex Hull | 16.673 | 869.14 | 14491.10 | 0.783 | 3.329 |
| Plus 21% | 20.134 | 891.62 | 17951.43 | 0.829 | 3.331 |
| Maximal | 28.257 | 922.83 | 26075.98 | 1.045 | 3.345 |
| Most Caudal | 21.014 | 896.22 | 18832.84 | 0.198 | 3.279 |
| Most Cranial | 23.102 | 905.56 | 20920.35 | 1.663 | 3.392 |
|  |  |  |  |  |  |
| ***Diplodocus*** |  |  |  |  |  |
|  | Volume | Density | Mass | CoM X | CoM Y |
| Minimum Convex Hull | 11.410 | 718.70 | 8200.54 | 0.767 | 2.788 |
| Plus 21% | 11.875 | 862.94 | 10247.36 | 0.801 | 2.783 |
| Maximal | 16.183 | 899.40 | 14555.11 | 0.908 | 2.629 |
| Most Caudal | 13.124 | 876.00 | 11496.58 | 0.536 | 2.765 |
| Most Cranial | 12.331 | 867.97 | 10703.23 | 1.186 | 2.778 |
|  |  |  |  |  |  |
| ***Amargasaurus*** |  |  |  |  |  |
|  | Volume | Density | Mass | CoM X | CoM Y |
| Minimum Convex Hull | 3.304 | 848.55 | 2803.60 | -0.287 | 2.148 |
| Plus 21% | 3.988 | 874.38 | 3487.03 | -0.272 | 1.995 |
| Maximal | 5.432 | 907.92 | 4931.92 | -0.250 | 1.968 |
| Most Caudal | 4.287 | 883.42 | 3787.41 | -0.515 | 1.948 |
| Most Cranial | 4.261 | 882.33 | 3760.03 | -0.013 | 2.012 |
|  |  |  |  |  |  |
| ***Dicraeosaurus*** |  |  |  |  |  |
|  | Volume | Density | Mass | CoM X | CoM Y |
| Minimum Convex Hull | 5.104 | 857.19 | 4375.10 | -0.071 | 2.199 |
| Plus 21% | 6.028 | 878.99 | 5298.56 | -0.022 | 2.199 |
| Maximal | 10.206 | 928.67 | 9478.22 | 0.114 | 2.082 |
| Most Caudal | 6.884 | 894.31 | 6156.15 | -0.156 | 2.013 |
| Most Cranial | 6.085 | 880.17 | 5356.28 | 0.192 | 2.200 |
|  |  |  |  |  |  |
|  |  |  |  |  |  |
|  |  |  |  |  |  |
| Table S27 continued |  |  |  |  |  |
| ***Camarasaurus*** |  |  |  |  |  |
|  | Volume | Density | Mass | CoM X | CoM Y |
| Minimum Convex Hull | 13.378 | 731.03 | 9779.78 | 1.762 | 2.401 |
| Plus 21% | 15.987 | 776.32 | 12410.96 | 1.436 | 2.477 |
| Maximal | 21.454 | 832.19 | 17853.85 | 1.440 | 2.545 |
| Most Caudal | 17.064 | 788.87 | 13461.51 | 1.124 | 2.518 |
| Most Cranial | 16.920 | 787.44 | 13323.44 | 1.710 | 2.533 |
|  |  |  |  |  |  |
| ***Giraffatitan*** |  |  |  |  |  |
|  | Volume | Density | Mass | CoM X | CoM Y |
| Minimum Convex Hull | 25.284 | 779.81 | 19716.77 | 0.367 | 4.010 |
| Plus 21% | 30.542 | 817.72 | 24975.07 | 0.394 | 4.048 |
| Maximal | 40.399 | 862.18 | 34830.84 | 0.534 | 4.067 |
| Most Caudal | 31.202 | 821.57 | 25634.71 | 0.099 | 4.032 |
| Most Cranial | 32.893 | 830.72 | 27324.82 | 0.846 | 4.043 |
|  |  |  |  |  |  |
| ***Sauroposeidon*** |  |  |  |  |  |
|  | Volume | Density | Mass | CoM X | CoM Y |
| Minimum Convex Hull | 11.968 | 753.81 | 9021.65 | 0.102 | 3.315 |
| Plus 21% | 14.467 | 796.34 | 11520.32 | 0.127 | 3.315 |
| Maximal | 19.559 | 849.33 | 16611.96 | 0.264 | 3.421 |
| Most Caudal | 14.925 | 802.61 | 11979.27 | -0.288 | 3.186 |
| Most Cranial | 15.902 | 814.63 | 12953.81 | 0.688 | 3.591 |
|  |  |  |  |  |  |
| ***Dreadnoughtus*** |  |  |  |  |  |
|  | Volume | Density | Mass | CoM X | CoM Y |
| Minimum Convex Hull | 26.910 | 813.69 | 21896.69 | 0.603 | 3.695 |
| Plus 21% | 32.534 | 845.90 | 27520.39 | 0.629 | 3.707 |
| Maximal | 43.016 | 883.47 | 38003.29 | 0.835 | 3.726 |
| Most Caudal | 33.116 | 848.61 | 28102.65 | 0.307 | 3.681 |
| Most Cranial | 35.102 | 857.19 | 30089.67 | 1.200 | 3.745 |
|  |  |  |  |  |  |
| ***Rapetosaurus*** |  |  |  |  |  |
|  | Volume | Density | Mass | CoM X | CoM Y |
| Minimum Convex Hull | 1.039 | 816.87 | 848.72 | 0.332 | 1.350 |
| Plus 21% | 1.255 | 848.88 | 1065.42 | 0.343 | 1.356 |
| Maximal | 1.677 | 888.13 | 1489.40 | 0.442 | 1.364 |
| Most Caudal | 1.257 | 849.69 | 1068.24 | 0.219 | 1.340 |
| Most Cranial | 1.396 | 864.68 | 1207.28 | 0.582 | 1.374 |
|  |  |  |  |  |  |
|  |  |  |  |  |  |
|  |  |  |  |  |  |
| Table S27 continued |  |  |  |  |  |
| ***Neuquensaurus*** |  |  |  |  |  |
|  | Volume | Density | Mass | CoM X | CoM Y |
| Minimum Convex Hull | 1.463 | 877.64 | 1283.76 | -0.016 | 1.576 |
| Plus 21% | 1.768 | 897.88 | 1587.43 | 0.002 | 1.546 |
| Maximal | 2.392 | 924.59 | 2211.43 | -0.024 | 1.497 |
| Most Caudal | 1.929 | 907.66 | 1751.30 | -0.201 | 1.519 |
| Most Cranial | 1.839 | 901.74 | 1658.06 | 0.156 | 1.537 |

**Table S28.** CoM distance in front of hip for Plus 21%, Max Caudal and Max Cranial models.

|  | **CoM distance in front of hip (m)** | | |
| --- | --- | --- | --- |
|  | Plus21 | Most Caudal | Most Cranial |
| *Alligator* | 0.139 | 0.109 | 0.208 |
| *Marasuchus* | 0.040 | 0.028 | 0.054 |
| *Heterodontosaurus* | 0.091 | 0.060 | 0.114 |
| *Staurikosaurus* | 0.102 | 0.041 | 0.154 |
| *Coelophysis* | 0.037 | -0.049 | 0.097 |
| *Plateosaurus* | 0.211 | 0.062 | 0.306 |
| *Lufengosaurus* | 0.435 | 0.261 | 0.558 |
| *Cetiosaurus* | 0.610 | 0.446 | 0.799 |
| *Patagosaurus* | 0.778 | 0.471 | 1.043 |
| *Mamenchisaurus* | 2.323 | 1.920 | 2.994 |
| *Jobaria* | 1.261 | 0.945 | 1.650 |
| *Apatosaurus* | 1.918 | 1.503 | 2.399 |
| *Barosaurus* | 1.707 | 1.076 | 2.542 |
| *Diplodocus* | 0.833 | 0.568 | 1.219 |
| *Amargasaurus* | 0.681 | 0.438 | 0.939 |
| *Dicraeosaurus* | 0.657 | 0.522 | 0.870 |
| *Camarasaurus* | 1.380 | 1.010 | 1.595 |
| *Giraffatitan* | 2.117 | 1.822 | 2.569 |
| *Sauroposeidon* | 2.186 | 1.729 | 2.849 |
| *Dreadnoughtus* | 2.547 | 2.226 | 3.119 |
| *Rapetosaurus* | 1.042 | 0.918 | 1.281 |
| *Neuquensaurus* | 0.452 | 0.249 | 0.606 |

**Table S29.** CoM distance in front of hip for Plus 21%, Max Caudal and Max Cranial models (data from Table S27) normalized by mean body mass^0.33^.

|  |  | **CoM distance in front of hips (body mass^0.33^)** | | |
| --- | --- | --- | --- | --- |
|  | **Mean Mass (kg)** | Plus21 | Most Caudal | Most Cranial |
| *Alligator* | 8.812 | 0.068 | 0.053 | 0.102 |
| *Marasuchus* | 0.225 | 0.066 | 0.045 | 0.088 |
| *Heterodontosaurus* | 3.304 | 0.062 | 0.040 | 0.077 |
| *Staurikosaurus* | 19.085 | 0.038 | 0.015 | 0.058 |
| *Coelophysis* | 18.847 | 0.014 | -0.018 | 0.037 |
| *Plateosaurus* | 534.386 | 0.027 | 0.008 | 0.038 |
| *Lufengosaurus* | 1595.700 | 0.038 | 0.023 | 0.049 |
| *Cetiosaurus* | 6824.187 | 0.033 | 0.024 | 0.043 |
| *Patagosaurus* | 9945.128 | 0.037 | 0.023 | 0.050 |
| *Mamenchisaurus* | 12937.449 | 0.102 | 0.084 | 0.132 |
| *Jobaria* | 15111.384 | 0.053 | 0.039 | 0.069 |
| *Apatosaurus* | 28904.290 | 0.065 | 0.051 | 0.081 |
| *Barosaurus* | 19654.342 | 0.065 | 0.041 | 0.097 |
| *Diplodocus* | 11040.565 | 0.039 | 0.026 | 0.056 |
| *Amargasaurus* | 3753.999 | 0.045 | 0.029 | 0.062 |
| *Dicraeosaurus* | 6132.864 | 0.037 | 0.029 | 0.049 |
| *Camarasaurus* | 13481.537 | 0.060 | 0.044 | 0.069 |
| *Giraffatitan* | 26496.443 | 0.073 | 0.063 | 0.089 |
| *Sauroposeidon* | 12417.403 | 0.097 | 0.077 | 0.127 |
| *Dreadnoughtus* | 29343.822 | 0.070 | 0.061 | 0.086 |
| *Rapetosaurus* | 1159.504 | 0.102 | 0.089 | 0.125 |
| *Neuquensaurus* | 1698.397 | 0.039 | 0.021 | 0.052 |

**Table S30.** Average proportion of body mass of each major body segment for each taxon modeled.

|  | Head | Neck | Thoracic | Tail | Humerus | Forearm | Femur | Crus | Forelimb | Hindlimb |
| --- | --- | --- | --- | --- | --- | --- | --- | --- | --- | --- |
| *Alligator* | 0.190 | 0.020 | 0.561 | 0.104 | 0.004 | 0.002 | 0.017 | 0.018 | 0.008 | 0.057 |
| *Marasuchus* | 0.055 | 0.018 | 0.605 | 0.130 | 0.006 | 0.012 | 0.019 | 0.019 | 0.030 | 0.074 |
| *Heterodontosaurus* | 0.090 | 0.011 | 0.639 | 0.139 | 0.005 | 0.003 | 0.011 | 0.021 | 0.010 | 0.010 |
| *Staurikosaurus* | 0.061 | 0.040 | 0.562 | 0.170 | 0.004 | 0.002 | 0.018 | 0.025 | 0.010 | 0.076 |
| *Coelophysis* | 0.044 | 0.029 | 0.557 | 0.302 | 0.001 | 0.002 | 0.008 | 0.011 | 0.004 | 0.029 |
| *Plateosaurus* | 0.009 | 0.016 | 0.690 | 0.178 | 0.005 | 0.002 | 0.016 | 0.016 | 0.010 | 0.047 |
| *Lufengosaurus* | 0.006 | 0.044 | 0.726 | 0.129 | 0.003 | 0.004 | 0.019 | 0.014 | 0.008 | 0.039 |
| *Cetiosaurus* | 0.004 | 0.073 | 0.632 | 0.133 | 0.013 | 0.009 | 0.029 | 0.017 | 0.027 | 0.050 |
| *Patagosaurus* | 0.010 | 0.046 | 0.702 | 0.146 | 0.008 | 0.005 | 0.014 | 0.011 | 0.020 | 0.027 |
| *Mamenchisaurus* | 0.007 | 0.188 | 0.703 | 0.057 | 0.005 | 0.002 | 0.008 | 0.003 | 0.008 | 0.013 |
| *Jobaria* | 0.015 | 0.105 | 0.683 | 0.065 | 0.013 | 0.013 | 0.013 | 0.013 | 0.029 | 0.029 |
| *Apatosaurus* | 0.001 | 0.108 | 0.731 | 0.077 | 0.010 | 0.004 | 0.014 | 0.009 | 0.015 | 0.026 |
| *Barosaurus* | 0.005 | 0.231 | 0.576 | 0.098 | 0.010 | 0.008 | 0.011 | 0.006 | 0.020 | 0.022 |
| *Diplodocus* | 0.002 | 0.070 | 0.543 | 0.115 | 0.006 | 0.003 | 0.013 | 0.008 | 0.010 | 0.024 |
| *Amargasaurus* | 0.010 | 0.108 | 0.683 | 0.109 | 0.008 | 0.004 | 0.014 | 0.009 | 0.017 | 0.026 |
| *Dicraeosaurus* | 0.006 | 0.050 | 0.720 | 0.176 | 0.006 | 0.002 | 0.019 | 0.012 | 0.011 | 0.032 |
| *Camarasaurus* | 0.008 | 0.079 | 0.772 | 0.061 | 0.006 | 0.003 | 0.018 | 0.007 | 0.011 | 0.028 |
| *Giraffatitan* | 0.003 | 0.108 | 0.766 | 0.034 | 0.013 | 0.007 | 0.013 | 0.008 | 0.023 | 0.022 |
| *Sauroposeidon* | 0.008 | 0.157 | 0.705 | 0.073 | 0.007 | 0.005 | 0.008 | 0.005 | 0.013 | 0.014 |
| *Dreadnoughtus* | 0.001 | 0.128 | 0.773 | 0.042 | 0.008 | 0.004 | 0.010 | 0.005 | 0.012 | 0.016 |
| *Rapetosaurus* | 0.004 | 0.175 | 0.728 | 0.021 | 0.009 | 0.004 | 0.013 | 0.005 | 0.014 | 0.021 |
| *Neuquensaurus* | 0.013 | 0.052 | 0.706 | 0.111 | 0.010 | 0.013 | 0.018 | 0.010 | 0.023 | 0.035 |

**Table S31.** Distance of individual body segment CoM positions relative to the hip (meters; positive distances indicate cranial to hip, negative distances are caudal to the hip).

|  | Head | Neck | Thoracic | Tail | Humerus | Forearm | Femur | Crus | Forelimb | Hindlimb |
| --- | --- | --- | --- | --- | --- | --- | --- | --- | --- | --- |
| *Alligator* | 0.116 | 0.201 | 0.083 | -0.094 | 0.167 | 0.169 | 0.004 | 0.007 | 0.167 | 0.005 |
| *Marasuchus* | 0.296 | 0.247 | 0.087 | -0.152 | 0.196 | 0.196 | -0.002 | 0.000 | 0.199 | 0.006 |
| *Heterodontosaurus* | 0.248 | 0.193 | 0.076 | -0.126 | 0.163 | 0.162 | 0.001 | 0.000 | 0.162 | 0.001 |
| *Staurikosaurus* | 0.277 | 0.187 | 0.063 | 0.158 | 0.131 | 0.133 | 0.000 | 0.000 | 0.133 | 0.005 |
| *Coelophysis* | 0.330 | 0.231 | 0.072 | 0.176 | 0.162 | 0.162 | 0.000 | -0.001 | 0.161 | 0.001 |
| *Plateosaurus* | 0.288 | 0.206 | 0.067 | -0.111 | 0.140 | 0.141 | -0.001 | 0.000 | 0.141 | 0.001 |
| *Lufengosaurus* | 0.297 | 0.206 | 0.059 | -0.113 | 0.108 | 0.108 | -0.001 | -0.005 | 0.108 | 0.003 |
| *Cetiosaurus* | 0.338 | 0.187 | 0.050 | -0.105 | 0.111 | 0.111 | -0.001 | -0.003 | 0.112 | 0.002 |
| *Patagosaurus* | 0.292 | 0.193 | 0.052 | -0.091 | 0.122 | 0.122 | -0.001 | -0.004 | 0.123 | 0.002 |
| *Mamenchisaurus* | 0.501 | 0.291 | 0.073 | -0.092 | 0.156 | 0.155 | 0.000 | 0.000 | 0.156 | 0.001 |
| *Jobaria* | 0.289 | 0.182 | 0.046 | -0.112 | 0.116 | 0.114 | 0.000 | -0.001 | 0.115 | 0.000 |
| *Apatosaurus* | 0.326 | 0.217 | 0.061 | -0.091 | 0.136 | 0.139 | 0.003 | 0.005 | 0.137 | 0.004 |
| *Barosaurus* | 0.480 | 0.247 | 0.039 | -0.148 | 0.105 | 0.105 | 0.003 | 0.004 | 0.105 | 0.004 |
| *Diplodocus* | 0.351 | 0.201 | 0.049 | -0.113 | 0.118 | 0.118 | 0.000 | -0.001 | 0.118 | 0.000 |
| *Amargasaurus* | 0.270 | 0.187 | 0.048 | -0.110 | 0.125 | 0.123 | -0.001 | -0.002 | 0.124 | 0.001 |
| *Dicraeosaurus* | 0.271 | 0.183 | 0.054 | -0.066 | 0.125 | 0.125 | 0.000 | -0.001 | 0.125 | 0.000 |
| *Camarasaurus* | 0.290 | 0.186 | 0.055 | -0.096 | 0.121 | 0.120 | 0.000 | -0.001 | 0.121 | 0.001 |
| *Giraffatitan* | 0.435 | 0.232 | 0.060 | -0.086 | 0.109 | 0.112 | -0.002 | -0.004 | 0.111 | 0.002 |
| *Sauroposeidon* | 0.580 | 0.293 | 0.070 | -0.099 | 0.139 | 0.140 | 0.000 | 0.000 | 0.139 | 0.000 |
| *Dreadnoughtus* | 0.485 | 0.277 | 0.064 | -0.083 | 0.121 | 0.119 | -0.001 | -0.002 | 0.120 | 0.001 |
| *Rapetosaurus* | 0.460 | 0.278 | 0.070 | -0.081 | 0.129 | 0.128 | 0.000 | -0.001 | 0.128 | 0.000 |
| *Neuquensaurus* | 0.277 | 0.186 | 0.055 | -0.159 | 0.112 | 0.113 | 0.002 | 0.002 | 0.113 | 0.002 |

**Table S32.** Estimated first mass moments multiplied by CoM position of individual modeled segments for each taxa (kg m). Values are defined about the hip, and so positive values represent a cranial moment and negative values a caudal moment.

|  | Head | Neck | Thoracic | Tail | Humerus | Forearm | Femur | Crus | Forelimb | Hindlimb |
| --- | --- | --- | --- | --- | --- | --- | --- | --- | --- | --- |
| *Alligator* | 0.0218 | 0.0041 | 0.0464 | -0.0098 | 0.0006 | 0.0004 | 6.48083E-05 | 0.000118232 | 0.001403802 | 0.000297232 |
| *Marasuchus* | 0.0163 | 0.0044 | 0.0526 | -0.0200 | 0.0012 | 0.0023 | -3.60141E-05 | -4.31154E-06 | 0.005924175 | 0.000458955 |
| *Heterodontosaurus* | 0.0222 | 0.0021 | 0.0486 | -0.0174 | 0.0008 | 0.0004 | 9.42252E-06 | -1.0345E-05 | -3.6436E-05 | 0.00158073 |
| *Staurikosaurus* | 0.0167 | 0.0075 | 0.0350 | 0.0266 | 0.0006 | 0.0003 | 5.404E-06 | -1.94168E-06 | 0.000427234 | 0.001332197 |
| *Coelophysis* | 0.0143 | 0.0066 | 0.0398 | 0.0526 | 0.0002 | 0.0002 | 1.9386E-07 | -1.58632E-05 | 0.000642329 | 1.98492E-05 |
| *Plateosaurus* | 0.0026 | 0.0031 | 0.0452 | -0.0194 | 0.0008 | 0.0003 | -1.93273E-05 | 6.25281E-06 | 0.001410943 | 3.48765E-05 |
| *Lufengosaurus* | 0.0016 | 0.0088 | 0.0416 | -0.0142 | 0.0003 | 0.0004 | -2.34407E-05 | -6.40416E-05 | 0.00088546 | 0.000120458 |
| *Cetiosaurus* | 0.0012 | 0.0132 | 0.0309 | -0.0135 | 0.0014 | 0.0010 | -2.56183E-05 | -5.26238E-05 | 0.002906338 | 7.39726E-05 |
| *Patagosaurus* | 0.0028 | 0.0085 | 0.0355 | -0.0129 | 0.0010 | 0.0006 | -1.82716E-05 | -4.02251E-05 | 0.00235476 | 6.43449E-05 |
| *Mamenchisaurus* | 0.0034 | 0.0531 | 0.0499 | -0.0051 | 0.0008 | 0.0004 | -3.58284E-06 | -9.84604E-07 | 0.001245908 | 1.59086E-05 |
| *Jobaria* | 0.0043 | 0.0184 | 0.0302 | -0.0071 | 0.0014 | 0.0014 | 1.91905E-06 | -1.0209E-05 | 0.003270143 | 1.28561E-05 |
| *Apatosaurus* | 0.0003 | 0.0225 | 0.0433 | -0.0067 | 0.0013 | 0.0006 | 3.53139E-05 | 4.2118E-05 | 0.001962616 | 0.000112443 |
| *Barosaurus* | 0.0025 | 0.0551 | 0.0215 | -0.0141 | 0.0010 | 0.0009 | 3.05874E-05 | 2.40624E-05 | 0.002022339 | 8.2659E-05 |
| *Diplodocus* | 0.0008 | 0.0136 | 0.0260 | -0.0126 | 0.0007 | 0.0003 | -1.76653E-06 | -7.8781E-06 | 0.001154641 | 5.48443E-06 |
| *Amargasaurus* | 0.0026 | 0.0196 | 0.0317 | -0.0116 | 0.0009 | 0.0005 | -1.46026E-05 | -1.77384E-05 | 0.002089078 | 3.76249E-05 |
| *Dicraeosaurus* | 0.0015 | 0.0088 | 0.0381 | -0.0113 | 0.0007 | 0.0003 | -8.24268E-06 | -1.60117E-05 | 0.001279459 | 1.22132E-05 |
| *Camarasaurus* | 0.0021 | 0.0142 | 0.0412 | -0.0057 | 0.0007 | 0.0003 | 7.34198E-06 | -5.33942E-06 | 0.001263065 | 2.45985E-05 |
| *Giraffatitan* | 0.0011 | 0.0241 | 0.0441 | -0.0028 | 0.0014 | 0.0008 | -1.95658E-05 | -3.19902E-05 | 0.002451118 | 5.0172E-05 |
| *Sauroposeidon* | 0.0043 | 0.0444 | 0.0475 | -0.0070 | 0.0009 | 0.0006 | 9.19595E-07 | -2.52918E-07 | 0.001792312 | 1.41774E-06 |
| *Dreadnoughtus* | 0.0006 | 0.0342 | 0.0480 | -0.0033 | 0.0009 | 0.0005 | -5.50527E-06 | -1.08108E-05 | 0.0014327 | 7.92437E-06 |
| *Rapetosaurus* | 0.0020 | 0.0474 | 0.0497 | -0.0017 | 0.0011 | 0.0005 | 1.46391E-06 | -2.81647E-06 | 0.001802306 | 8.18616E-06 |
| *Neuquensaurus* | 0.0035 | 0.0095 | 0.0379 | -0.0172 | 0.0011 | 0.0014 | 3.17496E-05 | 2.36833E-05 | 0.00251616 | 6.25149E-05 |

**Table S33. Pagel’s Lambda evolutionary signal test results for all segment masses variables with p < 0.05.**

| **Variable** | **Pagel's Lambda** | **Pagel's Lambda p** |
| --- | --- | --- |
| Pectoral Limb FMM | 0.9999 | <0.000001 |
| Mean Head Mass | 0.9999 | <0.000001 |
| Head FMM | 0.9999 | <0.000001 |
| Pelvic Limb length | 0.9512 | <0.000001 |
| Pectoral Limb Length | 0.9214 | <0.000001 |
| Pectoral Limb CoM | 0.9076 | <0.000001 |
| Max Caudal CoM | 0.8881 | 0.0163 |
| Mean Pectoral Limb Mass | 0.8730 | 0.0019 |
| Mean CoM | 0.8637 | 0.0483 |
| Thoracic CoM | 0.8288 | 0.0000 |
| GA Length | 0.7962 | 0.0000 |
| Pelvic Limb FMM | 0.7461 | 0.0003 |
| Pelvic Limb CoM | 0.7058 | 0.0022 |
| Mean Tail Mass | 0.6002 | 0.0271 |
| Neck Length | 0.5928 | 0.0002 |
| Thoracic FMM | 0.5911 | 0.0499 |
| Mean Pelvic Limb Mass | 0.5640 | 0.0004 |
| Tail CoM | 0.5623 | 0.0026 |
| Mean Thoracic Mass | 0.5621 | 0.0018 |
| Tail FMM | 0.5467 | 0.0263 |
| Mean Neck Mass | 0.5310 | 0.0006 |
| Neck FMM | 0.4346 | 0.0093 |

**Table S34. Spearman’s Rank statistics for body segment parameters in PIC analysis with p = <0.05.**

| **Segment Parameter** | **Spearmans Rank** | **Spearmans Rank p** |
| --- | --- | --- |
| Neck FMM | 0.977 | <0.0005 |
| Neck CoM | 0.973 | <0.0005 |
| Mean Neck Mass | 0.935 | <0.0005 |
| Head CoM | 0.932 | <0.0005 |
| Thoracic FMM | 0.858 | <0.0005 |
| Mean whole Body Mass | 0.831 | <0.0005 |
| Mean Thoracic Mass | 0.804 | <0.0005 |
| Neck Length | 0.801 | <0.0005 |
| Thoracic CoM | 0.773 | <0.0005 |
| Pectoral Limb CoM | 0.769 | <0.0005 |
| Pectoral Limb FMM | 0.694 | 0.0005 |
| Pectoral Limb Length | 0.678 | 0.0007 |
| Mean Pectoral Limb Mass | 0.675 | 0.0008 |
| GA Length | 0.658 | 0.0012 |
| Mean Pelvic Limb Mass | 0.600 | 0.0040 |
| Head FMM | 0.522 | 0.0152 |
| Pelvic Limb length | 0.497 | 0.0218 |
| Tail CoM | -0.440 | 0.0458 |
| Tail FMM | -0.464 | 0.0343 |

**Table S35. Results of sensitivity analysis of respiratory volumes in the *Giraffatitan* model with CoM positions normalised by distance in front of the hip/body mass^0.33^.**

| **Model iteration** | **Original Model** | **Smaller zero-density structures (absolute)** | **Smaller zero-density structures (%change)** | **Larger zero-density structures (absolute)** | **Larger zero-density structures (%change)** |
| --- | --- | --- | --- | --- | --- |
| Plus 21% | 0.07346 | 0.07258 | 1.20 | 0.07376 | -0.42 |
| Most Caudal | 0.06321 | 0.06329 | -0.12 | 0.06200 | 1.93 |
| Most cranial | 0.08913 | 0.08675 | 2.67 | 0.09196 | -3.17 |

**Table S36. Results of sensitivity analysis of respiratory volumes in the *Giraffatitan* model with CoM positions normalised by gleno-acetabular distance.**

| **Model iteration** | **Original Model** | **Smaller zero-density structures (absolute)** | **Smaller zero-density structures (%change)** | **Larger zero-density structures (absolute)** | **Larger zero-density structures (%change)** |
| --- | --- | --- | --- | --- | --- |
| Plus 21% | 0.668 | 0.676 | 1.22 | 0.647 | -3.12 |
| Most Caudal | 0.575 | 0.589 | 2.52 | 0.544 | -5.58 |
| Most cranial | 0.810 | 0.808 | -0.28 | 0.807 | -0.36 |

**Full Reference List**

1. Wilson JA. Sauropod dinosaur phylogeny: critique and cladistics analysis. *Zool. J. Linn. Soc.* 2002; 136, 217-276.
2. Upchurch P, Barrett PM, Dodson P. Sauropoda. In: Weishampel DB, Dodson P, Osmólska H, editors. The Dinosauria (2^nd^ Ed.). Berkeley: University of California Press; 2004, pp. 259–324.
3. Sander PM, Christian A, Clauss M, Fechner R, Gee CT, Griebeler EM, *et al.* Biology of the sauropod dinosaurs: the evolution of gigantism. *Biol. Rev.* 2010; 86, 117-160.
4. Benson RBJ, Campione NE, Carrano MT, Mannion PD, Sullivan C, Upchurch P, Evans, DC. Rates of dinosaur body mass evolution indicate 170 million years of sustained ecological innovation on the avian stem lineage. *PLoS Biology* 2014; **12** e1001853. DOI: 10.1371/journal.pbio.1001853.
5. Henderson DM. Burly gaits: centers of mass, stability, and the trackways of sauropod dinosaurs. *J. Verte. Pal*. 2006; 4, 907-921.
6. Henderson DM. Tipsy punters: sauropod dinosaur pneumaticity, buoyancy and aquatic habits. *Proc. Roy. Soc. B* 2004; 271, S180-183.
7. Rauhut, O, Fechner, R, Remes, K, Reis, K. How to get big in the Mesozoic: the evolution of the sauropodomorph body plan. In Klein N, Remes K, Gee CT, Sander PM, editors. Biology of the sauropod dinosaurs: Understanding the life of giants. Bloomington: Indiana University Press; 2011. pp. 119–149.
8. Bell MA, Lloyd GT. Strap: an R package for plotting phylogenies against stratigraphy and assessing their stratigraphic congruence. *Palaeontology*. 2014; 58, 379–389.
9. Sellers WI, Hepworth-Bell J, Falkingham PL, Bates KT, Brassey CA, Egerton V, Manning PL. Minimum convex hull mass estimations of complete mounted skeletons. *Biology* *Letters* 2012; doi: 10.1098/rsbl.2012.0263.
10. Bates KT, Falkingham PL, Macaulay S, Brassey CA, Maidment SCR. Downsizing a gaint: re-evaluating *Dreadnoughtus* body mass. *Biology Letters* 2015; DOI: 10.1098/rsbl.2015.0215.
11. Bates KT, Manning PL, Hodgetts D, Sellers WI. Estimating the body mass of dinosaurs using laser imaging and 3D computer modeling. *PLoS ONE* 2009; 4(2), e4532. doi: 10.1371.
12. Bates KT, Falkingham PL, Breithaupt BH, Hodgetts D, Sellers WI, Manning PL. How big was ‘Big Al’? Quantifying the effect of soft tissue and osteological unknowns on mass predictions for *Allosaurus* (Dinosauria: Theropoda). *Palaeontological Electronica* 2009; 12(3): 14A, p. 33 (2009).
13. Allen V, Bates KT, Zhiheng L, Hutchinson JR. Linking the evolution of body shape and locomotor biomechanics in bird-line archosaurs. *Nature* 2013; 497, 104-107.
14. Allen V, Paxton H, Hutchinson JR. 2009. Variation in center of mass estimates for extant sauropsids and its importance for reconstructing inertial properties in extinct archosaurs. *Anatomical Record* 292, 1442-1461.
15. Yates AM, Kitching JW. The earliest known sauropod dinosaur and the first steps towards sauropod locomotion. *Proc. Roy. Soc. B* 2003; 270, 1753-1758.
16. Falkingham PL. Acquisition of high resolution three-dimensional models using free, open-source, photogrammetric software*. Palaeontologia Electronica* 2012; 15, 1; 1T:15p.
17. Stevens KA. Articulation of sauropod necks: Methodology and mythology. *PLoS ONE* 2013; 8(10): e78572. doi:10.1371/journal.pone.0078572.
18. Stevens KA, Parrish JM. Neck posture and feeding habits of two Jurassic sauropods. *Science* 1999; 284, 798-800.
19. Taylor MP, Hone DWE, Wedel MJ, Naish D. The long necks of sauropods did not evolve primarily through sexual selection. *J. Zool.* 2011; doi:10.1111/j.1469-7998.2011.00824.x.
20. Taylor MP, Wedel MJ. The effect of intervertebral cartilage on neutral posture and range of motion in the necks of sauropod dinosaurs. *PLoS ONE* 2013; 8(10), e78214. doi:10.1371/journal.pone.0078214.
21. Christian A, Dzemski G. 2011 Neck posture in sauropods. In *Biology of the sauropod dinosaurs: understanding the life of giants* (eds Klein N, Remes K, Gee CT, Sander PM), pp. 251–260. Bloomington, IN: Indiana University Press.
22. Wedel MJ. Postcranial Skeletal Pneumaticity in Sauropods and Its Implications for Mass Estimates. In: Wilson JA, Curry-Rogers KA, editors. The sauropods: evolution and paleobiology. Berkeley: University of California Press; 2005. pp. 201–228.
23. Paradis, E., Claude, J., Strimmer, K. 2004 APE: analuses of phylogenetics and evolution in R language. Bioinformatics. 20, 289-290.
24. Revell, L. J. 2012 phytools: an R package for phylogenetic comparative biology (and other things). Methods in Ecology and Evolution. 3, 217-223.
25. Felsenstein J. Phylogenies and the comparative method. *The American Naturalist* 1985; 125, 1-15.
26. Sereno PC, Wilson JA, Witmer LM, Whitlock JA, Maga A et al. Structural extremes in a Cretaceous dinosaur. PLoS ONE 2007; 2: e1230.
27. Curry Rogers KA. Titanosauria: a phylogenetic overview. In: Curry Rogers KA, Wilson JA, editors. The sauropods: evolution and paleobiology. Berkeley; University of California Press; 2005. pp 50–103.
28. Preuschoft H, Hohn B, Stoinski S, Witzel U. 2011. Why so huge? Biomechanical reasons for the acquisition of large size in sauropod and theropod dinosaurs. In *Biology of the sauropod dinosaurs: understanding the life of giants* (eds Klein N, Remes K, Gee CT, Sander PM), pp. 197-218. Bloomington, IN: Indiana University Press.
29. Button DJ, Rayfield EJ, Barrett PM. Cranial biomechanics underpins high sauropod diversity in resource-poor environments *Proc. Roy. Soc. B* 2014; doi: 10.1098/rspb.2014.2114.
30. Young MT, Rayfield EJ, Holliday CM, Witmer LM, Button DJ, Upchurch P, Barrett PM. Cranial biomechanics of *Diplodocus* (Dinosauria, Sauropoda): testing hypotheses of feeding behaviour in an extinct megaherbivore. Naturwissenschaften 2012; 99, 637–643.
31. Calvo JO. 1994 Jaw mechanics in sauropod dinosaurs. Gaia 10, 183 – 193.
32. Christiansen P. 2000 Feeding mechanisms of the sauropod dinosaurs Brachiosaurus, Camarasaurus, Diplodocus and Dicraeosaurus. Hist. Biol. 14, 137 – 152. (doi:10.1080/10292380009380563)
33. Upchurch P, Barrett PM. 2000 The evolution of sauropod feeding mechanisms. In Evolution of herbivory in terrestrial vertebrates: perspectives from the fossil record (ed. H-D Sues), pp. 79 – 122. Cambridge, UK: Cambridge University Press.
34. Mallison H. 2011. Rearing giants: Kinetic-dynamic modeling of sauropod bipedal and tripodal poses. In *Biology of the sauropod dinosaurs: understanding the life of giants* (eds Klein N, Remes K, Gee CT, Sander PM), pp. 237–250. Bloomington, IN: Indiana University Press.
35. Hutchinson JR. Biomechanical modeling and sensitivity analysis of bipedal running. I. Extant taxa. *J. Morph*. 2004; 262, 421-440.
36. Mannion PD, Upchurch P. A quantitative analysis of environmental associations in sauropod dinosaurs. *Paleobiology* 2010; 36: 253-282.
37. Wilson JA, Carrano MT. Titanosaurs and the origin of “wide-gauge‟ trackways: a biomechanical systematic perspective on sauropod locomotion. *Paleobiology* 1999; 25, 252-267.
38. Wilson JA. Integrating ichnofossil and body fossil records to estimate locomotor posture and spatiotemporal distribution of early sauropod dinosaurs: a stratocladistic approach. *Paleobiology* 2005; 31, 400-423.
39. Salgado L, García R, Variación morfológica en la secuencia de vértebras caudales de algunos saurópodos titanosaurios. *Revista Española de Paleontología* 2002; 17, 211-216.
40. Ibiricu LM, Lamanna MC, Lacovara KJ. The influence of caudofemoral musculature on the titanosaurian (Saurischia: Sauropoda) tail skeleton: morphological and phylogenetic implications. *Historical* *Biology* 2014; 26, 454-471.
41. Borsuk-Bialynicka M. A new camarasaurid sauropod *Opisthocoelicaudia skarzynskii* gen. n., sp. n. from the Upper Cretaceous of Mongolia. *Palaeontologica* *Polonica* 1977; 37, 5-63.
42. Otero A. The appendicular skeleton of *Neuquensaurus*, a Late Cretaceous saltasaurine sauropod from Patagonia, Argentina. *Acta* *Palaeontologica* *Polonica* 2010; 55, 399-426.
43. Owaki D, Kano T, Nagasawa K, Tero A, Ishiguro A. Simple robot suggests physical interlimb communication is essential for quadruped walking. *J. Roy. Soc. Interface* 2013; 0120669.
44. P. J. Rose, *Palaeontologica Electronica* **10**, 1 (2007).
45. M. D. D’Emic, *Journal of Systematic Palaeontology* **11**, 707 (2013).
46. D’Emic MD. The early evolution of titanosauriform sauropod dinosaurs. *Zoological Journal of the Linnean Society* 2012; 166: 624–671.
47. L. K. Gabunia *et al.*, *Journal of Vertebrate Paleontology* **18**, 233 (1998).
48. M. P. Taylor, *Journal of Vertebrate Paleontology* **29**, 787 (2009).
49. P. Upchurch *et al.*, *Special Papers in Palaeontology* **77**, 57 (2007).
50. Remes K, Ortega F, Fierro I, Joger U, Kosma R, Ferrer JMM, PALDES, SNHM, Ide OA, Maga A. A new basal sauropod dinosaur from the Middle Jurassic of Niger and the early evolution of Sauropoda. *PLoS ONE* 2009; 4: e6924.
51. Carballido JL, Sander PM. Postcranial axial skeleton of *Europasaurus holgeri* (Dinosauria, Sauropoda) from the Upper Jurassic of Germany: implications for sauropod ontogeny and phylogenetic relationships of basal Macronaria. *Journal of Systematic Palaeontology* 2014; 12: 335–387.
52. Holwera F, Rauhut O and Pol D. Osteological revision of the holotype of *Patagosaurus fariasi*, a basal eusauropod from Argentina. *Journal of Vertebrate Paleontology* 2014; 34, supplement 2, 149A.
53. Lauren M. The evolution of body size, cope’s rule and the origin of amniotes. Systematic Biology 2004; 53: 594-622.
54. Sallan LC, Friedman M. Heads or tails: staged diversification in vertebrate evolutionary radiations. *Proceedings of the Royal Society of London B* 2012; 279:2025–2032.
55. Brusatte SL, Lloyd GT, Wang SC, Norell MA. Gradual assembly of avian body plan culminated in rapid rates of evolution across dinosaur-bird transition. *Current Biology* 2014; 24: 2386-2392.
56. Bapst DW. Assessing the effect of time-scaling methods on phylogeny-based analyses in the fossil record. *Paleobiology* 2014; 40: 331-351.
57. Hutchinson JR, Ng-Thow-Hing V, Anderson FC. A 3D interactive method for estimating body segmental parameters in animals: application to the turning and running performance of *Tyrannosaurus rex*. *Journal of Theoretical Biology* 2007; 246: 660-680.
58. Hutchinson JR, **Bates KT,** Molnar J, Allen V, Makovicky P. A computational and comparative analysis of limb and body proportions in Tyrannosaurus rex with implications for locomotion and growth. PLoS ONE 2011; 6(10): e26037. doi:10.1371/journal.pone.0026037.
59. K. A. Stevens, M. J. Parrish, In Thunder-lizards: The Sauropodomorph Dinosaurs. Virginia Tidwell and Kenneth Carpenter Eds. (Indiania University Press, Bloomington), pp. 212-232 (2005).
60. Alexander RM. Dinosaur biomechanics, *Proceedings of the Royal Society of London B* 2006; 273:1849-1855.
61. Mannion PD, Upchurch P. Completeness metrics and the quality of the sauropodomorph fossil record through geological and historical time. *Paleobiology* 2010; 36: 283–302.
62. K. J. Lacovara et al., *Scientific Reports* **4**, 6196 (2014).
63. Sander PM. The Norian *Plateosaurus* bonebeds of central Europe and their taphonomy. *Paleogeography, Paleoclimatology, Paleoecology* 1992; 93: 255–299.
64. Barrett PM, Upchurch P, Wang X-L. Cranial osteology of *Lufengosaurus huenei* Young (Dinosauria: Prosauropoda) from the Lower Jurassic of Yunnan, People's Republic of China. *Journal of Vertebrate Paleontology* 2005; 25: 806–822.
65. Upchurch P, Martin J. The Rutland *Cetiosaurus*: the anatomy and relationships of a Middle Jurassic British sauropod dinosaur. *Palaeontology* 2002; 45: 1049–1074.
66. Martin J. A Task of Dinosaur Proportions. Collecting, Conserving and Exhibiting the "Rutland Dinosaur". *Natural Science Conservation Group Newsletter* 1999; 12: 20–26.
67. Upchurch P, Martin J. The anatomy and taxonomy of *Cetiosaurus* (Saurischia: Sauropoda) from the Middle Jurassic of England. *Journal of Vertebrate Paleontology* 2003; 23: 208–231.
68. Bonaparte J. F. Les dinosaures (Carnosaures, Allosauridés, Sauropodes, Cétosauridés) du Jurassique Moyen de Cerro Cóndor (Chubut, Argentina). Annales de Paléontologie (Vert.-Invert.) 1986; 72: 325–386.
69. H. Ouyang and Y. Ye. The first mamenchisaurian skeleton with complete skull: Mamenchisaurus youngi. *Sichuan Science and Technology Press, Chengdu* 2002; 1-111.
70. Sereno PC, Beck AL, Dutheil DB, Larssen HCE, Lyon GH, Moussa B, Sadleir RW, Sidor CA, Varricchio DJ, Wilson GP, Wilson JA. Cretaceous sauropods from the Sahara and the uneven rate of skeletal evolution among dinosaurs. *Science* 1999; 286: 1342–1347.
71. Gilmore CW. Osteology of *Apatosaurus* with special reference to specimens in the Carnegie Museum. *Memoirs of the Carnegie Museum* 1936; 11: 175–300.
72. McIntosh JS. Annotated catalogue of the dinosaurs (Reptilia, Archosauria) in the collections of Carnegie Museum of Natural History. Bulletin of Carnegie Museum of Natural History 1981; 18: 1–67.
73. Tschopp E, Mateus O and Benson RBJ. A specimen-level phylogenetic analysis and taxonomic revision of Diplodocidae (Dinosauria, Sauropoda). PeerJ 2015; 3: e857.
74. Salgado L, Bonaparte JF. Un nuevo saurópodo Dicraeosauridae, *Amargasaurus cazaui* gen. et sp. nov., de la Formacion La Amarga, Neocomiano de la provincia del Neuquen, Argentina. *Ameghiniana* 1991; 28: 333–346.
75. Heinrich W-D. The taphonomy of dinosaurs from the Upper Jurassic of Tendaguru (Tanzania) based on field sketches of the German Tendaguru Expedition (1909–13). *Mitteilungen aus dem Museum für Naturkunde Berlin, Geowissenschaftliche Reihe* 1999; 2: 25–61.
76. Curry Rogers KA. The postcranial osteology of *Rapetosaurus krausei* (Sauropoda: Titanosauria) from the Late Cretaceous of Madagascar. *Journal of Vertebrate Paleontology* 2009; 29: 1046–1086.
77. Powell JE. Revision of South American titanosaurid dinosaurs: palaeobiological, palaeobiogeographical and phylogenetic aspects. Records of the Queen Victoria Museum 2003; 111: 1–173.
78. Huene, F. von. Los saurisquios y ornitisquios del Cretácico Argentino. *Museo de La Plata, Anales* 1929; 3: 1–194.
79. D’Emic MD, Wilson JA. New remains attributable to the holotype of *Neuquensaurus australis* (Dinosauria: Sauropoda): implications for saltasaurine systematics. Acta Palaeontologia Polonica 2011; 56: 61–73.
80. Hone DWE. Variation in the tail length of non-avian dinosaurs. *Journal of Vertebrate Paleontology* 2012; 32: 1082-1089.
81. J. O. Calvo *et al.*, *Arquivos do Museu Nacional, Rio de Janeiro* **65**, 511 (2008).
82. R. J. Butler *et al.*, *BMC Evolutionary Biology* **14**, 128 (2014).
83. S. J. Nesbitt *et al.*, *Biology Letters* **9**, 1 (2013).
84. S. J. Nesbitt, *Bulletin of the American Museum of Natural History***353**, 1 (2011).
85. D. B. Norman *et al.*, *Zoological Journal of the Linnean Society* **163**, 182 (2011).
86. R. N. Martinez *et al.*, *Science* **331**, 206 (2011).
87. R. Cúneo *et al.*, *Gondwana Research* **24**, 1267 (2013).
88. O. W. M. Rauhut, A. López-Arbarello, *Palaeogeography, Palaeoclimatology, Palaeoecology* **271**, 259 (2009).
89. L. K. Gabunia *et al.*, *Journal of Vertebrate Paleontology* **18**, 233 (1998).
90. S. Apesteguía, *Gondwana Research* **12**, 533 (2007).
91. P. D. Mannion *et al.*, *Zoological Journal of the Linnean Society* **168**, 98 (2013).
92. K. A. Curry Rogers, C. A. Forster, *Nature* **412**, 530 (2001).
